# Development of a quantitative proteomics approach for cyclooxygenases and lipoxygenases in parallel to quantitative oxylipin analysis allowing the comprehensive investigation of the arachidonic acid cascade

**DOI:** 10.1007/s00216-022-04489-3

**Published:** 2023-01-23

**Authors:** Nicole M. Hartung, Malwina Mainka, Rebecca Pfaff, Michael Kuhn, Sebastian Biernacki, Lilli Zinnert, Nils Helge Schebb

**Affiliations:** Chair of Food Chemistry, Faculty of Mathematics and Natural Sciences, University of Wuppertal, Gaußstr. 20, 42119 Wuppertal, Germany

**Keywords:** Targeted proteomics, Targeted oxylipin metabolomics, Arachidonic acid cascade, Liquid chromatography tandem mass spectrometry, Multiple reaction monitoring cubed, Human macrophages

## Abstract

Oxylipins derived from the cyclooxygenase (COX) and lipoxygenase (LOX) pathways of the arachidonic acid (ARA) cascade are essential for the regulation of the inflammatory response and many other physiological functions. Comprehensive analytical methods comprised of oxylipin and protein abundance analysis are required to fully understand mechanisms leading to changes within these pathways. Here, we describe the development of a quantitative multi-omics approach combining liquid chromatography tandem mass spectrometry–based targeted oxylipin metabolomics and proteomics. As the first targeted proteomics method to cover these pathways, it enables the quantitative analysis of all human COX (COX-1 and COX-2) and relevant LOX pathway enzymes (5-LOX, 12-LOX, 15-LOX, 15-LOX-2, and FLAP) in parallel to the analysis of 239 oxylipins with our targeted oxylipin metabolomics method from a single sample. The detailed comparison between MRM^3^ and classical MRM-based detection in proteomics showed increased selectivity for MRM^3^, while MRM performed better in terms of sensitivity (LLOQ, 16–122 pM vs. 75–840 pM for the same peptides), linear range (up to 1.5–7.4 μM vs. 4–368 nM), and multiplexing capacities. Thus, the MRM mode was more favorable for this pathway analysis. With this sensitive multi-omics approach, we comprehensively characterized oxylipin and protein patterns in the human monocytic cell line THP-1 and differently polarized primary macrophages. Finally, the quantification of changes in protein and oxylipin levels induced by lipopolysaccharide stimulation and pharmaceutical treatment demonstrates its usefulness to study molecular modes of action involved in the modulation of the ARA cascade.

**Graphical Abstract:**

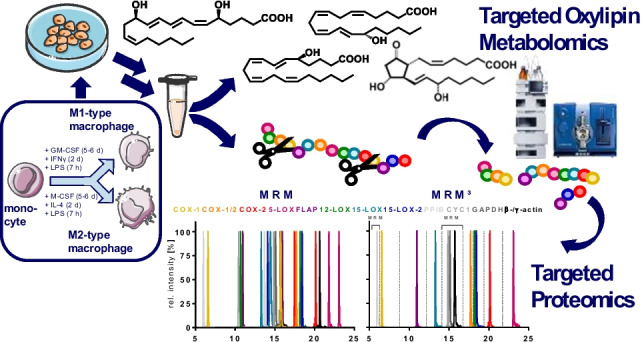

**Supplementary Information:**

The online version contains supplementary material available at 10.1007/s00216-022-04489-3.

## Introduction

**Fig. 1:**
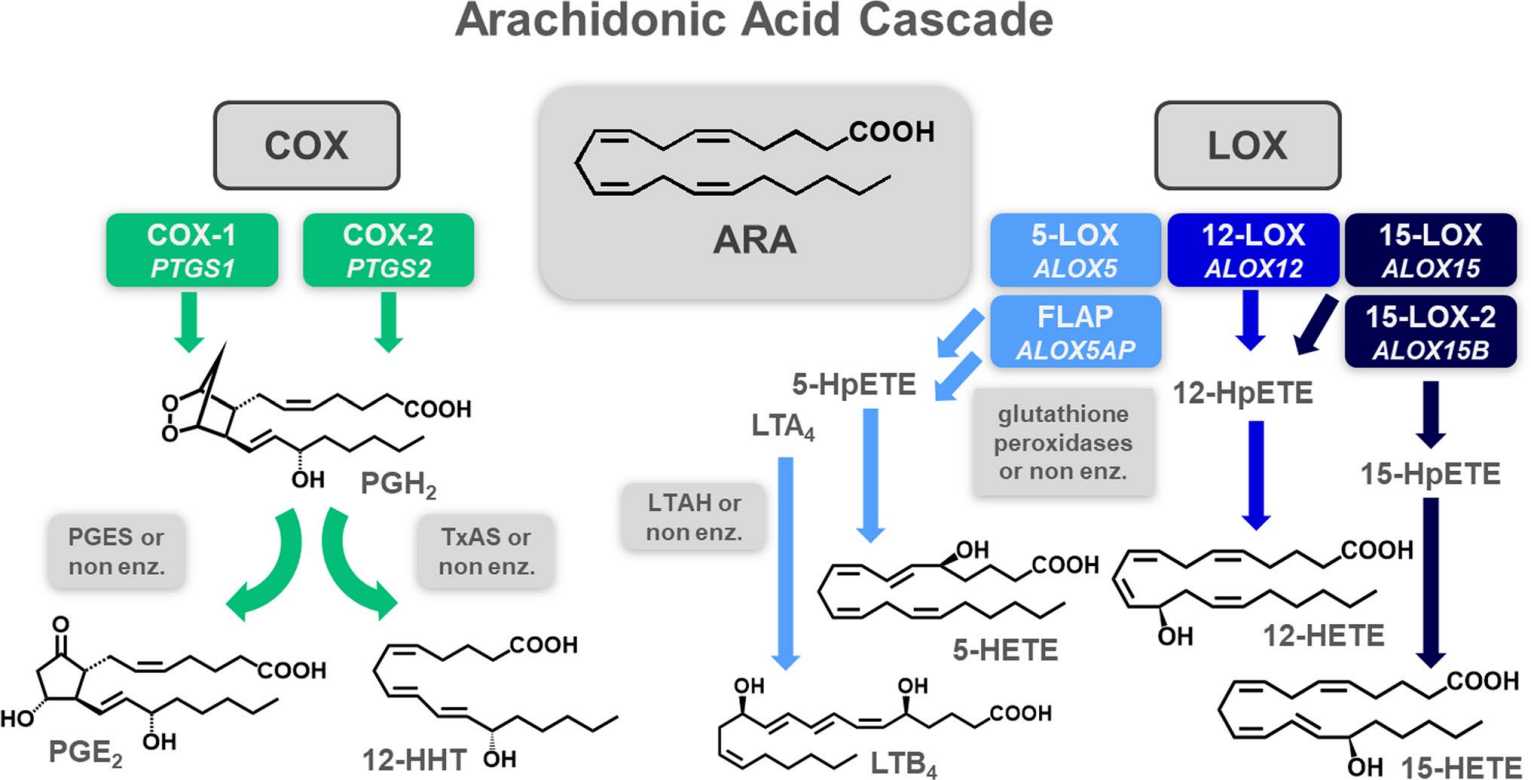
Simplified overview of the cyclooxygenase (COX) and lipoxygenase (LOX) branches of the arachidonic acid (ARA) cascade. COX catalyzes the formation of prostaglandin (PG) H_2_ which is further converted by downstream enzymes or non-enzymatically, e.g., to PGE_2_ by PGE synthases (PGES) or to 12-hydroxy-heptadecatrienoic acid (12-HHT) by thromboxane A synthase (TxAS). The different LOX isoforms each oxidize ARA regiospecifically to hydroperoxy-eicosatetraenoic acids (HpETE) or leukotriene A_4_ (LTA_4_) in case of 5-LOX supported by the 5-LOX-activating protein (FLAP). The primary products are reduced to their respective hydroxy eicosatetraenoic acids (HETE) by, e.g., glutathione peroxidases or rapidly hydrolyzed to LTB_4_ in case of LTA_4_ (gene names are noted under the enzyme/protein names in italic)

The cyclooxygenase (COX) and lipoxygenase (LOX) pathways of the arachidonic acid (ARA) cascade play important roles in inflammation (simplified overview in Fig. 1). The formed eicosanoids and other oxylipins are potent lipid mediators of the immune response [1]. Through the initial oxidation of polyunsaturated fatty acids, such as ARA, via one of the two COX enzymes, the unstable prostaglandin (PG) H_2_ is formed and can be further converted by downstream enzymatic or non-enzymatic reactions, e.g., to PGE_2_ or 12-hydroxy-heptadecatrienoic acid (12-HHT) [2, 3]. Formed in immune cells, PGE_2_ acts as a pro-inflammatory signaling molecule by, e.g., stimulating the upregulation of pro-inflammatory cytokines or enhancing blood flow through augmented atrial vasodilation [4, 5]. Increased PGE_2_ levels are often associated with upregulated COX-2 (derived from the *PTGS2* gene) abundance that is induced by pro-inflammatory stimuli such as gram-negative bacteria [5]. Though biological functions of 12-HHT are not yet fully understood, recent studies have found this oxylipin to be involved i.a. in the mediation of allergic inflammation [6]. As chemical breakdown product of PGH_2_, it is an established marker of COX activity [7]. The several LOX isoforms catalyze the stereo- and regiospecific formation of hydroperoxy fatty acids as primary products that are — in the cell — rapidly reduced to hydroxy fatty acids, e.g., hydroxyeicosatetraenoic acids (HETE) formed from ARA [8]. The LOX branch of the ARA cascade is also involved in inflammation regulation. 5-LOX catalyzes the formation of pro-inflammatory and chemotactic leukotrienes (LT), such as ARA-derived LTB_4_. The multiple hydroxylated fatty acids formed via consecutive LOX activity are believed to elicit anti-inflammatory properties involved in the active resolution of inflammation [8, 9] but remain controversially discussed [10]. The multitude of products arising from the many ARA cascade enzymes, crosstalk between the different branches, and various structurally distinct fatty acid substrates make a comprehensive oxylipin metabolomics platform necessary for thorough investigation of the oxylipin pattern. However, in order to fully comprehend the mechanisms leading to changes on metabolite levels, the additional investigation of gene expression, i.e., protein abundance, is indispensable.

In the recent years, interest in multi-omics techniques as tools to achieve systemic understanding of biological changes has drastically increased, i.e., metabolomics, proteomics, and transcriptomics [11, 12]. While liquid chromatography (LC) tandem mass spectrometry (MS/MS) is the standard method for quantitative targeted oxylipin analysis [13], the LC–MS/MS-based analysis of proteins has emerged in the recent years and is often conducted as high-throughput screenings allowing only relative quantification. Though the investigation of ARA cascade enzymes with proteomic tools has been reported [14–18], also in combination with metabolomics analyses [19, 20], a method for its quantitative analysis has not yet been described. Therefore, it was our goal to develop a targeted proteomics method comprising the important COX- and LOX-mediated signaling pathways and, together with our existing targeted oxylipin metabolomics platform [21–23], establishing a comprehensive and quantitative multi-omics tool to thoroughly investigate the ARA cascade.

Our targeted proteomics approach allows the analysis of human COX and LOX enzymes for the first time in a quantitative manner and, together with our oxylipin metabolomics method, is a valuable tool to characterize the ARA cascade from a single sample. This is demonstrated by characterizing the COX and LOX pathways in different human immune cells, showing correlations between oxylipin and protein abundances as well as quantitative changes upon pharmacological intervention.

## Materials and methods

### Chemicals and biological material

Fetal calf serum (superior standardized) was purchased from Biochrom (Berlin, Germany); 1,25-dihydroxyvitamin D_3_ (VD_3_) and ML351 as well as oxylipin standards were purchased from Cayman Chemical (Ann Arbor, MI, USA; local supplier Biomol, Hamburg, Germany). HEK293 cell-derived recombinant human transforming growth factor-β1 (TGF-β1), recombinant human colony-stimulating factors CSF-1 (M-CSF), CSF-2 (GM-CSF), interferon γ (IFNγ), and interleukin 4 (IL-4) produced in *Escherichia coli* were obtained from PeproTech Germany (Hamburg, Germany). Lymphocyte separation medium was purchased at PromoCell (Heidelberg, Germany). Human AB serum was provided by the blood donation center University Hospital Düsseldorf (Düsseldorf, Germany). Protease inhibitor mix M (AEBSF, Aprotinin, Bestatin, E-64, Leupeptin and Pepstatin A) and resazurin as well as MS approved trypsin (> 6.000 U g^−1^, from porcine pancreas) were from SERVA Electrophoresis GmbH (Heidelberg, Germany). Unlabeled AQUA peptide standards were obtained from Thermo Life Technologies GmbH (Darmstadt, Germany), unlabeled and heavy labeled (lys, uniformly labeled (U)-^13^C_6_; U-^15^N_2_; arg, U-^13^C_6_; U-^15^N_4_) peptide standards were purchased from JPT Peptides (Berlin, Germany).

Acetonitrile (HPLC–MS grade), acetone (HPLC grade), methanol, and acetic acid (both Optima LC–MS grade) were obtained from Fisher Scientific (Schwerte, Germany). Dithiothreitol was from AppliChem (Darmstadt, Germany). Tris(hydroxymethyl)aminomethane (TRIS), ammonium bicarbonate, sodium deoxycholate, and urea were obtained from Carl Roth (Karlsruhe, Germany). RPMI 1640, l-glutamine, and penicillin/streptomycin (5000 units penicillin and 5 mg streptomycin mL^−1^), lipopolysaccharide (LPS) from *E. coli* (0111:B4), dextran 500 from *Leuconostoc *spp., iodoacetamide, dimethylsulfoxide (DMSO), dexamethasone, indomethacin, celecoxib, and PF-4191834 as well as all other chemicals were purchased from Sigma (Schnellendorf, Germany).

### Cell cultivation

THP-1 cells were obtained from the German Collection of Microorganisms and Cell Cultures GmbH (DSMZ, Braunschweig, Germany) and were maintained in bicarbonate buffered RPMI medium supplemented with 10% fetal calf serum, 100 U mL﻿^−1^ penicillin, 100 μg mL^−1^ streptomycin (P/S, 2%) and 2 mM l-glutamine (1%) in 60.1 cm^2^ dishes in a humidified incubator at 37 °C and 5% CO_2_. For experiments, cells were seeded at densities of 0.125·10^6^ cells mL^−1^ and differentiated with 50 nM VD_3_ (0.1% DMSO) and 1 ng mL^−1^ TGF-β1 for 72 h.

Primary human macrophages were prepared as described by [24]. In brief, peripheral blood monocytic cells (PBMC) were isolated from buffy coats obtained from blood donations at the University Hospital Düsseldorf. Blood samples were drawn with the informed consent of the human subjects. The study was approved by the Ethical Committee of the University of Wuppertal. PBMC were isolated by dextran (5%) sedimentation for 45 min and subsequent centrifugation (1000 × *g* without deceleration, 10 min, 20 °C) on lymphocyte separation medium. The leucocyte ring was isolated and washed twice with PBS. Cells were seeded in 60.1 cm^2^ dishes and left to adhere for 1 h after resuspension in serum-free RPMI medium (2% P/S, 1% l-glutamine) in a humidified incubator at 37 °C and 5% CO_2_ (8 dishes per donor). Cells were washed, and RPMI medium (2% P/S, 1% l-glutamine) supplemented with 5% human AB serum was added. For polarization towards M1- or M2-like macrophages, the medium was additionally supplemented with 10 ng mL^−1^ CSF-2 or CSF-1 for 8 days and treated with 10 ng mL^−1^ IFNγ or IL-4 for the final 48 h. No cytokines were added to generate M0-like macrophages.

Platelets were isolated from EDTA blood as described by the platelet-rich plasma method [25].

### Cell culture experiments

For the experiments of the THP-1 cells or primary macrophages with test compounds, cell culture medium was replaced 7 h before the end of the differentiation with serum-free 50 mM TRIS-buffered RPMI medium (2% P/S, 1% l-glutamine) and the pharmacological inhibitors or DMSO (0.1%) as control were added. Cytotoxic effects of the test compounds at the used concentrations were excluded by resazurin (Alamar Blue) assay [26] and lactate dehydrogenase assay (ESM Figs. S4 and S5). After 1 h of preincubation, cells were additionally treated with 1 μg mL^−1^ LPS for 6 h. In case of the THP-1 cells, all adherent and non-adherent cells were harvested by scraping in the cell culture medium. Primary macrophages were harvested by cold shock method [24]. The harvested cell pellets were frozen at − 80 °C until use.

### Quantification of oxylipin and protein levels by LC–MS/MS

The presented methods allow the quantitative analysis of 239 oxylipins (ESM Table S4) and 11 proteins (Tables 1 and ﻿2, ESM Table S7) from one cell pellet. Cells were resuspended in PBS containing 1% protease inhibitor mix and antioxidant solution (0.2 mg m^-1^L BHT, 100 μM indomethacin, 100 μM soluble epoxide hydrolase inhibitor *trans*-4-[4-(3-adamantan-1-yl-ureido)-cyclohexyloxy]-benzoic acid (*t*-AUCB) in MeOH) [21, 22] and sonicated, and protein content was determined via bicinchoninic acid assay [27]. Internal standards (IS) for oxylipin analysis were added to the cell lysate before proteins were precipitated in methanol at − 80 °C for at least 30 min. The supernatant after centrifugation (20000 × *g*, 10 min, 4 °C) served as sample for oxylipin analysis, while the protein levels were later separately analyzed in the precipitated protein pellet after storage at − 80 °C. For the oxylipin analysis, the supernatant after the protein precipitation was further purified according to the previously published method [21, 22] by solid-phase extraction on a non-polar (C8)/strong anion exchange mixed mode material (Bond Elut Certify II, 200 mg, Agilent, Waldbronn, Germany) and analyzed by LC–MS/MS. For the targeted LC–MS/MS-based proteomics analysis, the protein pellet obtained after the protein precipitation was resuspended in 5% (*w/v*) sodium deoxycholate containing 1% protease inhibitor mix and precipitated again in four volumes of ice-cold acetone after centrifugation (15000 × *g*, 20 min, 4 °C). Further steps were carried out as described by [18]. In brief, the dried protein pellet was re-dissolved in 6 M urea, the disulfide bridges were reduced with dithiothreitol, and the resulting free sulfhydryl groups were alkylated with iodoacetamide in order to inhibit the reformation of disulfide bridges. The samples were diluted with 50 mM NH_4_HCO_3_ before the tryptic digestion was carried out at a trypsin-to-protein ratio of 1:50. The digestion was stopped after 15 h by adding concentrated acetic acid to reduce the pH from ≈ 7.8 to 3–4. A mixture of heavy labeled peptides (lys, U-^13^C_6_; U-^15^N_2_; arg, U-^13^C_6_; U-^15^N_4_) corresponding to each of the analytes was spiked as internal standards (final vial concentrations, 25 nM for COX and LOX peptides and 50/100 nM for the housekeeper peptides), before the samples were subjected to solid-phase extraction (Strata-X 33 μm Polymeric Reversed Phase, Phenomenex LTD, Aschaffenburg, Germany) and analyzed by LC–MS/MS.

**Table 1:** MRM method parameters for (A) unlabeled and (B) heavy labeled (lys, U-^13^C_6_; U-^15^N_2_; arg, U-^13^C_6_; U-^15^N_4_) peptides of COX-1, COX-2, 5-LOX, FLAP, 12-LOX, 15-LOX, and 15-LOX-2 used as internal standards (IS)

**(A)**													
**Gene/protein (UniProtKB No.)**	**Peptide**	**Transitions**	**Q1** ***m/z***	**Q3** ***m/z***	**RT [min]**	**Rel. ratio to quantifier [%]**	**CE (V)**	**IS transitions**	**Calibration range [nM]**	**LOD [pM]**	**LLOQ [pM]**	**LOD peptide on column [fg]**	**LOD enzyme on column [pg]**
**PTGS1/cyclooxygenase-1 (COX-1; P23219)**	**DCPTPMGTK**	M^2+^ → y_7_^+^	503.7	731.4			19						
		M^2+^ → b_2_^+^	503.7	276.1	6.92 ± 0.01	59	20	M^2+^ → y_7_^+^	0.016–1570	7.9	16	37	2.7
		M^2+^ → y_5_^+^	503.7	533.3		43	31						
	**AEHPTWGDEQLFQTTR**	M^3+^ → y_5_^+^	639.3	652.3			26						
		M^3+^ → y_4_^+^	639.3	505.3	16.06 ± 0.03	57	28	M^3+^ → y_5_^+^	0.50–5000	250	500	2394	86
		M^3+^ → y_6_^+^	639.3	765.4		55	28						
**PTGS2/cyclooxygenase-2 (COX-2; P35354)**	**FDPELLFNK**	M^2+^ → y_7_^++^	561.8	430.7			25						
		M^2+^ → y_7_^+^	561.8	860.4	20.44 ± 0.02	36	25	M^2+^ → y_7_^++^	0.021–2111	4.2	21	24	1.5
		M^2+^ → b_2_^+^	561.8	263.1		25	24						
	**NAIMSYVLTSR**	M^2+^ → y_8_^+^	627.8	956.3			29						
		M^2+^ → b_3_^+^	627.8	299.1	17.81 ± 0.02	86	27	M^2+^ → y_8_^+^	0.25–5000	100	250	627	34
		M^2+^ → y_9_^+^	627.8	1069.6		43	27						
**PTGS1/COX-1 & PTGS2/COX-2**	**LILIGETIK**	M^2+^ → y_7_^+^	500.3	773.3			23						
		M^2+^ → b_2_^+^	500.3	227.2	18.03 ± 0.02	62	22	M^2+^ → y_7_^+^	0.027–2660	13	27	66	4.6
		M^2+^ → y_5_^+^	500.3	547.3		30	25						
**ALOX5/5-lipoxygenase (5-LOX; P09917)**	**DDGLLVWEAIR**	M^2+^ → y_6_^+^	643.8	773.4			30						
		M^2+^ → y_7_^+^	643.8	886.5	23.38 ± 0.01	81	28	M^2+^ → y_6_^+^	0.122–1219	49	122	313	19
		M^2+^ → y_5_^+^	643.8	674.4		85	25						
	**NLEAIVSVIAER**	M^2+^ → y_7_^+^	657.4	773.5			28						
		M^2+^ → y_10_^+^	657.4	1086.6	22.12 ± 0.01	66	30	M^2+^ → y_6_^+^	0.25–5000	100	250	656	39
		M^2+^ → y_8_^+^	657.4	886.5		43	30						
**ALOX5AP/arachidonate 5-lipoxygenase-activating protein (FLAP; P20292)**	**TGTLAFER**	M^2+^ → y_5_^+^	447.7	635.4			22						
		M^2+^ → y_3_^+^	447.7	451.2	11.30 ± 0.02	70	24	M^2+^ → y_4_^+^	0.074–7366	37	74	165	3.4
		M^2+^ → y_6_^+^	447.7	736.4		55	20						
	**YFVGYLGER**	M^2+^ → y_7_^+^	552.3	793.4			24						
		M^2+^ → b_2_^+^	552.3	311.1	16.27 ± 0.03	67	24	M^2+^ → y_7_^+^	0.010–5000	5.0	10	28	0.45
		M^2+^ → y_6_^+^	552.3	694.4		69	26						
**ALOX12/12-lipoxygenase (12-LOX; P18054)**	**LWEIIAR**	M^2+^ → y_5_^+^	450.8	601.4			21						
		M^2+^ → b_2_^+^	450.8	300.2	18.51 ± 0.02	32	17	M^2+^ → y_6_^+^	0.025–5000	10	25	45	3.8
		M^2+^ → y_6_^+^	450.8	787.4		21	21						
	**AVLNQFR**	M^2+^ → y_5_^+^	424.2	677.4			19						
		M^2+^ → y_4_^+^	424.2	564.3	11.07 ± 0.02	47	21	M^2+^ → y_5_^+^	0.050–5000	25	50	106	9.5
		M^2+^ → y_3_^+^	424.2	450.3		6	19						
**ALOX15/15-lipoxygenase (15-LOX; P16050)**	**EITEIGLQGAQDR**	M^2+^ → y_8_^+^	715.4	844.4			34						
		M^2+^ → y_5_^+^	715.4	546.3	13.62 ± 0.01	38	32	M^2+^ → y_8_^+^	0.113–5629	56	113	402	21
		M^2+^ → y_9_^+^	715.4	957.5		29	35						
	**GFPVSLQAR**	M^2+^ → y_7_^++^	487.8	385.7			20						
		M^2+^ → y_5_^+^	487.8	574.3	14.78 ± 0.01	28	29	M^2+^ → y_7_^++^	0.25–5000	100	250	487	37
		M^2+^ → y_7_^+^	487.8	770.5		18	24						
**ALOX15B/15-lipoxygenase-2 (15-LOX-2; O15296)**	**ELLIVPGQVVDR**	M^2+^ → y_7_^+^	669.4	770.4			30						
		M^2+^ → b_5_^+^	669.4	568.4	18.76 ± 0.02	32	24	M^2+^ → y_7_^+^	0.044–4391	22	44	147	8.3
		M^2+^ → y_8_^+^	669.4	869.5		32	29						
	**VSTGEAFGAGTWDK**	M^2+^ → y_7_^+^	713.3	734.3			36						
		M^2+^ → y_8_^+^	713.3	881.4	14.42 ± 0.02	83	36	M^2+^ → y_7_^+^	0.250–5000	100	250	712	38
		M^2+^ → y_9_^+^	713.3	952.5		79	35						
**(B)**													
**Gene/protein (UniProtKB No.)**	**Peptide**	**Transitions**	**Q1 *m/z***	**Q3 *m/z***	**RT [min]**	**Rel. ratio to quantifier [%]**	**CE (V)**						
**PTGS1/cyclooxygenase-1 (COX-1; P23219)**	**DCPTPMGTK**	M^2+^ → y_7_^+^	507.7	739.4			19						
		M^2+^ → b_2_^+^	507.7	276.1	6.92 ± 0.01	59	20						
		M^2+^ → y_7_^++^	507.7	370.2		17	23						
	**AEHPTWGDEQLFQTTR**	M^3+^ → y_5_^+^	642.6	662.4			26						
		M^3+^ → y_4_^+^	642.6	515.3	16.06 ± 0.03	53	28						
		M^3+^ → y_6_^+^	642.6	775.4		50	28						
**PTGS2/cyclooxygenase-2 (COX-2; P35354)**	**FDPELLFNK**	M^2+^ → y_7_^++^	565.8	434.8			25						
		M^2+^ → y_7_^+^	565.8	868.5	20.44 ± 0.02	34	25						
		M^2+^ → y_4_^+^	565.8	529.3		6	33						
	**NAIMSYVLTSR**	M^2+^ → y_8_^+^	632.8	966.3			29						
		M^2+^ → b_3_^+^	632.8	299.2	17.81 ± 0.02	92	27						
		M^2+^ → y_7_^+^	632.8	835.5		70	30						
**PTGS1/COX-1 & PTGS2/COX-2**	**LILIGETIK**	M^2+^ → y_7_^+^	504.3	781.5			23						
		M^2+^ → y_6_^+^	504.3	668.4	18.03 ± 0.02	23	24						
		M^2+^ → y_8_^+^	504.3	894.6		4	24						
**ALOX5/5-lipoxygenase (5-LOX; P09917)**	**DDGLLVWEAIR**	M^2+^ → y_6_^+^	648.8	783.4			30						
		M^2+^ → y_7_^+^	648.8	896.5	23.38 ± 0.01	78	28						
		M^2+^ → y_5_^+^	648.8	684.4		83	25						
	**NLEAIVSVIAER**	M^2+^ → y_6_^+^	662.4	684.4			28						
		M^2+^ → y_8_^+^	662.4	896.5	22.12 ± 0.01	76	30						
		M^2+^ → y_4_^+^	662.4	498.3		36	28						
**ALOX5AP/arachidonate 5-lipoxygenase-activating protein FLAP; P20292)**	**TGTLAFER**	M^2+^ → y_4_^+^	452.7	532.2			24						
		M^2+^ → y_5_^+^	452.7	645.4	11.30 ± 0.02	44	22						
		M^2+^ → y_3_^+^	452.7	461.2		32	24						
	**YFVGYLGER**	M^2+^ → y_7_^+^	557.3	803.4			24						
		M^2+^ → b_2_^+^	557.3	311.1	16.27 ± 0.03	66	24						
		M^2+^ → y_6_^+^	557.3	704.4		72	26						
**ALOX12/12-Lipoxygenase (12-LOX; P18054)**	**LWEIIAR**	M^2+^ → y_6_^+^	455.8	797.5			21						
		M^2+^ → y_4_^+^	455.8	482.3	18.51 ± 0.02	87	21						
		M^2+^ → y_3_^+^	455.8	369.2		44	21						
	**AVLNQFR**	M^2+^ → y_5_^+^	429.2	687.4			19						
		M^2+^ → y_3_^+^	429.2	460.3	11.07 ± 0.02	7	19						
		M^2+^ → z_4_^+^	429.2	557.3		6	28						
**ALOX15/15-lipoxygenase (15-LOX; P16050)**	**EITEIGLQGAQDR**	M^2+^ → y_8_^+^	720.4	854.4			34						
		M^2+^ → y_5_^+^	720.4	556.3	13.62 ± 0.01	39	32						
		M^2+^ → y_9_^+^	720.4	967.5		30	35						
	**GFPVSLQAR**	M^2+^ → y_7_^++^	492.8	390.7			20						
		M^2+^ → y_5_^+^	492.8	584.3	14.78 ± 0.01	28	29						
		M^2+^ → y_6_^+^	492.8	683.4		10	30						
**ALOX15B/15-lipoxygenase-2 (15-LOX-2; O15296)**	**ELLIVPGQVVDR**	M^2+^ → y_7_^+^	674.4	780.4			30						
		M^2+^ → y_8_^+^	674.4	879.5	18.76 ± 0.02	30	29						
		M^2+^ → b_5_^+^	674.4	568.4		30	24						
	**VSTGEAFGAGTWDK**	M^2+^ → y_7_^+^	717.3	742.4			36						
		M^2+^ → y_8_^+^	717.3	889.4	14.42 ± 0.02	74	36						
		M^2+^ → y_12_^++^	717.3	624.3		58	30						

For each peptide, different collisionally activated dissociation fragment ions used for qualification and quantification (top) with their Q1 and Q3 *m/z* are shown with retention time (RT, mean ± SD, set of *n* = 23 calibrators) and relative ratios to quantifier transition as well as collision energies (CE). For unlabeled peptides (A), the linear calibration range is shown for quantifier transitions as well as the transitions of the corresponding heavy labeled peptides used as internal standards (IS) for quantification, limits of detection (LOD), lower limits of quantification (LLOQ), and LOD of the peptides and enzymes on column. Accuracy of calibrators was within a range of ± 15% (20% for LLOQ). The concentrations of all heavy labeled peptides (IS) in the vial are 25 nM

**Table 2:** MRM^3^ method parameters for (A) unlabeled and (B) heavy labeled (lys, U-^13^C_6_; U-^15^N_2_; arg U-^13^C_6_; U-^15^N_4_) peptides of COX-1, COX-2, 5-LOX, FLAP, 12-LOX, 15-LOX, and 15-LOX-2 used as internal standards (IS)

**(A)**															
**Protein**	**Peptide**	**Mode**	**Transition (Q1 → Q3)**	**Q1** ***m/z***	**Q3** ***m/z***	***m/z*** **of MS**^**3**^ **fragment ions summed for MRM**^**3**^	**Time period [min]**	**RT [min]**	**CE (V)**	**AF2 (V)**	**Calibration range [nM]**	**LOD [nM]**	**LLOQ** **[nM]**	**LOD peptide on column [pg]**	**LOD enzyme on column [pg]**
**PPIB**	**IGDEDVGR**	MRM	*	*	*	-	0.00–6.61	5.99 ± 0.01	*	-	*	-	-	-	-
**COX-1**	**DCPTPMGTK**	MS^3^	M^2+^ → y_7_^+^	503.7	731.4	713.4 (b_7_^+^), 644.3, 695.3, 533.3 (y_5_^+^), 515.3 (y_5_^+^—H_2_O), 567.4, 585.3 (b_6_^+^), 608.3, 677.3, 387.2	6.61–9.10	6.92 ± 0.01	19	0.08	0.079–31	0.031	0.079	0.15	11
**FLAP**	**TGTLAFER**	MS^3^	M^2+^ → y_5_^+^	447.7	635.4	617.3 (b_5_^+^), 416.2, 277.2, 287.3, 600.4, 382.2, 434.3, 522.3 (y_4_^+^), 461.2 (b_4_^+^), 332.2 (b_3_^+^)	9.10–12.45	11.30 ± 0.01	24	0.08	1.5–368	1.1	1.5	4.9	100
**15-LOX**	**EITEIGLQGAQDR**	MS^3^	M^3+^ → y_5_^+^	477.2	546.3	528.3 (b_5_^+^), 511.2, 330.1, 384.1, 401.2, 215.2, 244.1, 290.1 (y_2_^+^), 372.2 (b_4_^+^), 418.2 (y_3_^+^)	12.45–14.40	13.63 ± 0.01	21	0.07	0.84–113	0.56	0.84	4.0	211
**CYC1**	**DVCTFLR**	MRM	*	*	*	-	14.40–17.03	14.85 ± 0.03	*	-	-	-	-	-	-
**GAPDH**	**GALQNIIPASTGAAK**	MRM	*	*	*	-	14.40–17.03	15.09 ± 0.03	*	-	-	-	-	-	-
**β-/γ-actin**	**VAPEEHPVLLTEAPLNPK**	MRM	*	*	*	-	14.40–17.03	15.68 ± 0.04	*	-	-	-	-	-	-
**COX-1/2**	**LILIGETIK**	MS^3^	M^2+^ → y_7_^+^	500.3	773.3	755.5 (b_7_^+^), 609.4, 496.3, 361.2 (y_3_^+^), 310.3, 547.3 (y_5_^+^), 383.3, 451.3, 514.3 (b_5_^+^), 591.4	17.03–18.26	18.03 ± 0.01	23	0.12	0.13–4.0	0.053	0.13	0.27	18
**12-LOX**	**LWEIIAR**	MS^3^	M^2+^ → y_5_^+^	450.8	601.4	583.4 (b_5_^+^), 472.3 (y_4_^+^), 338.3, 342.2, 229.3, 310.3, 359.2 (y_3_^+^), 356.2 (b_3_^+^), 243.1 (b_2_^+^), 409.4	18.26–18.62	18.51 ± 0.01	21	0.07	0.075–25	0.050	0.075	0.23	19
**15-LOX-2**	**ELLIVPGQVVDR**	MS^3^	M^2+^ → y_7_^+^	669.4	770.4	752.4 (b_7_^+^), 283.1(b_3_^+^), 596.4 (b_6_^+^), 382.2 (b_4_^+^), 464.4, 436.6, 365.4, 337.2 (y_6_^++^), 481.3 (b_5_^+^), 587.5	18.62–19.59	18.76 ± 0.01	30	0.13	0.44–22	0.22	0.44	1.5	83
**COX-2**	**FDPELLFNK**	MS^3^	M^2+^ → y_7_^++^	561.8	430.7	634.4 (y_5_ +), 227.1 (b_2_^+^), 521.3 (y_4_^+^), 340.2 (b_3_^+^), 408.2 (y_3_^+^), 763.4 (y_6_^+^), 261.2 (y_2_^+^), 745.3, 697.2, 373.2	19.59–21.90	20.44 ± 0.01	25	0.05	0.084–42	0.042	0.084	0.24	15
**5-LOX**	**DDGLLVWEIAR**	MS^3^	M^2+^ → y_5_^+^	643.8	674.4	359.2 (y_3_^+^), 387.2 (b_3_^+^), 316.1 (b_2_^+^), 656.4 (b_5_^+^), 324.5, 638.4, 612.4, 595.4, 344.4, 510.4	21.90–36.00	23.38 ± 0.01	25	0.11	0.49–122	0.37	0.49	2.4	144
**(B)**															
**Protein**	**Peptide**	**Mode**	**Transition (Q1 → Q3)**	**Q1** ***m/z***	**Q3** ***m/z***	***m/z* of MS**^**3**^** fragment ions summed for MRM**^**3**^	**Time period [min]**	**RT [min]**	**CE (V)**	**AF2 (V)**	**IS concentration in vial [nM]**				
**PPIB**	**IGDEDVGR**	MRM	*	*	*	-	0.00–6.61	5.99 ± 0.01	*	-	50				
**COX-1**	**DCPTPMGTK**	MS^3^	M^2+^ → y_7_^+^	507.7	739.4	652.3, 721.4 (b_7_^+^), 703.3, 387.2, 541.3 (y_5_^+^)	6.61–9.10	6.92 ± 0.01	19	0.08	25				
**FLAP**	**TGTLAFER**	MS^3^	M^2+^ → y_5_^+^	452.7	645.4	627.3 (b_5_^+^), 425.2, 277.2, 287.3, 391.2	9.10–12.45	11.30 ± 0.01	24	0.08	25				
**15-LOX**	**EITEIGLQGAQDR**	MS^3^	M^3+^ → y_5_^+^	480.6	556.3	538.3 (b_5_^+^), 372.2 (b_4_^+^), 521.2, 330.1, 226.1	12.45–14.40	13.63 ± 0.01	21	0.07	25				
**CYC1**	**DVCTFLR**	MRM	*	*	*	-	14.40–17.03	14.85 ± 0.03	*	-	50				
**GAPDH**	**GALQNIIPASTGAAK**	MRM	*	*	*	-	14.40–17.03	15.09 ± 0.03	*	-	50				
**β-/γ-actin**	**VAPEEHPVLLTEAPLNPK**	MRM	*	*	*	-	14.40–17.03	15.68 ± 0.04	*	-	100				
**COX-1/2**	**LILIGETIK**	MS^3^	M^2+^ → y_7_^+^	504.3	781.5	496.3, 451.1, 310.2, 763.5 (b_7_^+^), 555.3 (y_5_^+^)	17.03–18.26	18.03 ± 0.01	23	0.12	25				
**12-LOX**	**LWEIIAR**	MS^3^	M^2+^ → y_5_^+^	455.8	611.3	593.4 (b_5_^+^), 482.3 (y_4_^+^), 338.3, 351.2, 238.2	18.26–18.62	18.51 ± 0.01	21	0.07	25				
**15-LOX-2**	**ELLIVPGQVVDR**	MS^3^	M^2+^ → y_7_^+^	674.4	780.4	283.1 (b_3_^+^), 762.4 (b_7_^+^), 382.2 (b_4_^+^), 365.4, 337.2	18.62–19.59	18.76 ± 0.01	30	0.13	25				
**COX-2**	**FDPELLFNK**	MS^3^	M^2+^ → y_7_^++^	565.8	434.8	642.4 (y_5_^+^), 227.1 (b_2_^+^), 771.4 (y_6_^+^), 529.3 (y_4_^+^), 340.2 (b_3_^+^)	19.59–21.90	20.44 ± 0.01	25	0.05	25				
**5-LOX**	**DDGLLVWEIAR**	MS^3^	M^2+^ → y_5_^+^	648.8	684.4	369.2 (y_3_^+^), 387.2 (b_3_^+^), 648.5, 352, 466.2	21.90–36.00	23.38 ± 0.01	25	0.11	25				

The unlabeled and corresponding heavy labeled peptides from one protein were measured together in one time period covering each retention time (RT). RT are shown as mean ± SD, set of *n* = 19 calibrators. Shown are Q1 *m/z* and collisionally activated dissociation fragments (Q3) as well as selected MS^3^ fragments together with their respective collision (CE) and excitation energies (AF2). The linear trap (LIT) excitation time was set to 25 ms (standard setting) with fixed fill times of 250 ms (maximum) for all peptides (TGTLAFER = 100 ms, IS peptides, 25 ms) at a scan rate of 10 000 Da/s. The MS^3^ fragments were isolated from the MS^3^ spectra with an isolation window of ± 0.5 Da. The ratio between the sum of (A) 10 MS^3^ fragments of the unlabeled peptide and (B) 5 MS^3^ fragments of the heavy labeled peptide is used for quantification. The concentrations of the IS are shown in (B). The linear calibration range and the limits of detection (LOD), lower limits of quantification (LLOQ), and LOD of the peptides and enzymes on column are shown for (A) unlabeled peptides. The accuracy of the calibrators was within a range of ± 15% (± 20% for LLOQ). Additionally, peptides of four housekeeping proteins (GAPDH, PPIB, β-/γ-actin, CYC1) were measured in MRM mode as separate periods with set dwell times of 20 ms and the parameters specified in ESM Table S7

^*^details are specified in ESM Table S7

The samples for the oxylipin and peptide analysis were measured with separate methods on two 1290 Infinity II LC systems, each equipped with a Zorbax Eclipse Plus C18 reversed phase column (2.1 × 150 mm, particle size 1.8 μm, pore size 95 Å, Agilent) at 40 °C, with an upstream inline filter (3 µm, 1290 infinity II inline filter, Agilent) and SecurityGuard Ultra C18 cartridge as precolumn (2.1 × 2 mm). The oxylipins were separated as described by [21–23] with a gradient composed of 0.1% acetic acid mixed with 5% mobile phase B (mobile phase A) and acetonitrile/methanol/acetic acid (800/150/1, *v/v/v*; mobile phase B) at a flow rate of 0.3 mL min^−1^: 21% B at 0 min, 21% B at 1.0 min, 26% B at 1.5 min, 51% B at 10 min, 66% B at 19 min, 98% B at 25.1 min, 98% B at 27.6 min, 21% B at 27.7 min, and 21% B at 31.5 min. The LC used for oxylipin analysis was coupled with a 5500 QTRAP mass spectrometer operated in negative electrospray ionization (ESI(-)) mode (Sciex, Darmstadt, Germany). The MS was set as follows: ion spray voltage, − 4500 V; capillary temperature, 650 °C; curtain gas N_2_, 50 psi; nebulizer gas (GS1) N_2_, 30 psi; drying gas (GS2) N_2_, 70 psi; generated with N_2_ generator NGM 33 (cmc Instruments, Eschborn, Germany); and collisionally activated dissociation (CAD) gas, high. Declustering potentials (DP), entrance potentials (EP), collision cell exit potentials (CXP), and collision energies (CE) were optimized for each of the oxylipins. MS parameters for oxylipin analysis can be found in ESM Table S14 together with a detailed description of the standard series preparation (ESM Sect. 1). The oxylipin concentrations were quantified using external calibrations with IS, and they were normalized to the absolute protein content determined with bicinchoninic acid assay [27].

The peptides were chromatographically separated with a gradient composed of 95/5% water/acetonitrile (mobile phase A) and 5/95% water/acetonitrile (mobile phase B), both containing 0.1% acetic acid at a flow rate of 0.3 mL min^−1^ as follows: 0% B at 0 min, 0% B at 1 min, 35% B at 30.5 min, 100% B at 30.6 min, 100% B at 33.5 min, 0% B at 33.7 min, and 0% B at 36 min. The LC system for peptide analysis was coupled to a 6500 + hybrid triple quadrupole linear ion trap mass spectrometer (QTRAP; Sciex) in ESI(+)-mode, with the following settings: ion spray voltage, 5500 V; capillary temperature, 550 °C; curtain gas N_2_, 50 psi; nebulizer gas (GS1) N_2_, 60 psi; and drying gas (GS2) N_2_, 60 psi, generated with N_2_ generator Eco Inert-ESP (DTW, Bottrop, Germany). DP, EP, and CXP were set to 40 V, 10 V, and 10 V, respectively, and CE were optimized for each of the peptides (Tables 1 and 2; ESM Table S7). CAD gas was set to medium. Analyst (Sciex, version 1.7) was used for instrument control and data acquisition, and Multiquant (Sciex, version 3.0.2) software was used for data analysis. The peptide/protein concentrations were quantified using external calibrations with IS (ESM Sect. 2.1; ESM Table S5; Tables 1 and 2; and ESM Table S7), and they were normalized to the absolute protein content determined with bicinchoninic acid assay [27].

## Results

The ARA cascade plays a key role in the regulation of many different physiological processes. In order to understand the crosstalk between the different enzymatic pathways of the ARA cascade (Fig. 1) and modulation thereof, quantitative information for both oxylipin levels as well as enzyme/protein abundance is needed.

For this reason, we developed an analytical approach allowing to quantify the enzymes of the ARA cascade and combined it with our targeted oxylipin metabolomics method [21–23]. Combining targeted LC–MS/MS-based proteomics and oxylipin metabolomics as multi-omics methodology allows to quantify the abundance of all relevant enzymes of the COX and the LOX pathways (COX-1 and COX-2, 5-LOX, 12-LOX, 15-LOX, 15-LOX-2, and FLAP) as well as four housekeeping proteins and oxylipin levels from a single sample down to pM ranges.

Oxylipins were extracted from the methanolic supernatant resulting after sonication and precipitation of the cell samples, and enzyme/protein levels were quantified in the precipitated protein residue. Thus, only a *single* sample is required for quantitatively assessing the ARA cascade on metabolite and protein abundance levels in biological samples.

### Targeted proteomics LC–MS/MS/(MS) method

The enzyme abundance is measured in form of representative peptides with amino acid (aa) sequences specific to the target enzyme. Based on an in silico tryptic digestion of the COX and LOX enzymes, two proteotypic peptides with unique [28, 29] aa sequences were selected per enzyme from the multitude of theoretically possible peptides (ESM Table S6). The results from the in silico digestion were narrowed down by a defined set of criteria [18] including fixed peptide lengths (7–22 aa) as well as acceptable calculated cleavage probabilities [30] (e.g., ≥ 70% using cleavage prediction with decision trees [31]) and predicted retention times (3–30 min) [32]. Possible variations in relevant splice variants [33] were considered as well as the presence of maximum two unfavored aa (C, M, N, Q, W). Peptides containing single nucleotide polymorphisms [33] or posttranslational modifications were excluded [33, 34]. After the in silico peptide selection and evaluation of three to five candidates in digested cell matrix, the MS/MS parameters were optimized, and two peptides per protein were finally selected based on their MS sensitivity, selectivity, and chromatographic behavior (Tables 1 and 2; ESM Table S7).

In MS^3^ mode, the triple quadrupole QTRAP instrument uses the linear ion trap (LIT) in Q3 for a second fragmentation of the CAD fragment ions. With the aim of achieving higher selectivity and, thus, sensitivity for quantification of the peptides in complex biological matrices by this additional fragmentation, we chose an MS^3^ approach for the targeted proteomics method. For each peptide, the CE of multiple CAD fragment ions was optimized, and two to three of the most intense fragment ions, ideally with m/z exceeding the precursor ion m/z (e.g., a transition from a double charge precursor to a single charged fragment), were chosen for further evaluation in MS^3^ mode. Their excitation energies (AF2) were optimized in 0.01 V steps, and the final CAD fragment ions for the MS^3^ method were selected based on the highest sensitivities and/or lack of matrix interference in digested cell lysates for each peptide (Table 2).

**Fig. 2:**
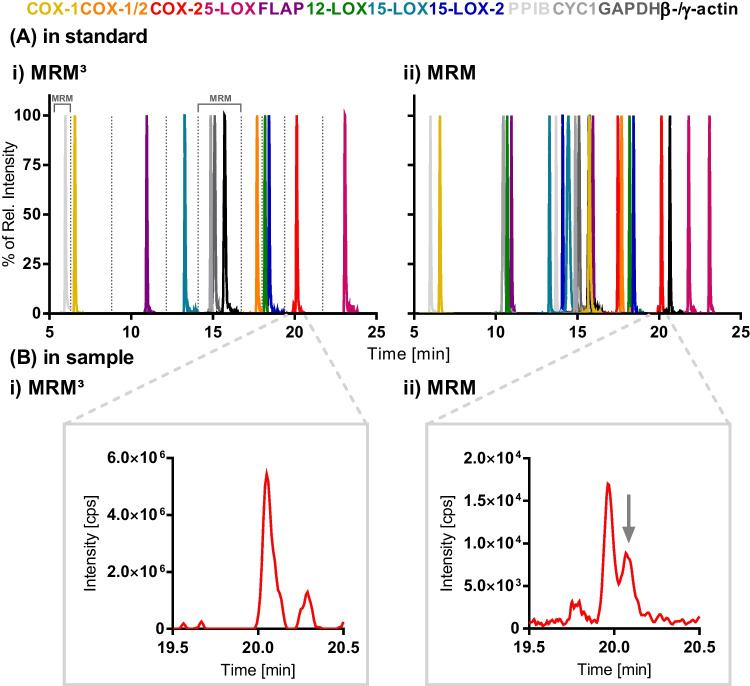
Chromatographic separation of the peptides from the COX and LOX enzyme pathways as well as housekeeping peptides with detection in (i) MRM^3^ and (ii) MRM mode on an LC–MS/MS QTRAP system. Shown are **A** (i) and (ii) a mix of peptide standards (25–100 nM) as well as **B** (i) and (ii) the signal of COX-2 peptide FDPELLFNK in THP-1 cells (i) MS^3^: M^2+^  → y_7_^++^  → Ʃ10 MS^3^ fragments; (ii) MRM: M^2+^  → y_7_^++^. The cells were differentiated for 72 h with vitamin D_3_ (50 nM) and TGF-β1 (1 ng mL^−1^) and treated with LPS (1 μg mL^−1^) for 6 h

The fixed fill time (FFT) for the LIT had a major impact on the signal intensity which increased with longer FFTs (ESM Fig. S1A). The maximum FFT of 250 ms provided the highest sensitivities and was thus used for all peptides (except abundant TGTLAFER, 100 ms, and IS peptides, 25 ms). In order to allow the simultaneous analysis of all peptides with acceptable cycle times and, thus, data points per peak, the analytical run was split into 10 periods (i.e., time windows) with separate MS experiments. Despite excellent chromatographic separation (Table 2; Fig. 2A (i)), with average peak widths at half maximum height (FWHM) of 4.9 s, the number of initially selected peptides needed to be reduced to one peptide per protein for the MRM^3^ method. The selection was made based on the peptides’ sensitivities and retention times to assure that all proteins are detected in the separate time windows of the chromatogram. At a LIT scan rate of 10000 Da s^−1^, a total cycle time of 372–572 ms for each of the eight MS^3^ experiments resulted and thus 9–12 data points over the FWHM of the peak. The peptides of four housekeeping proteins were measured in two periods set in MRM mode with resulting cycle times of 150 and 450 ms at constant dwell times of 20 ms.

For data evaluation, MRM^3^ transitions were constructed from the MS^3^ spectra by the Multiquant 3.0.2 software. Assessing the MRM^3^ transitions of one MS^3^ fragment ion compared to the sum of multiple MS^3^ fragment ions showed higher signal intensity for the use of multiple fragment ions (ESM Fig. S2). Thus, for the final method, the ten most abundant MS^3^ fragment ions of the analyte peptides and five of the IS peptides were selected for data analysis.

The MS^3^ approach was compared to scheduled MRM detection. Here, the windows were set to ± 45 s at the expected retention time and a cycle time of 0.4 s resulting in comparable average 14 data points over FWHM of the chromatographic peaks. Two peptides per protein were included in the method comprising again all COX and relevant LOX pathway enzymes as well as four housekeeping proteins, resulting in a total of 23 peptides (Fig. 2A (ii), Table 1, ESM Table S7). The parallel measurement of three transitions per peptide ensures its identity by calculating the area ratios between one quantifier and two qualifier transitions and comparing the area ratios of the samples to the standards. As acceptance criteria, the ratios for a peak in a biological sample need to be within ± 20% of the area ratio measured in standards (ESM Table S8) [18].

The additional fragmentation in MS^3^ increased selectivity allowing separation of the analyte from interfering matrix signals. This is shown in Fig. 2B (i) and (ii) for the low abundant COX-2 peptide FDPELLFNK in differentiated (50 nM VD_3_ and 1 ng mL^−1^ TGF-β1, 72 h) and LPS-stimulated (1 μg mL^−1^, 6 h) THP-1 cells. The MRM^3^ method enables sensitive detection and quantification of COX and LOX peptides in the medium to high pM range (31–560 pM) (ESM Fig. S3; Table 2). However, the MRM method was more sensitive with up to tenfold lower limits of detection (LOD) ranging from 4.2 to 56 pM and lower limits of quantification (LLOQ) in the range of 16–122 pM for the same peptides (ESM Fig. S3; Table 1). Overfilling of the trap at higher concentrations results in a breakdown of the MS signal (ESM Fig. S1B) and restricts the calibration range of the MRM^3^ method to 4.0–368 nM depending on the peptide (Table 2). This limits the linear working range of the MRM^3^ method to only two to three orders of magnitude. Here, the MRM method also shows a clear advantage allowing linear calibration over approximately five orders of magnitude from the pM LLOQ up to the low μM range (Table 1). Thus, MRM is generally advantageous. If the analyte signal is interfered in matrix, MRM^3^ provides an additional level of selectivity and is useful for complicated biological matrices, while MRM is more sensitive and allows analysis within a large linear range. The developed method is not only sensitive but shows good precision and accuracy as demonstrated for the repeated independent analysis of THP-1 macrophages. The intraday precision was generally ≤ 15%, and interday precision was < 30% in the LPS-stimulated cells (ESM Table S9). The accuracy, determined after spiking the unstimulated cells with peptides during sample preparation, was between 95 and 140% (ESM Table S10). The dual approach of targeted oxylipin metabolomics and proteomics allows the analysis of oxylipin concentrations and protein levels in *one* sample. This powerful tool was applied to comprehensively analyze the ARA cascade in immune cells.

### Analysis of the ARA cascade in immune cells

**Fig. 3:**
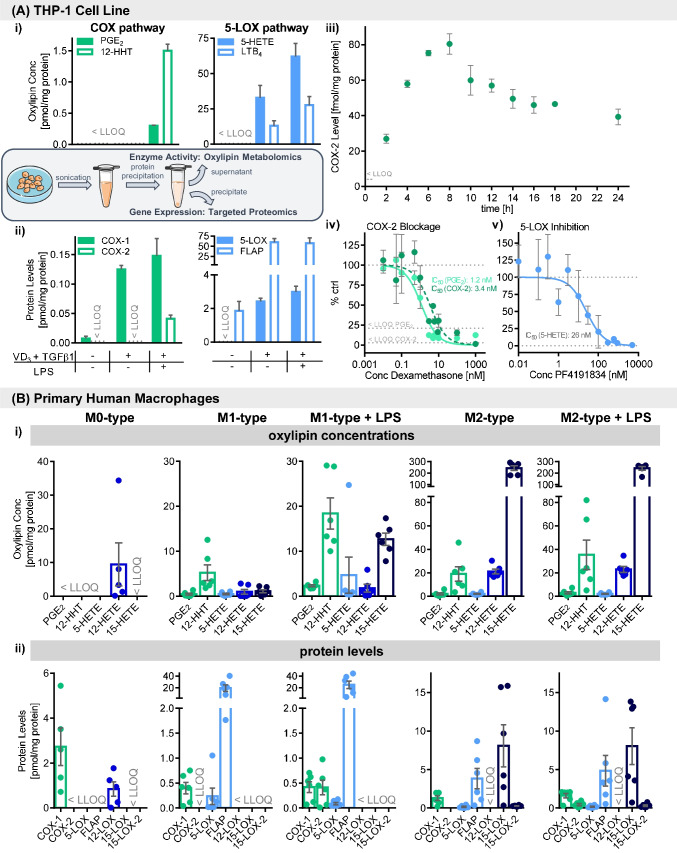
Comprehensive characterization of immune cells using combined targeted oxylipin metabolomics and proteomics: **A** THP-1 cell line and **B** primary human macrophages. **A** (i) Oxylipin concentrations and (ii) enzyme levels in monocytic and macrophage-like THP-1 cell line with and without lipopolysaccharide (LPS) stimulation. Cells were differentiated to macrophages with 50 nM 1,25-dihydroxyvitamin D_3_ (VD_3_) and 1 ng mL^−1^ TGF-β1 for 72 h, with or without LPS stimulation (1 μg mL^−1^) for 6 h (mean ± SD, *n* = 3). **A** (iii) COX-2 abundance following time-dependent LPS stimulation (1 μg mL^−1^). Shown are mean ± SD, *n* = 3. The potencies (IC_50_) of COX-2 and 5-LOX inhibition by **A** (iv) dexamethasone, calculated based on PGE_2_ formation and COX-2 abundance, and **A** (v) 5-LOX inhibitor PF4191834, calculated based on 5-HETE formation, relative to control incubations (0.1% DMSO). Shown are mean ± SD, *n* = 3 – 6. Correlation of **B** (i) oxylipin formation and (ii) enzyme levels in human macrophages derived from primary blood monocytic cells. Cells were differentiated with 10 ng mL^−1^ CSF-2 (M1-like cells) or CSF-1 (M2-like cells) for 8 days. For the final 48 h, they were treated with 10 ng mL^−1^ IFNγ (M1-like cells) or IL-4 (M2-like cells) and with or without 1 μg mL^−1^ LPS for the final 6 h. For M0-like cells, the adhered monocytes were left untreated for 7 days. Shown are mean ± SEM, *n* = 5 – 6

The lipid mediators formed in the ARA cascade are an essential part of the immune system and function i.a. as signaling molecules between different types of immune cells in the host defense. Using the developed LC–MS/MS-based proteomics platform together with the targeted oxylipin metabolomics method, the ARA cascade was comprehensively analyzed in human macrophages for the first time with this novel approach. The monocytes from the THP-1 cell line were examined during differentiation to macrophage-like cells with 50 nM VD_3_ and 1 ng mL^−1^ TGF-β1 for 72 h. This process induced the *ALOX5* gene expression along with 5-LOX product formation (5-HETE and LTB_4_) (Fig. 3A (i), (ii)). While other LOX were not present, COX-1 and FLAP levels increased by 17- and 32-fold, respectively, after differentiation. Additional treatment of the macrophages with 1 μg mL^−1^ LPS for 6 h stimulated *PTGS2* gene expression and formation of PGE_2_ and 12-HHT which was below the detection limit in THP-1 cells bearing COX-1 alone (THP-1 monocytes and macrophages) (Fig. 3A (i), (ii)). The COX-2 protein level increased strongly after LPS (1 μg mL^−1^) treatment from below the detection limit (t_0_) to approximately 80 fmol mg^−1^ protein at the peak after 6–8 h where it declined to 40 fmol mg^−1^ protein after 24 h (Fig. 3A (iii)). Pretreatment of the THP-1 macrophages with dexamethasone suppressed the induction of COX-2 and concomitant prostanoid synthesis with potencies (IC_50_) of 3.4 nM (COX-2; 95% CI, 2.3–4.9 nM) and 1.2 nM (PGE_2_; 95% CI, 0.9–1.6 nM), respectively (Fig. 3A (iv)). The 5-LOX inhibitor PF4191834 suppressed 5-HETE formation with a potency (IC_50_) of 26 nM (95% CI, 12–53 nM) and did not affect the 5-LOX abundance (Fig. 3A (v)).

In the next step, we investigated the expression of ARA cascade genes and oxylipin formation in differently polarized primary human macrophages. The different types of polarization led to distinct oxylipin and protein patterns (Fig. 3B (i), (ii)). In M0-like macrophages, which were derived from primary monocytic cells and incubated without cytokines for 8 days, only COX-1 and 12-LOX as well as its product 12-HETE were detected. However, the presence of both enzymes is most likely attributed to platelet contamination which can be detected with our method since they are highly abundant in these cells (ESM Table S11). Relevant amounts of COX-1, 5-LOX, and FLAP (0.4 ± 0.1, 0.4 ± 0.2, and 19 ± 6 pmol mg^−1^ protein, respectively) were found in the macrophages polarized towards M1-like cells (10 ng mL^−1^ CSF-2 and 10 ng mL^−1^ IFNγ) with the targeted proteomics method. Oxylipins formed via these pathways (PGE_2_, 12-HHT, and 5-HETE) as well as 12- and 15-HETE were detected at low levels (≤ 5 pmol mg^−1^ protein) in the cells (Fig. 3B (i), (ii); ESM Table S12). Stimulation with 1 μg mL^−1^ LPS led to strong elevation of oxylipin concentrations, e.g., fourfold increase of PGE_2_ and 12-HHT as well as an approximately tenfold increase of 5- and 15-HETE. *PTGS2* gene expression was induced by LPS, while the protein levels of COX-1 and FLAP were not modulated, and 5-LOX was slightly reduced. LC–MS analysis of the M2-like macrophages showed an extensive protein pattern: COX-1, 5-LOX, and FLAP as well as 15-LOX and 15-LOX-2 were present. High levels of 15-HETE (243 ± 20 pmol mg^−1^ protein) as well as moderate levels of 12-HETE (21 ± 2 pmol mg^−1^) and 12-HHT (19 ± 6 pmol mg^−1^ protein) dominated the oxylipin profile, while PGE_2_ and 5-HETE were found at approximately 2 pmol mg^−1^ protein (Fig. 3B (i), (ii); ESM Table S12). Interestingly, the additional LPS treatment only led to an approximately twofold increase of PGE_2_ and 12-HHT concentrations but did not affect any of the oxylipins from the LOX pathways. Apart from COX-2 induction, the levels of the ARA cascade enzymes were not changed by LPS (Fig. 3 B (i), (ii)). While the COX-2 levels were similar in both (LPS-stimulated) M1- and M2-like cells, 5-LOX and FLAP levels were two- and fivefold higher in M1-like and COX-1 levels were higher in M2-like macrophages. However, all of the analyzed oxylipins were higher concentrated in M2-like macrophages with the most pronounced differences between M1- and M2-like cells found for 15-HETE (> 200-fold) and 12-HETE (approximately 20-fold) followed by PGE_2_, 12-HHT, and 5-HETE (all approximately fourfold). Regarding the housekeeping proteins, only GAPDH showed strong differences between the M1- and M2-like macrophages indicating that it is not suited for normalization when investigating macrophage polarization (ESM Table S12).

**Fig. 4:**
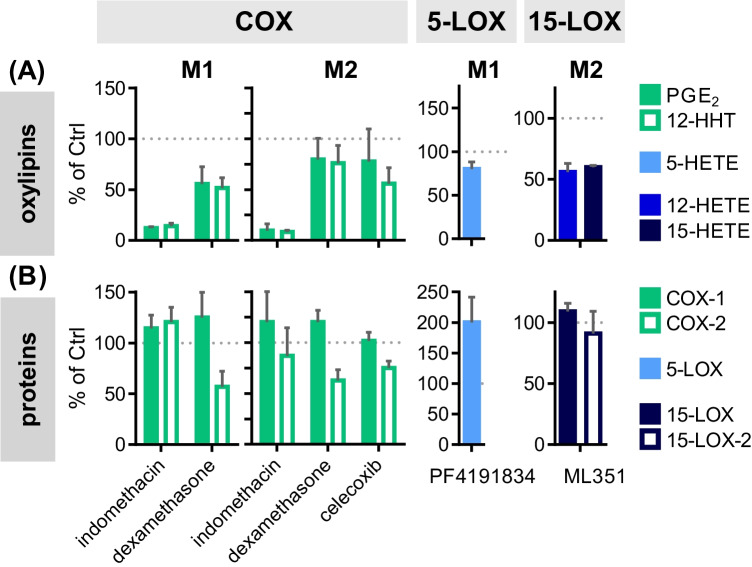
Investigation of ARA cascade modulation in human macrophages using LC–MS/MS-based targeted **A** oxylipin metabolomics and **B** proteomics. Primary blood monocytic cells were differentiated to macrophages with 10 ng mL^−1^ CSF-2 (M1-like cells) or CSF-1 (M2-like cells) for 8 days and with 10 ng mL^−1^ IFNγ (M1-like cells) or IL-4 (M2-like cells) for the final 48 h. The cells were incubated with the different drugs at the following concentrations for the final 7 h during additional LPS stimulation (1 μg mL^−1^) for the final 6 h, 1 μM COX-1/2 inhibitor indomethacin, 100 nM dexamethasone, 5 μM COX-2 inhibitor celecoxib, 5 μM 5-LOX inhibitor PF4191834, 10 μM 15-LOX inhibitor ML351, or 0.1% DMSO as vehicle control. Relative product formation was calculated based on the mean of 2 controls per donor. Shown are mean ± SEM, *n* = 3–5 donors

The ARA cascade is an important target of pharmaceuticals because of its pivotal role in the regulation of the immune response and inflammation. We applied the multi-omics LC–MS/MS-based approach on the quantitative characterization of pharmaceutical modulation of the ARA cascade to demonstrate its usefulness in drug development (Fig. 4A, B; ESM Table S13).

For the experiments, the primary human macrophages polarized towards M1- or M2-like phenotype were pre-incubated with the test compounds at sub-cytotoxic levels (ESM Figs. S4 and S5) for 1 h before LPS was added for the remaining 6 h. The COX-1/COX-2 inhibitor indomethacin strongly reduced the PGE_2_ and 12-HHT concentrations in both M1- and M2-like macrophages without relevantly modulating the COX-1 or COX-2 levels. Dexamethasone treatment also led to lowered concentrations of PGE_2_ and 12-HHT with a more pronounced effect in M1 (approximately 50% inhibition) compared to M2-like cells (approximately 20% inhibition). The decrease of prostanoid concentrations occurred together with a decrease of the COX-2 levels which was similar in both types (approximately 40% inhibition) and did not affect COX-1. Both indomethacin and dexamethasone also markedly reduced 15-HETE formation in M1-like macrophages but had no effect in the M2-like cells. The celecoxib treatment of M2-like macrophages led to a moderate inhibition of the PGE_2_ and 12-HHT formation, while the concentrations of LOX products slightly increased. COX-2 and 15-LOX-2 levels were slightly reduced, and the selective COX-2 inhibitor did not affect COX-1 (Fig. 4A, B; ESM Table S13). The 5-LOX inhibitor PF4191834 hardly reduced the 5-HETE concentration in the M1-like macrophages. The PGE_2_ and 12-HHT concentrations were unaffected by PF4191834, while the 12- and 15-HETE concentrations were slightly reduced. Regarding the 15-LOX pathway, ML351 led to a marked inhibition of both 12- and 15-HETE formation without affecting 15-LOX and 15-LOX-2 levels. 5-LOX abundance was strongly reduced (23 ± 4% of control) with only a slight effect on the 5-HETE concentration. In these incubations, the PGE_2_ and 12-HHT concentrations were moderately increased, and the COX-1 and COX-2 levels were slightly elevated (Fig. 4A, B; ESM Table S13).

Conclusively, we combined our existing targeted oxylipin metabolomics method with an LC–MS/MS-based targeted proteomics method comprising all COX and relevant LOX pathway enzymes as well as four housekeeping proteins. While the more selective detection can be achieved with the MRM^3^ detection method, the MRM approach is characterized by higher sensitivity (in low pM range) and greater linear range up to μM concentrations. With our sensitive multi-omics approach, we were able to determine the oxylipin and protein levels of immune cells in a single sample. We successfully used this approach to thoroughly characterize the ARA cascade in different immune cells and demonstrated that quantitative changes induced by pharmaceutical modulation can be determined on protein and metabolite levels.

## Discussion

Oxylipins formed in the ARA cascade act as potent lipid mediators regulating many physiological functions. In order to profoundly evaluate and understand modulation of this important signaling pathway, it is crucial to investigate not only changes in metabolite concentrations, i.e., eicosanoids and oxylipins, but also on enzyme levels in parallel. Therefore, we combined our targeted oxylipin metabolomics method covering 239 analytes (ESM Table S4) — allowing the quantitative characterization of the complex crosstalk between the different branches of the ARA cascade — with a novel LC–MS/MS-based targeted proteomics approach. The developed targeted proteomics method allows the quantitative analysis of all COX (COX-1 and COX-2) as well as relevant enzymes of the LOX pathway (5-LOX, 12-LOX, 15-LOX, 15-LOX-2, and FLAP) and four housekeeping proteins (β-/γ-actin, PPIB, GAPDH, CYC1). This is the first LC–MS/MS(/MS)-based method for the targeted analysis of the COX and LOX pathways of the ARA cascade.

In targeted proteomics, different MS modes can be used for detection on hybrid triple quadrupole-LIT mass spectrometers. In MRM mode, the analytes are quantified via the pair of a precursor and a specific fragment ion resulting from CAD-based fragmentation. In MRM^3^, these CAD ions are again fragmented in the LIT, and an ion chromatogram is reconstructed from the secondary fragment ions [35]. We compared both approaches in detail. The LIT fill time had a strong effect on sensitivity of the MRM^3^ mode. FFT was preferred over dynamic fill time (DFT) due to its better signal reproducibility and accuracy based on the resulting identical cycle times for every sample [36]. The signal intensity increased with longer FFT (ESM Fig. S1A) in line with literature [36, 37]. Long FFTs, however, have the drawback of a more rapid exhaustion of LIT capacity and breakdown of the MS signal (ESM Fig. S1B). This generally limited the upper calibration range of our MRM^3^ method to low (4 nM) or medium (368 nM) nM concentrations (corresponding to 0.28–9.5 μg mL^−1^ enzyme equivalent) (Table 2), comparable to other proteomics applications of MRM^3^ where linearity was reported for concentrations up to 0.5–20 μg mL^−1^ [35, 36, 38]. Using MRM, however, robust quantification is possible over a concentration range of five orders of magnitude up to low μM concentrations (Table 1; ESM Table S7).

Summing the ten most abundant fragment ions from the MS^3^ spectra as “MRM^3^” during data evaluation enhanced sensitivity (ESM Fig. S2). In MRM^3^, the LODs of the COX and LOX peptides were in the low to medium pM range (equivalent to 11–209 pg enzyme on column) and the LLOQs ranged from 75 to 840 pM, corresponding to 5–63 ng mL^−1^ enzyme equivalent (Table 2; ESM Fig. S3). Other groups reported LLOQs in a similar range for MRM^3^-based quantification on comparable instruments; e.g., several proteins were quantified down to concentrations between 10 and 80 ng mL^−1^ in human serum [35], the LLOQs of two inflammation markers were 7.8 and 156 ng mL^−1^ in plasma [38], and aquaprorin-2 water channel protein could be measured at levels down to 0.5 ng mL^−1^ in human urine (corresponding to 5 ng mL^−1^ in the measuring solution) [36]. Here, the LLOQs were two up to tenfold lower in comparison to MRM-based quantification in matrix [35, 36, 38]. MS^3^ leads to lower signal intensities than MRM due to inevitable losses during each fragmentation step. Thus, the sensitivity gain of MRM^3^ strongly depends on the reduction of interfering signals in biological matrices — the increased selectivity compensates the signal intensity loss [39]. The MRM detection of standards was up to tenfold more sensitive compared to MRM^3^ (Table 1, 2; ESM Fig. S3) and provided sufficient sensitivity and selectivity in cell matrix. However, the additional MS^3^ filtering stage proved helpful to separate the COX-2 peptide FDPELLFNK from closely eluting background matrix in THP-1 cells (Fig. 2B (i), (ii)).

A relevant parameter for quantitative analysis is the number of data points per peak which is defined by the instrument cycle time. In order to enable MRM^3^, the MS method was subdivided into ten time periods (Fig. 2A (i); Table 2) in order to keep these within an accepted range of 10–15 data points per peak (FWHM). Summing the excitation time (25 ms for each MS^3^ fragmentation), FFT (250/100 and 25 ms), and individual scan times per peptide (scan ranges 450–700 Da), the cycle times per period in the MRM^3^ method were all below 600 ms, thus, allowing the detection of acceptable 9–12 data points per peak (FWHM). The long cycle times of the LIT have already been addressed as drawback of MRM^3^ methodology drastically limiting the number of concurrently measurable analytes [39, 40] and thus multiplexing capacities. This might be one of the reasons why MRM^3^ has not (yet) been employed for the analysis of (highly) multiplexed methods, e.g., the targeted analysis of pathway proteomes.

In our view, due to these drawbacks, (i) limited linear range, (ii) higher LLOQs, and (iii) limited multiplexing capacities based on the long cycle times and the use of time periods, the MRM^3^ method is not favored for routine analysis of pathway proteomes such as the ARA cascade. However, it serves as complimentary method, in case of heavy matrix background interference disturbing MRM analysis.

Combining this targeted proteomics approach with our oxylipin metabolomics method, we comprehensively characterized the ARA cascade in immune cells for the first time solely by LC–MS/MS in a single sample. This is especially advantageous for experiments with limited biological material such as primary human cells or tissue also known as single-platform multi-omics [41]. Moreover, if applicable, further merging the sample preparation techniques of proteomics and metabolomics also reduces sample preparation time [42].

The analysis of monocytic THP-1 cells showed that differentiation with VD_3_ and TGFβ1 to macrophage-like cells led to the induction of *ALOX5* gene expression together with a drastic increase in levels of oxylipins (Fig. 3A (i), (ii)). VD_3_/TGFβ1-based differentiation and concomitant increase of *ALOX5* gene activity have been described for several myeloid cell lines (HL-60, Mono Mac 6, THP-1) [43–46]. Concomitant upregulation of the FLAP protein or mRNA levels (Fig. 3A (ii)) were also reported during similar treatments in peripheral blood monocytic cells [47] or the monocytic cell line U937 [48].

The LPS treatment induced upregulation of COX-2 abundance together with increased product formation (Fig. 3A (i)–(iii)). With the quantitative multi-omics approach, we could show a dose-dependent inhibition of LPS-induced PGE_2_ formation and *PTGS2* gene expression by dexamethasone for the first time. Both determined IC_50_ were similar (IC_50_ = 1.2 nM and 3.4 nM) (Fig. 3A (iv)). This is consistent with the described mechanism of dexamethasone i.a. preventing the *PTGS2* gene expression by its mRNA destabilization [49] and concomitantly reducing PGE_2_ formation. The remarkable potencies of dexamethasone in THP-1 macrophages were well within the range determined for inhibited PGE_2_ formation (IC_50_ = 1.6 nM; 95% CI, 1.4–1.9 nM) in LPS-stimulated human monocytes [50]. No IC_50_ values have been determined for the inhibition of the *PTGS2* gene expression with the commonly used semi-quantitative western blot method (relevant inhibition detected at 3 nM to 1 μM) [50, 51]; thus, the novel targeted proteomics method offers new opportunities for such detailed characterization. The competitive 5-LOX inhibitor PF4191834 strongly inhibited 5-LOX product formation in differentiated and LPS-treated THP-1 cells without affecting the 5-LOX abundance (IC_50_ (5-HETE) = 26 nM) (Fig. 3A (v)) fivefold more potently than in human whole blood assay (IC_50_ (LTB_4_) = 130 ± 10 nM) [52]. The commonly used iron–ligand inhibitor zileuton as well as the FLAP inhibitor MK886 had only low inhibitory potential in this cell model which might be caused by interferences induced by the VD_3_/TGFβ1 and/or LPS treatment.

The multi-omics approach allows to obtain true *quantitative* information on the oxylipin concentrations and enzyme abundance levels with sensitive LC–MS/MS methods. For the first time, differently polarized primary human macrophages were characterized with this unique approach and displayed distinct oxylipin and protein patterns for each type (Fig. 3B (i), (ii)). In the non-CSF-treated macrophages (M0-like cells), only COX-1, 12-LOX, and its product 12-HETE were found. This pattern strongly resembles that of platelets (ESM Table S11) [53] which often contaminate monocyte preparations [54]. The presence of other enzymes (5-LOX, FLAP, and 15-LOX-2) and oxylipins at very low abundances as previously reported in M0-like macrophages [24] could not be supported. 5-LOX and FLAP were detected in M1- (CSF-2 and IFNγ-treated) and M2-like (CSF-1 and IL-4 treated) macrophages together with the corresponding oxylipins formed via this pathway (Fig. 3B (i), (ii); ESM Table S12). Varying 5-LOX levels between M1- and M2-like macrophages have been described [24, 55, 56] and thus might be donor-dependent. However, the relatively low 5-HETE concentrations in both macrophage types suggest only low 5-LOX activity and the detected 5-HETE levels could also result from autoxidation. Similarly, the data from the multi-omics investigation showing low levels of 12- and 15-HETE in M1-like macrophages could not be associated to LOX enzyme activity, since 12- and 15-LOX as well as 15-LOX-2 were below the detection limits and thus might be also formed autoxidatively (Fig. 3B (i), (ii), ESM Table S12). The correlation between the tenfold increased 15-HETE concentration and LPS-stimulated COX-2 upregulation in our work is consistent with previous studies demonstrating that 15-HETE is a side product of COX(-2) [57, 58]. In the M2-like macrophages, the multi-omics approach showed that high 15-HETE concentrations dominated their lipid mediator profile which coincided with the presence of 15-LOX and 15-LOX-2 in these cells. This is expected because IL-4 is used during differentiation to M2-like macrophages, causing a strong elevation of 15-LOX and 15-LOX-2 abundances [24, 59, 60]. The dual reaction specificity of 15-LOX [61, 62] giving rise to both 15-HETE as well as 12-HETE also explains the formation of the second most abundant oxylipin 12-HETE in M2-like macrophages which was detected in parallel with the targeted oxylipin metabolomics method. Constitutive *PTGS1* gene expression and LPS-induced *PTGS2* expression were measured in both macrophage types. COX-2 abundances in both macrophage types were comparable, but LPS stimulation led to a more pronounced increase in product synthesis (PGE_2_ and 12-HHT) in M1- vs. M2-like macrophages (Fig. 3B (i), (ii); ESM Table S12). Higher PGE_2_ formation in M1-like cells is also in line with previous reports [24, 55].

The dual targeted oxylipin metabolomics and proteomics approach also allows the detailed investigation of quantitative changes induced by pharmaceuticals on both metabolite and enzyme levels of the ARA cascade (Fig. 4; ESM Table S13).

The COX inhibitors hampered the synthesis of PGE_2_ and 12-HHT in M1- and M2-like macrophages. Indomethacin almost completely blocked product formation — inhibiting COX-1 and COX-2 [63] without affecting the enzyme abundance. Dexamethasone and celecoxib showed less inhibitory effects on product formation due to their specificity to only target COX-2 by direct specific inhibition in case of celecoxib [63] or reduction of its expression by the glucocorticoid dexamethasone [49]. The effect of the latter is also reflected in the results of the targeted proteomics analysis: markedly decreased COX-2 protein levels in M1- and M2-like macrophages (Fig. 4B). Interestingly, 15-HETE formation was reduced to a similar extent as the COX pathway products in indomethacin-like or dexamethasone-treated M1-like but not in the M2-like macrophages. This again demonstrated that 15-HETE must be predominately formed as COX product in M1-like macrophages as byproduct to prostaglandin synthesis [57, 58], while 15-HETE is mainly produced in M2-like macrophages by 15-LOX and 15-LOX-2. The finding underlines that the complexity of the ARA cascade can only be addressed with the use of comprehensive methods such as our multi-omics approach. It also showed that the other prominent LOX pathway products were hardly affected by the COX inhibitors, and only celecoxib caused a notable shunt (increased formation) towards the formation of the hydroxy fatty acids (ESM Table S13). The 5-LOX inhibitor PF4191834 hardly inhibited the 5-HETE formation in M1-like macrophages without a substrate shunt towards the other enzymes (Fig. 4A; ESM Table S13) at a concentration 40-fold above the reported IC_50_ in human whole blood [52]. These results from the multi-omics analysis thus indicate that 5-LOX is hardly active in M1-like macrophages and that 5-HETE seems to be predominantly formed by autoxidation. The determined oxylipin pattern in M2-like macrophages again highlighted the dual reaction specificity of the 15-LOX [61, 62] as its inhibitor ML351 reduced both 12- and 15-HETE concentrations to the same extent. It showed only minimal inhibitory activity towards the other ARA cascade enzymes as described by [64] and rather promoted a substrate shunt towards the COX products. The parallel analysis of the cells with the targeted proteomics method supported that the inhibitor acted only on enzyme activity as the 15-LOX level remained unchanged (Fig. 4; ESM Table S13).

With our comprehensive multi-omics approach, we showed clear correlations between the product and enzyme patterns in different human immune cells. Quantitative changes induced by different pharmaceuticals were assessed on both oxylipin and protein levels providing insights into their modes of action on the modulation of the ARA cascade.

## Conclusion

The combination of the developed proteomics method with our targeted oxylipin metabolomics platform as multi-omics approach allows the quantitative investigation of 239 oxylipins and all COX (COX-1 and COX-2), relevant LOX pathway enzymes (5-, 12-, and 15-LOX, 15-LOX-2, and FLAP) from a single sample. MRM-based detection in proteomics is more favorable compared to MRM^3^ for investigation of the ARA cascade in immune cells due to its higher sensitivity, greater linear range, and higher multiplexing capacities. However, in case of matrix interference, MRM^3^ can be helpful. The application of the combined sensitive oxylipin metabolomics and proteomics approach to different human immune cells proved its usefulness in the thorough characterization of the ARA cascade. Here, it allowed the examination of quantitative changes induced by pharmaceuticals on oxylipin and enzyme abundance levels. Thus, this multi-omics strategy is an indispensable tool to study molecular modes of action involved in the modulation of the ARA cascade and can be used in the future for the investigation, e.g., of novel pharmaceuticals or phytochemicals.

## Electronic supplementary material

Below is the link to the electronic supplementary material.

Supplementary file1 (PDF 512 kb)

Supplementary file2 (PDF 522 kb)

## Abbreviations

aa: Amino acid
ALOX5: Gene of the 5-lipoxgenase enzyme (5-LOX)
ARA: Arachidonic acid
CAD: Collisionally activated dissociation
CE: Collision energy
COX: Cyclooxygenase
CSF: Colony-stimulating factors
CXP: Collision cell exit potential
DFT: Dynamic fill time
DP: Declustering potential
EP: Entrance potential
FFT: Fixed fill time
FLAP: Five-lipoxygenase-activating protein
FWHM: Full width at half maximum
GM-CSF: Granulocyte-macrophage colony-stimulating factor
HETE: Hydroxyeicosatetraenoic acid
HHT: Hydroxyheptadecatrienoic acid
IFNγ: Interferon γ
IL-4: Interleukin 4
IS: Internal standard
LC: Liquid chromatography
LIT: Linear ion trap
LLOQ: Lower limit of quantification
LOD: Limit of detection
LOX: Lipoxygenase
LPS: Lipopolysaccharide
LT: Leukotriene
M-CSF: Macrophage colony-stimulating factor
MRM: Multiple reaction monitoring
MRM^3^: Multiple reaction monitoring cubed
MS: Mass spectrometry
PBMC: Peripheral blood monocytic cells
PBS: Phosphate buffered saline
PG: Prostaglandin
PTGS1/2: Genes of the prostaglandin G/H synthase 1/2 enzymes (COX-1 and COX-2)
P/S: Penicillin/streptomycin
TGF-β1: Transforming growth factor-β1
TRIS: Tris(hydroxymethyl)aminomethane
VD_3_: 1,25-Dihydroxyvitamin D_3_
