# Supplementary material for: Development of a quantitative proteomics approach for cyclooxygenases and lipoxygenases in parallel to quantitative oxylipin analysis allowing the comprehensive investigation of the arachidonic acid cascade
Source: Anal Bioanal Chem. 2023 Jan 23;415(5):913–33. doi: 10.1007/s00216-022-04489-3 (PMC9883352; doi:10.1007/s00216-022-04489-3)
Supplement: Supplementary file 1 — Supplementary file1 (PDF 512 kb) [file 216_2022_4489_MOESM1_ESM.pdf]

Development of a quantitative proteomics approach for cyclooxygenases and lipoxygenases in parallel to quantitative oxylipin analysis allowing the comprehensive investigation of the arachidonic acid cascade

Nicole M. Hartung, Malwina Mainka, Rebecca Pfaff, Michael Kuhn, Sebastian Biernacki, Lilli Zinnert, Nils Helge Schebb*

Chair of Food Chemistry, Faculty of Mathematics and Natural Sciences, University of Wuppertal, Gaußstr. 20, 42119 Wuppertal, Germany

*Corresponding author (Tel: +49 202-439-3457; E-mail: nils@schebb-web.de)

Electronic Supplementary Material

Contents

1 Oxylipin analysis 3
1.1 Standard operating procedure for the preparation of master mixes 4
ESM Table S1 Preparation of master mixes and working stocks from single stock standards. 6
1.2 Verification of standard concentrations 9
1.3 Preparation of dilution series for calibration 10
ESM Table S2 Preparation of new calibration series using master mixes. 11
1.4 Preparation of RT mixture 12
ESM Table S3 Analytes in the retention time mix for identification. 12
ESM Table S4 Oxylipins covered by the targeted oxylipin metabolomics method. 13
2 Proteomics analysis 20
2.1 Preparation of the proteomics calibration series 20
ESM Table S5 Correction factors for peptides. 20
ESM Table S6 Proteotypic peptides for targeted proteomics method. 21
ESM Table S7 Parameters for analysis of housekeeper peptides via LC-MS/MS. 22
ESM Table S8 Identification of peptides. 24
ESM Table S9 Precision: Intra- and interday variability 25
ESM Table S10 Accuracy 26
3 Detailed multi-omics data of human primary macrophages and platelets 27
ESM Table S11 Protein levels in human platelets. 27
ESM Table S12 Investigation of the ARA cascade in primary human macrophages. 28
ESM Table S13 Modulation of the ARA cascade in primary human macrophages. 29
4 MRM^3 analysis 33
ESM Fig. S1 Optimization of QTRAP fill time for MS^3 experiments and evaluation of linear range in MS^3. 33
ESM Fig. S2 Improving MRM^3 analysis. 34
ESM Fig. S3 Comparison of MRM and MRM^3 sensitivities. 35
5 Cell viability assays 36
ESM Fig. S4 Resazurin assay. 36
ESM Fig. S5 Lactate dehydrogenase assay. 37
6 References 38

1 Oxylipin analysis

Preparation of calibration series

An oxylipin calibration series was prepared containing 54 analytes which was used in addition to the established calibration series (1). Here, we provide a detailed description of all steps.

Before the preparation started, all reusable glass ware (e.g. volumetric flasks, volumetric pipettes, gastight syringes) was checked for residual interfering compounds by rinsing them with methanol and analyzing the rinsing solution with the targeted oxylipin metabolomics LC-MS/MS method (1-3). Next, the retention times of the new analytes were determined using the established LC gradient. Their MS parameters were optimized using single stocks of 100 nM which were infused into the MS per flow injection mode without analytical column (0.3 mL min^{-1}, 35/65% A/B). The Q1 *m/z* were determined in Q1 scans. The Q3 *m/z* for the MRM method were selected from the recorded fragment ion spectra with CE ramps over a range of 20 V, under consideration of sensitivity and selectivity. DP and CE were then optimized for the selected transitions.

The single stocks of the internal standards were diluted to the anticipated working concentrations in the calibrators (20 nM, approx. equivalent to 20-times LLOQ) and analyzed with the MRM method. At this concentration 7(*S*),8(*R*),17(*S*)-TriHDHA-d5 (RvD1-d_5) was contaminated with the unlabeled analyte at concentrations >LLOQ. Therefore, we reduced the concentration of this IS by four-fold in the IS master mix and thus, no interference was found at the final calibrator concentration (5 nM).

Then, nine stock mixes ("master mixes", ESM Table S1) were prepared avoiding direct light radiation. The analytes assigned to each of these either differed in retention time or *m/z*, enabling an interference-free measurement in single ion monitoring (SIM) mode for every analyte in each master mix according to Hartung et al. (4). In total, two internal standard master

mixes, seven analyte master mixes and at the same time, working solutions (3-5 µM) for each analyte (for later optimization, etc.), were prepared (ESM Table S1):

1.1 Standard operating procedure for the preparation of master mixes

Pre-arrangements

- Get enough ice boxes/cold packs
- Prepare cleaning solvents
- Prepare working stocks
    - Add fresh MeOH to fresh vial (volume in ESM Table S1)
- Get the needed volumetric flasks (VF) and gas tight syringes (e.g. from Hamilton) ready, after they were checked for residues
- Put a bit of fresh MeOH in the clean VF
- Pipette the masters on **ice**
- Only take **5 single stock STD** out of the -80°C freezer at once

Master mix preparation

- *Work in groups of two, all main steps are done by partner A, unless stated otherwise*
- Warm the vial containing the single stock STD in the hand
    - Vortex
    - Draw the STD and set to correct volume with a gas tight syringe
- Show partner B the set volume
- Partner B checks it off the list or notes the actual volume
- Wipe the syringe tip with lint-free wipe (moistened with MeOH)
- Transfer volume to VF
- Give partner B the single stock STD
- Partner B: Prepare working stock
    - Add 1 µL of single stock STD with pipette to prepared vial with MeOH
    - Vortex
    - Store on ice
    - Close the single stock STD vial tightly
- Take next original vial of single stock STD and restart procedure
- Partner B: clean syringes with cleaning solvents

    - 10 x ACN I
    - 10 x ACN II
    - 10 x MeOH I
    - 10 x MeOH II
    - Dry syringe (move piston up and down)
    - Change cleaning solvents after 5 STDs
    - Wipe the syringe tip with lint-free wipe (moistened with MeOH)
- When all STDs are added to masters, warm VF with hand to RT
    - Fill to mark with MeOH
    - Mix master by turning flask upside down
    - Transfer to flasks with screwcaps
    - Store at -80°C

ESM Table S1 Preparation of master mixes and working stocks from single stock standards.

	Cayman Chemical item no.	Pre-cursor FA	Q1 *m/z*	RT [min]	single stock STD conc [µM]	vol [µL]	master mix conc [µM]	total vol [mL]	working stock volumes (STD + MeOH) [µL]
**IS master I**									
15(*S*)-HETE-d_8	334720	ARA	327.2	19.88	304	32.9	5		
20-HETE-d_6	390030	ARA	325.2	17.97	306	32.7	5		
(±)9(10)-DiHOME-d_4	10009993	LA	317.2	14.84	314	31.9	5		
Leukotriene B_4-d_4	320110	ARA	339.2	13.76	734	13.6	5	2	1 + 100
5(*S*),6(*R*),15(*S*)-TriHETE-d_5 (LxA_4-d_5)	10007737	ARA	356.3	10.09	280	35.8	5		
7(*S*),8(*R*),17(*S*)-TriHDHA-d_5 (RvD1-d_5)	11182	DHA	380.3	10.19	262	9.5	1.25		
7(*S*),16(*R*),17(*S*)-TriHDHA-d_5 (RvD2-d_5)	11184	DHA	380.2	9.40	262	38.2	5		
**IS master II**									
15-deoxy-Δ12,14-PGJ_2-d_4	318570	ARA	319.4	17.68	312	250			1 + 100
PGE_2-d_4	314010	ARA	355.2	8.88	2805	50			1 + 600
PGD_2-d_4	312010	ARA	355.2	9.29	281	250	5	10	1 + 100
13,14-dihydro-15-keto-PGE_2-d_4	10010606	ARA	355.4	10.26	281	250			1 + 100
TxB_2-d_4	319030	ARA	373.3	7.66	267	250			1 + 100
**Master I**									
13,14-dihydro-15-keto-PGD_2 *MaxSpec*	10007208	ARA	351.2	11.18	284	176			1 + 100
11-dehydro-2,3-dinor-TxB_2	19510	ARA	339.3	6.89	294	170			1 + 100
2,3-dinor-TxB_2	19050	ARA	341.2	5.68	292	171			1 + 100
PGD_3	12990	EPA	349.3	8.11	285	175			1 + 100
13,14-dihydro-15-keto-tetranor-PGD_2	13100	ARA	297.2	6.56	335	149			1 + 100
15-keto-PGE_1	13680	DGLA	351.3	9.96	500	100			1 + 100
PGD_1	12000	DGLA	353.2	9.36	500	100	10	5	1 + 100
13,14-dihydro-15-keto-PGD_1	10010425	DGLA	353.3	11.68	500	100			1 + 100
11-dehydro-TxB_2	19500	ARA	367	9.02	1357	37			1 + 300
11-dehydro-TxB_3	19995	EPA	365.3	7.73	273	183			1 + 100
TxB_3	19990	EPA	367.2	6.54	271	184			1 + 100
TxB_2 *MaxSpec*	10007237	ARA	369.2	7.68	270	185			1 + 100
TxB_1	10006610	DGLA	371.3	7.37	500	100			1 + 100

ESM Table S1 continued.

	Cayman Chemical item no.	Pre-cursor FA	Q1 *m/z*	RT [min]	single stock STD conc [µM]	single stock STD vol [µL]	master mix conc [µM]	master mix total vol [mL]	working stock volumes (STD + MeOH) [µL]
**Master II**									
LTB$_5$	21110	EPA	333.3	11.95	299	167			
2,3-dinor-TxB$_1$	10006330	DGLA	343	5.17	290	172			
5(*S*),12(*R*),18(*R*)-TriHEPE (RvE1)	10007848	EPA	349.3	6.25	143	351			
5(*S*),6(*R*),15(*S*)-TriHEPE (LxA$_5$)	10011453	EPA	349.1	8.77	285	175	10	5	1 + 100
15-keto-PGF$_{2\alpha}$ *MaxSpec*	10007227	ARA	351.2	9.17	284	176			
5(*S*),6(*S*),15(*S*)-TriHETE (6(*S*)-LxA$_4$)	10049	ARA	351.2	10.51	284	176			
7(*R*),14(*S*)-DiHDHA (Mar 1)	10878	DHA	359.1	13.60	277	180			
4(*S*),11(*R*),17(*S*)-TriHDHA (RvD3)	13834	DHA	375.3	9.18	266	188			
**Master III**									
13,14-dihydro-15-keto-tetranor-PGE$_2$	13101	ARA	297	7.32	335	149			1 + 100
15-keto-PGE$_2$ *MaxSpec*	10007215	ARA	349.2	9.50	285	175			1 + 100
PGD$_2$ *MaxSpec*	10007202	ARA	351.2	9.37	284	176			1 + 100
8-iso-PGE$_2$	14350	ARA	351.4	8.69	1500	33			1 + 300
5(*S*),14(*R*),15(*S*)-TriHEPE (LxB$_4$)	90420	ARA	351.2	9.15	284	176	10	5	1 + 100
8-iso-PGE$_1$	13360	DGLA	353.4	8.84	1500	33			1 + 300
13,14-dihydro-PGE$_1$	13610	DGLA	355.4	9.81	500	100			1 + 100
20-OH-PGE$_2$	14950	ARA	367.2	3.74	1357	37			1 + 300
7(*S*),16(*R*),17(*S*)-TriHDHA (RvD2)	10007279	DHA	375.3	9.45	266	188			1 + 100
1a,1b-dihomo-PGE$_2$	18665	ARA	379.4	11.40	1510	33			1 + 300
**Master IV**									
15-deoxy-Δ12,14-PGJ$_2$ *MaxSpec*	10007235	ARA	315.2	17.73	316	158			1 + 100
20-HEPE	19322	EPA	317.2	16.76	314	159			1 + 100
2,3-dinor-11β-PGF$_{2\alpha}$	16530	ARA	325.3	5.93	306	163			1 + 100
Δ12-PGJ$_2$	18550	ARA	333.3	11.89	2990	17			1 + 600
22-HDHA	19321	DHA	343.2	19.15	290	172	10	5	1 + 100
PGE$_3$	14990	EPA	349.3	7.74	1427	35			1 + 300
11β-PGF$_{2\alpha}$ *MaxSpec*	10007224	ARA	353.3	7.82	282	177			1 + 100
11β-13,14-dihydro-15-keto PGF$_{2\alpha}$	16540	ARA	353.4	9.83	1410	35			1 + 300
13,14-dihydro-15-keto-PGF$_{2\alpha}$	10007226	ARA	353.3	10.28	282	177			1 + 100
13,14-dihydro-PGF$_{2a}$	16660	ARA	355.4	9.53	500	100			1 + 100

ESM Table S1 continued.

	Cayman Chemical item no.	Pre-cursor FA	Q1 *m/z*	RT [min]	single stock STD conc [µM]	single stock STD vol [µL]	master mix conc [µM]	master mix total vol [mL]	working stock volumes (STD + MeOH) [µL]
**Master V**									
13,14-dihydro-15-keto-PGE_2 *MaxSpec*	10007214	ARA	351.2	10.29	284	176	10	5	1 + 100
2,3-dinor-6-keto-$PGF_{1\alpha}$	15120	DGLA	341.1	7.34	500	100			1 + 100
20-OH $PGF_{2\alpha}$	16950	ARA	369.3	3.59	1350	37			1 + 300
PGE_1	13010	DGLA	353.3	9.20	1500	33			1 + 300
13,14-dihydro-15-keto-PGE_1	13650	DGLA	353.3	10.81	500	100			1 + 100
9,10-DiH stearic acid	28612	OL	315.2	17.29	1504	33			1 + 300
PGB_1	11110	DGLA	335.4	12.27	1500	33			1 + 300
7(*S*),14(*S*)-DiHDHA (7-*epi*-Mar1)	13161	DHA	359.1	13.06	277	180			1 + 100
6,15-diketo-13,14-dihydro-$PGF_{1\alpha}$	15270	DGLA	369.3	7.72	2699	19			1 + 600
PGE_2 *MaxSpec*	10007211	ARA	351.2	8.91	284	176			1 + 100
**Master VI**									
7(*S*),8(*R*),17(*S*)-tri-HDHA (RvD1) *MaxSpec*	25905	DHA	375.3	10.24	27	941	10	2.5	1 + 100
5(*S*),18(*R*)-DiHEPE (RvE2)	13827	EPA	333.2	11.27	299	84			
**Master VII**									
5(*S*),15(*S*)-DiHEPE (RvE4)	29590	EPA	333.2	11.85	299	84	10	2.5	1 + 100

ARA: arachidonic acid (20:4 n6)
DGLA: dihomo-gamma-linolenic acid (20:3 n6)
DHA: docosahexaenoic acid (22:6 n3)
EPA: eicosapentaenoic acid (20:5 n3)
LA: linoleic acid (18:2 n6)
OL: oleic acid (18:1 n9)

1.2 Verification of standard concentrations

Only 12 analytes were available as STD with verified concentrations, i.e. MaxSpec standards (Cayman Chemical, Ann Arbor, MI, USA). In order to check the concentrations of the remaining analytes in regular quality, their SIM areas were compared to those of the MaxSpec STD, assuming comparable ionization efficiency for similar chemical structures as described (4). For this, the master mixes were separately diluted to 100 nM and measured as triplicates in SIM mode using their Q1 *m/z* (ESM Table S1). The mean SIM areas of structurally similar analytes were compared (under consideration of the actual volumes used for master preparation) and a correction factor was calculated if the difference between the analyte and the MaxSpec areas exceeded ± 30%. This was the case for 21 analytes.

1.3 Preparation of dilution series for calibration

The calibration series was prepared by serial dilution as follows

- *Work in groups of two, all main steps are done by partner A, unless stated otherwise*
- Get enough ice boxes/cold packs
- Get the needed volumetric flasks (VF) ready after they were checked for residues (see ESM Table S2)
- Add small volume of fresh MeOH in the clean VF
- Add analyte master mixes/higher or concentrated calibrator (ESM Table S2)
    - Warm the flasks containing the analyte master mixes/calibrator in the hand
    - Vortex
    - Draw the volume of the analyte master mixes/calibrator with a volumetric pipette
    - Wipe the tip with lint-free wipe (moistened with MeOH)
    - Transfer volume to VF which is stored on ice and gently shake
    - Put analyte master mixes/calibrator back on ice immediately
- Partner B: Add IS
    - Warm the flasks containing the IS master mixes in the hand
    - Vortex
    - Draw volumes of IS masters with gastight syringes (ESM Table S2)
    - Wipe the tip with lint-free wipe (moistened with MeOH)
    - Transfer volume to VF which is stored on ice and gently shake
- When all STDs are added to the VF, warm VF with hand to RT
    - Fill to mark with MeOH
        - **CAVE:** Calibrator 17: add exact volume of MeOH
    - Mix calibrator by turning flask upside down
- Repeat procedure until 18 calibrators are prepared (ESM Table S2)
- Transfer each calibrator from VF to multiple vials
- Store at -80°C

ESM Table S2 Preparation of new calibration series using master mixes.

calibrator no.	Analyte conc [nM]	final vol [mL]	type of STD	vol STD [mL]	vol IS master [µL] IS Master I	IS Master II	vol MeOH [mL]	IS conc [nM]
**18**	1000	10	*all masters*	7 x 1	40	40	fill to mark	20
**17**	750	6.667	calibrator 18	5	7	7	1.65	20
**16**	500	25	*all masters*	7 x 1.25	100	100	fill to mark	20
**15**	250	20	calibrator 16	10	40	40		20
**14**	100	25	calibrator 16	5	80	80		20
**13**	50	25	calibrator 16	2.5	90	90		20
**12**	25	25	calibrator 15	2.5	90	90		20
**11**	10	25	calibrator 14	2.5	90	90		20
**10**	5	25	calibrator 13	2.5	90	90		20
**9**	2.5	25	calibrator 12	2.5	90	90		20
**8**	1	25	calibrator 11	2.5	90	90		20
**7**	0.75	20	calibrator 11	1.5	74	74		20
**6**	0.5	25	calibrator 10	2.5	90	90		20
**5**	0.25	25	calibrator 9	2.5	90	90		20
**4**	0.1	25	calibrator 8	2.5	90	90		20
**3**	0.05	20	calibrator 6	2	72	72		20
**2**	0.025	20	calibrator 5	2	72	72		20
**1**	0.01	20	calibrator 4	2	72	72		20

1.4 Preparation of RT mixture

Few analytes with interfering MS transitions could not be fully chromatographically separated and were therefore not added to the master mixes. However, their transitions were added to the targeted oxylipin metabolomics method and a mixture of these analytes was prepared (50 nM, ESM Table S3) in order to be able to monitor them in samples. This retention time mixture is regularly measured together with the calibration series.

ESM Table S3 Analytes in the retention time mix for identification.

Analyte	Cayman Item No.	precursor FA	Q1 *m/z*	RT [min]	interfering oxylipin (RT [min])
11β-PGE_2	14510	ARA	351.2	9.11	LxB_4 (9.15)
15-keto-$PGF_{1α}$ *MaxSpec*	25902	DGLA	353.2	9.46	PGD_1 (9.36)
8-iso-15-keto-PGE_2	14390	ARA	349.2	9.47	15-keto-PGE_2 (9.50)
Δ12-PGD_2	12650	ARA	351.2	8.67	8-iso-PGE2 (8.69) + PGE_2 (8.91)
5(*S*),6(*R*),15(*R*)-TriHETE (15(*R*)-LxA_4)	90415	ARA	351.2	10.22	LxA_4 (10.23)
15(*R*)-PGD_2	10118	ARA	351.2	9.45	PGD_2 (9.37)
15(*R*)-PGE_2	14710	ARA	351.2	8.67	PGE_2 (9.01)
15(*R*)-$PGF_{2α}$	16740	ARA	353.2	8.48	$PGF_{2α}$ (8.65)
7(*S*),8(*R*),17(*R*)-TriHDHA (17(*R*)-RvD1)	13060	DHA	375.3	10.35	7(*S*),8(*R*),17(*S*)-TriHDHA (RvD1; 10.24)
4(*S*),11(*R*),17(*R*)-TriHDHA (17(*R*)-RvD3)	9002880	DHA	375.3	9.12	4(*S*),11(*R*),17(*S*)-TriHDHA (RvD3; 9.18)
8-iso-15(*R*)-$PGF_{2α}$	16395	ARA	353.2	8.48	$PGF_{2α}$ (8.65)

ARA: arachidonic acid (20:4 n6)
DGLA: dihomo-gamma-linolenic acid (20:3 n6)
DHA: docosahexaenoic acid (22:6 n3)

The final targeted LC-MS/MS based oxylipin metabolomics method thus allows to quantitatively measure 239 oxylipins (using 29 IS) derived from twelve different polyunsaturated fatty acid precursors formed via the three enzymatic branches of the ARA cascade as well as autoxidation:

ESM Table S4 Oxylipins covered by the targeted oxylipin metabolomics method.

precursor PUFA	PUFA class	oxylipin	sensitivity LLOQ [nM][1)]
**Oleic acid (18:1 *n*-9)**	epoxy-PUFA	9(10)-Ep-stearic acid	0.5
		trans-9(10)-Ep-stearic acid	0.5
	vic dihydroxy-PUFA	erythro-9,10-DiH-stearic acid	0.50
		threo-9,10-DiH-stearic acid	0.50
**Linoleic Acid (LA; 18:2 *n*-6)**	hydroxy-PUFA	9-HODE	0.35
		10-HODE	0.076
		12-HODE	0.05
		13-HODE	0.25
		15-HODE	0.18
	oxo-PUFA	9-oxo-ODE	0.5
		13-oxo-ODE	0.5
	epoxy-PUFA	9(10)-EpOME	0.2
		trans-9(10)-EpOME	0.2
		12(13)-EpOME	0.037
		trans-12(13)-EpOME	0.037
	vic dihydroxy-PUFA	9,10-DiHOME	0.01
		12,13-DiHOME	0.029
	misc	9,10,11-TriHOME	0.1
		9,10,13-TriHOME	0.1
		9,12,13-TriHOME	0.05
		EKODE	[2)]
**alpha-Linolenic Acid (ALA; 18:3 *n*-3)**	hydroxy-PUFA	9-HOTrE	0.25
		13-HOTrE	0.5
	oxo-PUFA	9-oxo-OTrE	0.25
		13-oxo-OTrE	0.1
	epoxy-PUFA	9(10)-EpODE	0.116
		12(13)-EpODE	0.33
		15(16)-EpODE	0.185
		trans-9(10)-EpODE	0.116
		trans-12(13)-EpODE	0.33
		trans-15(16)-EpODE	0.185
	vic dihydroxy-PUFA	9,10-DiHODE	0.025
		12,13-DiHODE	0.25
		15,16-DiHODE	0.45
	misc	9,10,11-TriHODE	0.05
		9,10,13-TriHODE	1
		9,12,13-TriHODE	0.1
**gamma-Linolenic Acid (GLA; 18:3 *n*-6)**	hydroxy-PUFA	13-γ-HOTrE	2.5
**dihomo-gamma-Linolenic Acid (DGLA; 20:3 *n*-6)**	hydroxy-PUFA	8-HETrE	0.5
		12-HETrE	0.25
		15-HETrE	0.1

	multihydroxy-PUFA	LTB_3	0.25
	epoxy-PUFA	14(15)-EpEDE	0.05
	prostanoids	PGB1	0.10
		PGD_1	0.10
		13,14-dihydro-15-keto-PGD_1	0.50
		PGE_1	0.10
		13,14-dihydro PGE_1	0.35
		13,14-dihydro-15-keto-PGE_1	0.50
		15-keto PGE_1	5.00
		$PGF_{1\alpha}$	0.05
		15-keto-$PGF_{1\alpha}$	
		TxB_1	0.80
	isoprostanes	8-*iso*-PGE_1	0.50
		15-F_{1t}-IsoP (8-iso-$PGF_{1\alpha}$)	1
**Mead acid (20:3 *n*-9)**	hydroxy-PUFA	5-HETrE	0.025
**Arachidonic Acid (ARA; 20:4 *n*-6)**	hydroperoxy-PUFA	5-HpETE	2)
		12-HpETE	2)
		15-HpETE	2)
	hydroxy-PUFA	5-HETE	0.035
		8-HETE	0.23
		9-HETE	0.4
		11-HETE	0.044
		12-HETE	0.25
		15-HETE	0.22
		16-HETE	0.25
		17-HETE	0.25
		18-HETE	0.25
		19-HETE	2.5
		20-HETE	0.5
		tetranor-12-HETE	0.05
		12-HHTrE	0.5
	multihydroxy-PUFA	5(*S*),12(*S*)-DiHETE	0.05
		5(*S*),15(*S*)-DiHETE	0.1
		8(*S*),15(*S*)-DiHETE	1.26
		LTB_4	0.1
		6-*trans*-LTB_4	0.25
		6-*trans*-12-*epi*-LTB_4	0.25
		5(*S*),6(*R*)-DiHETE (ARA)	0.039
		5(*S*),6(*S*)-DiHETE (ARA)	0.045
		20-OH-LTB_4	0.05
		20-COOH-LTB_4	0.17
		18-COOH-dinor-LTB_4	1.0
		12-oxo-LTB_4	0.25
		5(*S*),6(*R*),15(*S*)-TriHETE (LxA_4)	0.25
		5(*S*),6(*S*),15(*S*)-TriHETE (6(*S*)-LxA_4)	1.00
		5(*S*),6(*R*),15(*R*)-TriHETE (15(*R*)-LxA4)	2)
		5(*S*),14(*R*),15(*S*)-TriHEPE (LxB_4)	0.75
	oxo-PUFA	5-oxo-ETE	0.75
		12-oxo-ETE	1.0

		15-oxo-ETE	0.1
	epoxy-PUFA	5(6)-EpETrE	2)
		8(9)-EpETrE	0.5
		11(12)-EpETrE	0.1
		14(15)-EpETrE	0.25
		trans-5(6)-EpETrE	0.5
		trans-8(9)-EpETrE	0.5
		trans-11(12)-EpETrE	0.1
		trans-14(15)-EpETrE	0.25
	vic hydroxy-PUFA	5,6-DiHETrE	0.1
		8,9-DiHETrE	0.068
		11,12-DiHETrE	0.064
		14,15-DiHETrE	0.025
	prostanoids	PGB_2	0.05
		PGD_2	1.00
		15(*R*)-PGD_2	2)
		Δ12-PGD_2	2)
		13,14-dihydro-15-keto PGD_2	0.50
		13,14-dihydro-15-keto-tetranor-PGD_2	1.55
		PGE_2	0.50
		15(*R*)-PGE_2	2)
		11β-PGE_2	2)
		20-OH-PGE_2	1.14
		15-keto PGE_2	0.50
		13,14-dihydro-15-keto-PGE_2	25.00
		13,14-dihydro-15-keto-tetranor-PGE_2	0.79
		1a,1b-dihomo PGE_2	0.06
		$PGF_{2α}$	0.5
		15(*R*)-$PGF_{2α}$	2)
		11β-$PGF_{2α}$	0.75
		20-OH $PGF_{2α}$	1.59
		13,14-dihydro-$PGF_{2α}$	10.00
		15-keto $PGF_{2α}$	0.75
		13,14-dihydro-15-keto-$PGF_{2α}$	1.00
		11β-13,14-dihydro-15-keto $PGF_{2α}$	45.62
		2,3-dinor-11β-$PGF_{2α}$	1.31
		6-keto-$PGF_{1α}$	0.96
		2,3-dinor-6-keto $PGF_{1α}$	0.25
		6,15-diketo-13,14-dihydro $PGF_{1α}$	75.82
		PGJ_2	0.027
		Δ12-PGJ_2	1.28
		15-deoxy-$Δ^{12,14}$-PGJ_2	1.00
		TxB_2	0.50
		2,3-dinor-TxB_2	2.50
		2,3-dinor-TxB_1	2.50
		11-dehydro-2,3-dinor-TxB_2	0.76
		11-dehydro-TxB_2	0.31
	isoprostanes	8-*iso*-PGE_2	0.25
		8-*iso*-15-keto PGE_2	2)
		15-F_{2t}-IsoP (8-iso-$PGF_{2α}$)	0.25
		8-*iso*-15(*R*)-$PGF_{2α}$	2)

		5(*R,S*)-5-F_{2c}-IsoP (8,12-*iso*-iPF$_{2\alpha}$-VI)	0.5
		13,14-dihydro-15-oxo-15-F_{2t}-IsoP	0.50
		15-oxo-15-F_{2t}-IsoP	0.5
		2,3-dinor-15-(*R,S*)-15-F_{2t}-IsoP	0.25
		5(*R,S*)-5-F_{2t}-IsoP (5-iPF$_{2\alpha}$-VI)	0.25
	misc	20-COOH-ARA	0.25
		11,12,15-TriHETrE	0.25
**Eicosapentaenoic Acid (EPA; 20:5 *n*-3)**	hydroxy-PUFA	5-HEPE	0.06
		8-HEPE	0.06
		9-HEPE	0.25
		11-HEPE	0.062
		12-HEPE	0.1
		15-HEPE	0.1
		18-HEPE	0.1
		19-HEPE	0.1
		20-HEPE	0.50
	multihydroxy-PUFA	5(*S*),12(*R*),18(*R*)-TriHEPE (RvE1)	0.50
		5,12,12-TriHEPE (*trans*-RvE1)	2)
		5(*S*),18(*R*)-DiHEPE (RvE2)	2)
		17(*R*),18(*R*)-DiHEPE (RvE3)	2)
		17(*R*),18(S)-DiHEPE (18(*S*)-RvE3)	1.26
		5(*S*),15(*S*)-DiHEPE (RvE4)	0.50
		5(*S*),6(*R*),15(*S*)-TriHEPE (LxA_5)	2.50
		LTB_5	0.50
		5,12-diHEPE	2)
		12,18-diHEPE	2)
		5,x,18-triHEPE 1	2)
		5,x,18-triHEPE 2	2)
	epoxy-PUFA	5(6)-EpETE	2)
		8(9)-EpETE	0.75
		11(12)-EpETE	0.25
		14(15)-EpETE	0.25
		17(18)-EpETE	0.75
		trans-5(6)-EpETE	0.75
		trans-8(9)-EpETE	0.75
		trans-11(12)-EpETE	0.25
		trans-14(15)-EpETE	0.25
		trans-17(18)-EpETE	0.75
	vic hydroxy-PUFA	5,6-DiHETE	0.3
		8,9-DiHETE	0.100
		11,12-DiHETE	0.05
		14,15-DiHETE	0.05
		17,18-DiHETE	0.11
	prostanoids	PGB_3	0.75
		PGD_3	0.75
		PGE_3	0.73
		$PGF_{3\alpha}$	1
		Δ^{17}-6-keto-$PGF_{1\alpha}$	0.50

		TxB_3	4.30
		11-dehydro-TxB_3	1.84
	isoprostanes	15-F_{3t}-IsoP (8-iso-$PGF_{3\alpha}$)	2.5
	misc	12-OH-17(18)-EpETE	1.26
**Docosapentaenoic Acid (DPA; 22:5 *n*-3)**	multihydroxy-PUFA	7(*S*),17(*S*)-DiH-n3DPA	0.75
	oxo-PUFA	17-oxo-n3DPA	5
**Docosahexaenoic Acid (DHA; 22:6 n-3)**	hydroxy-PUFA	4-HDHA	0.1
		7-HDHA	0.1
		8-HDHA	0.1
		10-HDHA	0.05
		11-HDHA	0.25
		13-HDHA	0.1
		14-HDHA	0.14
		16-HDHA	0.25
		17-HDHA	0.9
		20-HDHA	0.25
		21-HDHA	0.25
		22-HDHA	1.00
	multihydroxy-PUFA	7(*R*),14(*S*)-DiHDHA (MaR1)	1.00
		7(*S*),14(*S*)-DiHDHA (7-*epi*-MaR1)	0.75
		13(*R*),14(*S*)-diHDHA (MaR2)	0.25
		7(*S*),8(*R*),17(*S*)-TriHDHA (RvD1)	0.10
		7(*S*),8(*R*),17(*R*)-TriHDHA (17(*R*)-RvD1)	2)
		7(*S*),16(*R*),17(*S*)-TriHDHA (RvD2)	1.00
		4(*S*),11(*R*),17(*R*)-TriHDHA (17(*R*)-RvD3)	2)
		4(*S*),11(*R*),17(*S*)-TriHDHA (RvD3)	0.50
		4(*S*),5(*R*),17(*R,S*)-RvD4	0.25
		7(*S*),17(*S*)-DiHDHA (RvD5)	0.25
		10(*S*),17(*S*)-DiHDHA (PDx)	0.39
	oxo-PUFA	4-oxo-DHA	0.25
		17-oxo-DHA	10
	epoxy-PUFA	4(5)-EpDPE	2)
		7(8)-EpDPE	0.65
		10(11)-EpDPE	0.025
		13(14)-EpDPE	0.1
		16(17)-EpDPE	0.25
		19(20)-EpDPE	0.5
		trans-4(5)-EpDPE	0.65
		trans-7(8)-EpDPE	0.65
		trans-10(11)-EpDPE	0.025
		trans-13(14)-EpDPE	0.1
		trans-16(17)-EpDPE	0.25
		trans-19(20)-EpDPE	0.5
	vic dihydroxy-PUFA	4,5-DiHDPE	0.65
		7,8-DiHDPE	0.5

		10,11-DiHDPE	0.1
		13,14-DiHDPE	0.1
		16,17-DiHDPE	0.1
		19,20-DiHDPE	0.5
Adrenic acid (AdA; 22:4 *n*-6)	prostanoids	1a,1b-dihomo-$PGF_{2\alpha}$	0.75

1) lower limit of quantification (LLOQ) set to lowest calibration standards with a signal to noise ratio ≥5 and accuracy ±20%
2) relative quantification, see ESM Table S14 as separate file

Abbreviations:

**AdA**	adrenic acid
**ALA**	alpha-linolenic acid
**ARA**	arachidonic acid
**DGLA**	dihomo-gamma linolenic acid
**DHA**	docosahexaenoic acid
**DiH**	dihydroxy
**DiHDHA**	dihydroxydocosahexaenoic acid
**DiHDPE**	dihydroxydocosapentaenoic acid
**DiHEPE**	dihydroxyeicosapentaenoic acid
**DiHETE**	dihydroxyeicosatetraenoic acid
**DiHETrE**	dihydroxyeicosatrienoic acid
**DiHODE**	dihydroxyoctadecadienoic acid
**DiHOME**	dihydroxyoctadecamonoenoic acid/ dihydroxyoctadecenoic acid
**DPA**	docosapentaenoic acid
**EKODE**	epoxy-keto-octadecadienoic acid
**Ep**	epoxy
**EPA**	eicosapentaenoic acid
**EpDoTrE**	epoxydocosatrienoic acid
**EpDPE**	epoxydocosapentaenoic acid
**EpEDE**	epoxyeicosadienoic acid
**EpETE**	epoxyeicosatetraenoic acid
**EpETrE**	epoxyeicosatrienoic acid
**EpETrE**	epoxyeicosatrienoic acid
**EpODE**	epoxyoctadecadienoic acid
**EpOME**	epoxyoctadecamonoenoic acid/ epoxyoctadecenoic acid
**ETE**	eicosatetraenoic acid
**FA**	fatty acid
**GLA**	gamma-linolenic acid
**HDHA**	hydroxydocosahexaenoic acid
**HEPE**	hydroxyeicosapentaenoic acid
**HETE**	hydroxyeicosatetraenoic acid
**HETrE**	hydroxyeicosatrienoic acid
**HHTrE**	hydroxyheptatrienoic acid
**HOTrE**	hydroxyoctadecatrienoic acid
**HpETE**	hydroperoxyeicosatetraenoic acid
**IsoP/ iP**	isoprostane
**LA**	linoleic acid
**LT**	leukotriene
**Lx**	lipoxin
**MaR**	maresin
**ODE**	octadecadienoic acid
**Oleic**	oleic acid
**OTrE**	octadecatrienoic acid
**P**	protectin
**PG**	prostaglandin
**Rv**	resolvin
**TriHDHA**	trihydroxydocosahexaenoic acid
**TriHEPE**	trihydroxyeicosapentaenoic acid
**TriHETE**	trihydroxyeicosatetraenoic acid
**TriHETrE**	trihydroxyeicosatrienoic acid
**TriHODE**	trihydroxyoctadecadienoic acid
**TriHOME**	trihydroxyoctadecamonoenoic acid/ trihydroxyoctadecenoic acid
**Tx**	thromboxane

2 Proteomics analysis

2.1 Preparation of the proteomics calibration series

For the quantification of protein abundance levels, two calibration series were prepared: for all COX/LOX peptides and for the peptides of the housekeeping proteins (Table 1, Table 2, ESM Table S7). The calibrations were prepared using unlabeled and heavy labeled (lys: uniformly labeled (U)-$^{13}C_6$; U-$^{15}N_2$; arg: U-$^{13}C_6$; U-$^{15}N_4$) peptide standards as internal standards from JPT Peptides (Berlin, Germany). The absolute concentration of selected COX/LOX peptides (DCPTPMGTK, FDPELLFNK, LILIGETIK, DDGLLVWEIAR, TGTLAFER, LWEIIAR, EITEIGLQGAQDR, ELLIVPGQVVDR, VSTGEAFGAGTWDK) in the calibration solution was validated with unlabeled AQUA peptide standards (> 97% purity, 25-30% concentration precision, Thermo Life Technologies GmbH, Darmstadt, Germany). The concentration was corrected in case of deviations > 10% between both standards (ESM Table S5).

ESM Table S5 Correction factors for peptides. The correction factors were calculated between the peptide standards from JPT Peptides and Thermo Life Technologies GmbH (AQUA peptide standards).

Peptide	correction factor
LILIGETIK	0.53
FDPELLFNK	0.84
DCPTPMGTK	0.31
DDGLLVWEAIR	0.49
TGTLAFER	1.47
LWEIIAR	-
EITEIGLQGAQDR	1.13
VSTGEAFGAGTWDK	-
ELLIVPGQVVDR	0.88

- : no correction factor needed

ESM Table S6 Proteotypic peptides for targeted proteomics method. The proteotypic peptides (PTPs) were selected from an *in silico* tryptic digest of 5-LOX, FLAP, 12-LOX, 15-LOX, 15-LOX-2 and CYC1. The peptides were selected based on peptide length (7-22 aa), uniqueness, cleavage probability calculated with peptide cutter (≥ 90%) or cleavage prediction with decision trees (CP-DT; ≥ 70%), occurrence of single nucleotide polymorphisms (SNPs), variation in splice variants or posttranslational modifications (PTMs), as well as unfavored amino acids (C, M, N, Q, W; max. 2) and predicted retention time (RT; 3 – 30 min).

Peptides	Position	[M+H]$^+$	Length [aa]	Uniqueness[a)]	C-terminal cleavage probability[b)] [%]	Overall cleavage probability[c)] [%]	SNPs[d)]	Variation in splice variants[e)]	PTMs[f)]	Unfavored aa	Pred. RT [min][g)]
**5-Lipoxygenase (5-LOX, P09917, gene: ALOX5)**											
DDGLLVWEAIR	473-483	1286.7	11	unique	100%	98%	-	differs in isoform delta-10-13	-	1 × W	23.50
NLEAIVSVIAER	641-652	1313.7	12	unique	100%	97%	-	missing in isoform delta-10-13 & missing in alpha-10	-	1 × N	20.40
**5-Lipoxygenase-activating protein (FLAP, P20292, gene: ALOX5AP)**											
TGTLAFER	45-52	894.0	8	unique	94%	98%	-	-	-	-	10.80
YFVGYLGER	97-105	1103.2	9	unique	100%	97%	-	-	-	-	15.50
**12-Lipoxygenase (12-LOX, P18054, gene: ALOX12)**											
LWEIIAR	467-473	900.1	7	unique	100%	97%	-	-	-	1 × W	16.70
AVLNQFR	622-628	847.0	7	unique	100%	90%	-	-	-	1 × N; 1 × Q	10.40
**15-Lipoxygenase (15-LOX, P16050, gene: ALOX15)**											
EITEIGLQGAQDR	501-513	1429.7	13	unique	100%	97%	-	-	-	2 × Q	12.80
GFPVSLQAR	514-522	974.5	9	unique	100%	96%	-	-	-	1 × Q	12.00
**15-Lipoxygenase-2 (15-LOX-2, O15296, gene: ALOX 15B)**											
VSTGEAFGAGTWDK	7-21	1425.5	14	unique	90%	94%	-	-	-	1 × W	13.40
ELLIVPGQVVDR	418-429	1337.6	12	unique	100%	95%	-	missing in isoform O15296-2 (15-LOX2sv-b) and O15296-4 (15-LOX2sv-a)	-	1 × Q	17.40
**Cytochrome C1 (CYC1, P08574, gene: CYC1)**											
HLVGVCYTEDEAK*	134-146	1520.7	13	unique	82%	92%	-	-	-	1 × C	8.70
DVCTFLR*	269-275	910.4	7	unique	100%	99%	-	-	-	1 × C	13.00

[a)]from BLAST (5) and NeXtprot (6); [b)]calculated from peptide cutter (7); [c)]calculated from CP-DT (8); [d)]SNPs from Uniprot (9); [e)]splice variants from Uniprot (9); [f)]PTMs from Uniprot (9) and Phosphosite Plus (10); [g)]predicted RT from SSRCalc (11)
-: not reported; *: carbamidomethylated cys

ESM Table S7 Parameters for analysis of housekeeper peptides via LC-MS/MS. (A) Unlabeled and **(B)** heavy labeled (lys: U-$^{13}C_6$; U-$^{15}N_2$; arg: U-$^{13}C_6$; U-$^{15}N_4$) peptide data for housekeeper peptides GAPDH, PPIB, β-/γ-actin, CYC1, updated from Hartung et al. (12). For each peptide, different CAD fragment ions used for qualification and quantification (top) with their Q1 and Q3 *m/z* are shown with retention time (RT, mean ± SD, n =12), relative ratios to quantifier transition as well as collision energies (CE). For unlabeled peptides **(A)** linear calibration range is shown for quantifier transitions, as well as the transitions of the corresponding heavy labeled peptides used as internal standards (IS) for the quantification, limits of detection (LOD) and lower limits of quantification (LLOQ). Accuracy of calibrators was within a range of ± 20%. The spiking levels of the heavy labeled peptides (concentrations in vial) are in shown **(B)**.

(A)

Gene / Protein (UniProtKB No.)	Peptide	Transitions	Q1 *m/z*	Q3 *m/z*	RT [min]	Rel. Ratio to quantifier [%]	CE (V)	IS Transitions	Calibration Range [µM]
**ACTB & ACTG1 / β-Actin & γ-Actin (P60709 / P63261)**	**VAPEEHPVLLTEAPLNPK**	$M^{3+} \rightarrow y_5^+$	652.0	568.4			45		
		$M^{3+} \rightarrow y_{16}^{++}$	652.0	892.5	15.7 ± 0.04	86	38	$M^{3+} \rightarrow y_6^+$	0.01 - 10
		$M^{3+} \rightarrow y_8^+$	652.0	869.5		45	42		
	**DLYANTVLSGGTTMYPGIADR**	$M^{3+} \rightarrow y_6^+$	739.0	628.3			47		
		$M^{3+} \rightarrow y_7^+$	739.0	791.4	20.66 ± 0.01	64	40	$M^{3+} \rightarrow y_6^+$	0.01 - 10
		$M^{3+} \rightarrow y_8^+$	739.0	922.5		31	38		
**PPIB / Peptidyl-prolyl cis-trans isomerase B (PPIB; P23284)**	**IGDEDVGR**	$M^{2+} \rightarrow y_7^+$	430.7	747.3			26		
		$M^{2+} \rightarrow y_6^+$	430.7	690.3	5.99 ± 0.01	27	26	$M^{2+} \rightarrow y_7^+$	0.01 - 10
		$M^{2+} \rightarrow y_5^+$	430.7	575.3		19	31		
	**VLEGMEVVR**	$M^{2+} \rightarrow y_7^+$	516.3	819.4			33		
		$M^{2+} \rightarrow y_6^+$	516.3	690.4	13.69 ± 0.02	41	36	$M^{2+} \rightarrow y_7^+$	0.01 - 7.5
		$M^{2+} \rightarrow y_8^+$	516.3	932.5		12	36		
**GAPDH / Glyceraldehyde-3-phosphate dehydrogenase (GAPDH; P04406)**	**VPTANVSVVDLTCR**	$M^{3+} \rightarrow y_5^+$	510.9	664.3			31		
		$M^{3+} \rightarrow y_3^+$	510.9	436.2	15.75 ± 0.02	48	29	$M^{3+} \rightarrow y_5^+$	0.01 - 10
		$M^{3+} \rightarrow y_4^+$	510.9	549.3		50	37		
	**GALQNIIPASTGAAK**	$M^{2+} \rightarrow y_8^+$	706.4	702.4			43		
		$M^{2+} \rightarrow y_9^+$	706.4	815.5	15.10 ± 0.03	38	46	$M^{2+} \rightarrow y_9^+$	0.01 - 10
		$M^{2+} \rightarrow y_{11}^+$	706.4	1042.6		19	43		
**CYC1 / Cytochrome c1 (CYC1; P08574)**	**HLVGVCYTEDEAK**	$M^{3+} \rightarrow y_6^+$	507.6	692.3			22		
		$M^{3+} \rightarrow y_7^+$	507.6	855.4	10.42 ± 0.07	82	16	$M^{3+} \rightarrow y_6^+$	0.01 - 10
		$M^{3+} \rightarrow b_6^+$	507.6	666.3		58	20		
	**DVCTFLR**	$M^{2+} \rightarrow y_5^+$	455.7	696.4			20		
		$M^{2+} \rightarrow y_5^{++}$	455.7	348.7	14.86 ± 0.03	45	18	$M^{2+} \rightarrow y_5^+$	0.01 - 10
		$M^{2+} \rightarrow y_4^+$	455.7	536.3		40	22		

(B)

Gene / Protein (UniProtKB No.)	Peptide	Transitions	Q1 *m/z*	Q3 *m/z*	RT [min]	Rel. Ratio to quantifier [%]	CE (V)	Spiking level in vial [nM]
**ACTB & ACTG1 / β-Actin & γ-Actin (P60709 / P63261)**	**VAPEEHPVLLTEAPLNPK**	$M^{3+} \rightarrow y_6^+$	654.7	647.4			30	
		$M^{3+} \rightarrow y_7^+$	654.7	776.4	15.7 ± 0.04	98	30	100
		$M^{3+} \rightarrow y_2^+$	654.7	252.2		81	45	
	**DLYANTVLSGGTTMYPGIADR**	$M^{3+} \rightarrow y_6^+$	742.4	638.3			30	
		$M^{3+} \rightarrow y_7^+$	742.4	801.4	20.66 ± 0.01	50	28	100
		$M^{3+} \rightarrow y_8^+$	742.4	932.5		20	28	
**PPIB / Peptidyl-prolyl cis-trans isomerase B (PPIB; P23284)**	**IGDEDVGR**	$M^{2+} \rightarrow y_7^+$	435.7	757.3			21	
		$M^{2+} \rightarrow y_6^+$	435.7	700.3	5.99 ± 0.01	31	21	50
		$M^{2+} \rightarrow y_5^+$	435.7	585.3		17	26	
	**VLEGMEVVR**	$M^{2+} \rightarrow y_7^+$	521.3	829.4			23	
		$M^{2+} \rightarrow y_6^+$	521.3	700.4	13.69 ± 0.02	40	26	50
		$M^{2+} \rightarrow y_8^+$	521.3	942.5		13	26	
**GAPDH / Glyceraldehyde-3-phosphate dehydrogenase (GAPDH; P04406)**	**VPTANVSVVDLTCR**	$M^{3+} \rightarrow y_5^+$	514.3	674.3			21	
		$M^{2+} \rightarrow y_5^+$	770.9	674.3	15.75 ± 0.02	5	40	50
		$M^{3+} \rightarrow y_3^+$	514.3	446.2		46	19	
	**GALQNIIPASTGAAK**	$M^{2+} \rightarrow y_9^+$	710.4	823.5			31	
		$M^{2+} \rightarrow y_{11}^+$	710.4	1050.6	15.10 ± 0.03	40	33	50
		$M^{2+} \rightarrow y_{10}^+$	710.4	936.6		22	33	
**CYC1 / Cytochrome c1 (CYC1; P08574)**	**HLVGVCYTEDEAK**	$M^{3+} \rightarrow y_6^+$	510.2	700.3			22	
		$M^{3+} \rightarrow y_7^+$	510.2	863.4	10.42 ± 0.07	80	16	50
		$M^{3+} \rightarrow b_6^+$	510.2	666.3		61	20	
	**DVCTFLR**	$M^{2+} \rightarrow y_5^+$	460.7	706.4			20	
		$M^{2+} \rightarrow y_5^{++}$	460.7	353.7	14.86 ± 0.03	40	18	50
		$M^{2+} \rightarrow y_4^+$	460.7	546.3		36	22	

ESM Table S8 Identification of peptides. Area ratios between quantifier and qualifier transitions in **(A)** unlabeled and **(B)** heavy labeled peptide standards (n =12 – 23) and samples (n = 12 – 29; human macrophages derived from primary blood monocytic cells). Shown are mean ± SD in % of quantifier transition. All data was obtained by LC-MS/MS based targeted proteomics.

		**(A) Unabeled peptides**					**(B) Heavy labeled peptides**				
			**Standards**		**Samples**			**Standards**		**Samples**	
		**Transitions**	**Mean**	**SD**	**Mean**	**SD**	**Transitions**	**Mean**	**SD**	**Mean**	**SD**
**COX-1**	**DCPTPMGTK**	$M^{2+} \rightarrow y_7^+$	100		100		$M^{2+} \rightarrow y_7^+$	100		100	
		$M^{2+} \rightarrow b_2^+$	59	4	57	6	$M^{2+} \rightarrow b_2^+$	59	1	58	4
		$M^{2+} \rightarrow y_5^+$	43	3	42	5	$M^{2+} \rightarrow y_7^{++}$	17	0.4	17	1
	**AEHPTWGDEQLFQTTR**	$M^{3+} \rightarrow y_5^+$	100		100		$M^{3+} \rightarrow y_5^+$	100		100	
		$M^{3+} \rightarrow y_4^+$	57	7	59	6	$M^{3+} \rightarrow y_4^+$	53	1	55	1
		$M^{3+} \rightarrow y_6^+$	55	5	58	9	$M^{3+} \rightarrow y_6^+$	50	2	50	2
**COX-2**	**FDPELLFNK**	$M^{2+} \rightarrow y_7^{++}$	100		100		$M^{2+} \rightarrow y_7^{++}$	100		100	
		$M^{2+} \rightarrow y_7^+$	36	2	34	1	$M^{2+} \rightarrow y_7^+$	34	0.4	33	1
		$M^{2+} \rightarrow b_2^+$	25	2	22	4	$M^{2+} \rightarrow y_4^+$	6	0.1	6	0
	**NAIMSYVLTSR**	$M^{2+} \rightarrow y_8^+$	100		100		$M^{2+} \rightarrow y_8^+$	100		100	
		$M^{2+} \rightarrow b_3^+$	92	23	82	7	$M^{2+} \rightarrow b_3^+$	92	3	84	7
		$M^{2+} \rightarrow y_9^+$	43	6	38	6	$M^{2+} \rightarrow y_7^+$	70	2	69	6
**COX-1/2**	**LILIGETIK**	$M^{2+} \rightarrow y_7^+$	100		100		$M^{2+} \rightarrow y_7^+$	100		100	
		$M^{2+} \rightarrow b_2^+$	62	4	61	5	$M^{2+} \rightarrow y_6^+$	23	2	23	1
		$M^{2+} \rightarrow y_5^+$	30	2	47	22	$M^{2+} \rightarrow y_8^+$	4	0.1	5	0.3
**5-LOX**	**DDGLLVWEAIR**	$M^{2+} \rightarrow y_6^+$	100		100		$M^{2+} \rightarrow y_6^+$	100		100	
		$M^{2+} \rightarrow y_7^+$	81	5	84	8	$M^{2+} \rightarrow y_7^+$	78	2	82	4
		$M^{2+} \rightarrow y_5^+$	85	5	85	7	$M^{2+} \rightarrow y_5^+$	83	2	85	3
	**NLEAIVSVIAER**	$M^{2+} \rightarrow y_7^+$	100		100		$M^{2+} \rightarrow y_6^+$	100		100	
		$M^{2+} \rightarrow y_{10}^+$	66	2	57	7	$M^{2+} \rightarrow y_8^+$	76	1	78	4
		$M^{2+} \rightarrow y_8^+$	43	2	52	6	$M^{2+} \rightarrow y_4^+$	36	1	37	2
**FLAP**	**TGTLAFER**	$M^{2+} \rightarrow y_5^+$	100		100		$M^{2+} \rightarrow y_4^+$	100		100	
		$M^{2+} \rightarrow y_3^+$	70	6	72	4	$M^{2+} \rightarrow y_5^+$	44	1	42	2
		$M^{2+} \rightarrow y_6^+$	55	7	56	4	$M^{2+} \rightarrow y_3^+$	32	0.5	32	1
	**YFVGYLGER**	$M^{2+} \rightarrow y_7^+$	100		100		$M^{2+} \rightarrow y_7^+$	100		100	
		$M^{2+} \rightarrow b_2^+$	67	6	68	7	$M^{2+} \rightarrow b_2^+$	66	1	75	13
		$M^{2+} \rightarrow y_6^+$	69	3	71	7	$M^{2+} \rightarrow y_6^+$	72	1	74	5
**12-LOX**	**LWEIIAR**	$M^{2+} \rightarrow y_5^+$	100		100		$M^{2+} \rightarrow y_6^+$	100		100	
		$M^{2+} \rightarrow b_2^+$	32	2	34	6	$M^{2+} \rightarrow y_4^+$	87	2	90	7
		$M^{2+} \rightarrow y_6^+$	22	2	22	1	$M^{2+} \rightarrow y_3^+$	44	1	43	2
	**AVLNQFR**	$M^{2+} \rightarrow y_5^+$	100		100		$M^{2+} \rightarrow y_5^+$	100		100	
		$M^{2+} \rightarrow y_4^+$	47	3	40	13	$M^{2+} \rightarrow y_3^+$	7	0.2	8	1
		$M^{2+} \rightarrow y_3^+$	6	2	8	1	$M^{2+} \rightarrow z_4^+$	6	0.2	6	0.4
**15-LOX**	**EITEIGLQGAQDR**	$M^{2+} \rightarrow y_8^+$	100		100		$M^{2+} \rightarrow y_8^+$	100		100	
		$M^{2+} \rightarrow y_5^+$	38	1	38	2	$M^{2+} \rightarrow y_5^+$	39	1	38	1
		$M^{2+} \rightarrow y_9^+$	30	2	30	1	$M^{2+} \rightarrow y_9^+$	30	1	31	1
	**GFPVSLQAR**	$M^{2+} \rightarrow y_7^{++}$	100		100		$M^{2+} \rightarrow y_7^{++}$	100		100	
		$M^{2+} \rightarrow y_5^+$	28	1	28	1	$M^{2+} \rightarrow y_5^+$	28	0.4	28	1
		$M^{2+} \rightarrow y_7^+$	18	1	18	1	$M^{2+} \rightarrow y_6^+$	10	0.3	10	0.3
**15-LOX-2**	**ELLIVPGQVVDR**	$M^{2+} \rightarrow y_7^+$	100		100		$M^{2+} \rightarrow y_7^+$	100		100	
		$M^{2+} \rightarrow b_5^+$	32	3	32	5	$M^{2+} \rightarrow y_8^+$	30	0.5	31	1
		$M^{2+} \rightarrow y_8^+$	32	1	32	3	$M^{2+} \rightarrow b_5^+$	30	1	30	1
	**VSTGEAFGAGTWDK**	$M^{2+} \rightarrow y_7^+$	100		100		$M^{2+} \rightarrow y_7^+$	100		100	
		$M^{2+} \rightarrow y_8^+$	83	4	78	9	$M^{2+} \rightarrow y_8^+$	74	2	78	3
		$M^{2+} \rightarrow y_9^+$	79	4	73	12	$M^{2+} \rightarrow y_{12}^{++}$	58	2	58	4

ESM Table S8 continued.

**β-Actin & γ-Actin**	**VAPEEHPVLLTEAPLNPK**	$M^{3+} \rightarrow y_5^+$	100		100		$M^{3+} \rightarrow y_6^+$	100		100	
		$M^{3+} \rightarrow y_{16}^{++}$	86	9	68	14	$M^{3+} \rightarrow y_7^+$	98	4	94	14
		$M^{3+} \rightarrow y_8^+$	45	2	42	3	$M^{3+} \rightarrow y_2^+$	81	5	89	7
	**DLYANTVLSGGTTMYPGIADR**	$M^{3+} \rightarrow y_6^+$	100		100		$M^{3+} \rightarrow y_6^+$	100		100	
		$M^{3+} \rightarrow y_7^+$	64	4	66	2	$M^{3+} \rightarrow y_7^+$	50	3	49	2
		$M^{3+} \rightarrow y_8^+$	31	2	34	3	$M^{3+} \rightarrow y_8^+$	20	1	20	4
**PPIB**	**IGDEDVGR**	$M^{2+} \rightarrow y_7^+$	100		100		$M^{2+} \rightarrow y_7^+$	100		100	
		$M^{2+} \rightarrow y_6^+$	27	1	27	3	$M^{2+} \rightarrow y_6^+$	31	1	30	1
		$M^{2+} \rightarrow y_5^+$	19	1	20	3	$M^{2+} \rightarrow y_5^+$	17	0.4	17	0.5
	**VLEGMEVVR**	$M^{2+} \rightarrow y_7^+$	100		100		$M^{2+} \rightarrow y_7^+$	100		100	
		$M^{2+} \rightarrow y_6^+$	41	1	43	2	$M^{2+} \rightarrow y_6^+$	40	1	41	1
		$M^{2+} \rightarrow y_8^+$	12	0.3	11	1	$M^{2+} \rightarrow y_8^+$	13	0.3	13	0.4
**GAPDH**	**VPTANVSVVDLTCR**	$M^{3+} \rightarrow y_5^+$	100		100		$M^{3+} \rightarrow y_5^+$	100		100	0
		$M^{3+} \rightarrow y_3^+$	48	1	53	4	$M^{2+} \rightarrow y_5^+$	5	2	8	4
		$M^{3+} \rightarrow y_4^+$	50	2	50	3	$M^{3+} \rightarrow y_3^+$	46	2	48	2
	**GALQNIIPASTGAAK**	$M^{2+} \rightarrow y_8^+$	100		100		$M^{2+} \rightarrow y_9^+$	100		100	
		$M^{2+} \rightarrow y_9^+$	38	2	34	4	$M^{2+} \rightarrow y_{11}^+$	40	2	38	2
		$M^{2+} \rightarrow y_{11}^+$	19	1	16	3	$M^{2+} \rightarrow y_{10}^+$	22	1	22	1
**CYC1**	**HLVGVCYTEDEAK**[1]	$M^{3+} \rightarrow y_6^+$	100		100		$M^{3+} \rightarrow y_6^+$	100		100	
		$M^{3+} \rightarrow y_7^+$	82	3	81	27	$M^{3+} \rightarrow y_7^+$	80	3	68	42
		$M^{3+} \rightarrow b_6^+$	58	1	136	142	$M^{3+} \rightarrow b_6^+$	61	1	94	145
	**DVCTFLR**	$M^{2+} \rightarrow y_5^+$	100		100		$M^{2+} \rightarrow y_5^+$	100		100	0
		$M^{2+} \rightarrow y_5^{++}$	45	1	47	2	$M^{2+} \rightarrow y_5^{++}$	40	2	43	2
		$M^{2+} \rightarrow y_4^+$	40	1	40	3	$M^{2+} \rightarrow y_4^+$	36	1	37	1

[1]: interference, not used for quantification

ESM Table S9 Precision: Intra- and interday variability of the targeted proteomics analysis was determined in THP-1 monocytes differentiated to macrophages (50 nM 1,25-dihydroxyvitamin D_3 and 1 ng mL^{-1} TGF-β1 for 72 h, stimulated with 1 µg mL^{-1} LPS for 6 h). Variability was calculated as relative standard deviation of the same sample prepared independently three times on the same day (intraday) and on three different days (interday).

protein	peptide	precision	
		intraday [%]	interday [%]
**COX-1**	DCPTPMGTK	10	24
**COX-2**	FDPELLFNK	6	28
**5-LOX**	DDGLLVWEAIR	10	21
**FLAP**	TGTLAFER	34	42
**ACTB**	VAPEEHPVLLTEAPLNPK	5	15
**PPIB**	IGDEDVGR	13	25
**GAPDH**	GALQNIIPASTGAAK	5	11
**CYC1**	DVCTFLR	8	21

ESM Table S10 Accuracy of the targeted proteomics method. THP-1 monocytes differentiated to macrophages (50 nM 1,25-dihydroxyvitamin D_3 and 1 ng mL^{-1} TGF-β1 for 72 h) were spiked with unlabeled peptides during sample preparation after tryptic digestion. The accuracy was determined as the mean (n = 3) % of the nominal concentration (4 nM FDPELLFNK, 5 nM LWEIIAR, 20 nM EITEIGLQGAQDR, 8 nM ELLIVPGQVVDR).

protein	peptide	accuracy [%]
**COX-2**	FDPELLFNK	140
**12-LOX**	LWEIIAR	131
**15-LOX**	EITEIGLQGAQDR	95
**15-LOX-2**	ELLIVPGQVVDR	122

3 Detailed multi-omics data of human primary macrophages and platelets

ESM Table S11 Protein levels in human platelets. Platelet-rich plasma was generated from EDTA-blood after centrifugation and platelets were then isolated from the platelet-rich plasma after subsequent centrifugation. Protein levels were quantified via LC-MS/MS based targeted proteomics, shown are mean ± SEM in pg mg^{-1} protein from n=3 donors.

<table>
<tr><th></th><th colspan="7">Protein abundance levels [pg mg^{-1}] total protein in human platelets</th></tr>
<tr><th>donor</th><th>COX-1</th><th>COX-2</th><th>5-LOX</th><th>FLAP</th><th>12-LOX</th><th>15-LOX</th><th>15-LOX-2</th></tr>
<tr><td>A</td><td>1.2</td><td rowspan="3"><LLOQ</td><td rowspan="3"><LLOQ</td><td rowspan="3"><LLOQ</td><td>0.7</td><td rowspan="3"><LLOQ</td><td rowspan="3"><LLOQ</td></tr>
<tr><td>B</td><td>1.6</td><td>0.6</td></tr>
<tr><td>C</td><td>0.5</td><td>0.4</td></tr>
</table>

ESM Table S12 Investigation of the ARA cascade in primary human macrophages. (A) Oxylipin concentrations and **(B)** protein levels in human macrophages derived from primary blood monocytic cells. Cells were differentiated with 10 ng mL^{-1} CSF-2 (M1-like cells) or CSF-1 (M2-like cells) for 8 days. For the final 48 h, they were treated with 10 ng mL^{-1} IFNγ (M1-like cells) or IL-4 (M2-like cells) and with or without 1 µg mL^{-1} LPS for the final 6 h. For M0-like cells, the adhered monocytes were left untreated for 8 days (mean ± SEM, n=5-6). All data was obtained by LC-MS/MS based targeted oxylipin metabolomics and proteomics. Peptides highlighted in bold were quantified using AQUA standards (Section 2).

	[pmol/mg protein]	M0	M1	M1 + LPS	M2	M2 + LPS
oxylipin conc.	PGE_2	< LLOQ	0.6 ± 0.2	2.3 ± 0.5	2.0 ± 0.8	3 ± 1
	12-HHT	< LLOQ	5 ± 1	18 ± 5	19 ± 6	35 ± 13
	5-HETE	< LLOQ	0.5 ± 0.1	5 ± 3	2.1 ± 0.4	2.1 ± 0.4
	12-HETE	9 ± 6	1.0 ± 0.5	2 ± 3	21 ± 2	23 ± 3
	15-HETE	< LLOQ	1.1 ± 0.4	13 ± 3	243 ± 20	241 ± 15

	[pmol/mg protein]	Peptide	M0	M1	M1 + LPS	M2	M2 + LPS
protein levels	COX-1	**DCPTPMGTK**	2.7 ± 0.8	0.4 ± 0.1	0.4 ± 0.1	1.3 ± 0.3	1.6 ± 0.3
		AEHPTWGDEQLFQTTR	3 ± 1	0.6 ± 0.1	0.9 ± 0.4	2 ± 1	2.0 ± 0.5
	COX-2	**FDPELLFNK**	< LLOQ	< LLOQ	0.4 ± 0.1	< LLOQ	0.5 ± 0.1
		NAIMSYVLTSR	< LLOQ	< LLOQ	2.7 ± 0.8	< LLOQ	2.2 ± 0.7
		**LILIGETIK**	1.1 ± 0.4	0.20 ± 0.07	1.1 ± 0.4	0.5 ± 0.1	1.7 ± 0.4
	5-LOX	**DDGLLVWEAIR**	< LLOQ	0.4 ± 0.2	0.13 ± 0.02	0.18 ± 0.08	0.3 ± 0.1
		NLEAIVSVIAER	< LLOQ	< LLOQ	< LLOQ	1.3 ± 0.6	1.3 ± 0.6
	FLAP	**TGTLAFER**	< LLOQ	19 ± 6	25 ± 7	3.8 ± 1.3	5 ± 2
		YFVGYLGER	< LLOQ	4 ± 1	5 ± 2	0.8 ± 0.3	1.0 ± 0.3
	12-LOX	**LWEIIAR**	0.8 ± 0.3	< LLOQ	< LLOQ	< LLOQ	< LLOQ
		AVLNQFR	4.4 ± 0.9	< LLOQ	< LLOQ	< LLOQ	< LLOQ
	15-LOX	**EITEIGLQGAQDR**	< LLOQ	< LLOQ	< LLOQ	8 ± 3	8 ± 2
		GFPVSLQAR	< LLOQ	< LLOQ	< LLOQ	22 ± 7	22 ± 7
	15-LOX-2	**ELLIVPGQVVDR**	< LLOQ	< LLOQ	< LLOQ	0.28 ± 0.03	0.3 ± 0.1
		**VSTGEAFGAGTWDK**	< LLOQ	< LLOQ	< LLOQ	0.36 ± 0.04	0.3 ± 0.1
	ACTB	VAPEEHPVLLTEAPLNPK	1825 ± 557	2977 ± 710	3487 ± 804	3164 ± 247	3219 ± 384
		DLYANTVLSGGTTMYPGIADR	21965 ± 8354	32069 ± 8687	38167 ± 10622	38180 ± 3314	38648 ± 5282
	PPIB	IGDEDVGR	3 ± 2	13 ± 4	20 ± 5	17 ± 3	18 ± 3
		VLEGMEVVR	3 ± 1	59 ± 10	72 ± 15	60 ± 5	64 ± 3
	GAPDH	VPTANVSVVDLTCR	78 ± 33	72 ± 43	31 ± 16	264 ± 16	267 ± 31
		GALQNIIPASTGAAK	78 ± 21	82 ± 35	44 ± 20	283 ± 24	278 ± 31
	CYC1	HLVGVCYTEDEAK	10 ± 3	4 ± 1	5 ± 1	3 ± 1	4 ± 1
		DVCTFLR	1.4 ± 0.4	6 ± 1	7 ± 2	5 ± 1	6 ± 1

ESM Table S13 Modulation of the ARA cascade in primary human macrophages. Effects of ARA cascade modulation on **(A)** oxylipin concentrations and **(B)** protein levels of the COX, 5-,12-, 15-LOX and 15-LOX-2 pathways in human macrophages derived from primary blood monocytic cells. Cells were differentiated with 10 ng mL^{-1} CSF-2 (M1-like cells) or CSF-1 (M2-like cells) for 8 days and with 10 ng mL^{-1} IFNγ (M1-like cells) or IL-4 (M2-like cells) for the final 48 h. The cells were incubated with the different pharmaceuticals at the following concentrations for the final 7 h during additional LPS stimulation (1 µg mL^{-1}) for the final 6 h: 1 µM COX-1/2 inhibitor indomethacin, 100 nM dexamethasone, 5 µM COX-2 inhibitor celecoxib, 5 µM 5-LOX inhibitor PF4191834, 10 µM 15-LOX inhibitor ML351 or 0.1% DMSO as control.

The concentrations of **(A) i)** oxylipins and **(B) i)** proteins were determined in each sample and **(A) ii), (B) ii)** calculated relative to the mean of both controls per donor as well as **(A) iii), (B) iii)** the overall means ± SEM per test compound. In case the concentrations of analytes were < LLOQ and ≥ LOD the LOD was used and for concentrations < LOD the half LLOQ was used for relative calculation. All data was obtained by LC-MS/MS based targeted oxylipin metabolomics and proteomics.

			(A) i) Oxylipin conc [pmol mg^{-1} protein]					(B) i) Protein levels [pmol mg^{-1} protein]						
	Donor	Incubation	12-HHT	PGE_2	5-HETE	12-HETE	15-HETE	COX-1	COX-2	5-LOX	FLAP	12-LOX	15-LOX	15-LOX-2
M1 + 1 µg mL^{-1} LPS	A	Ctrl. 1	17	0.61	0.26	0.33	7.4	0.39	0.12	0.15	24			
		Ctrl. 2	18	0.71	0.32	0.21	5.2	0.61	0.17	0.24	41			
		Indomethacin	1.4	0.077	0.26	0.19	0.43	0.62	0.20	0.23	41	< LOD	< LOD	< LOD
		Dexamethasone	16	0.77	0.26	0.26	3.6	0.59	0.077	0.28	39			
		PF4191834	18	0.88	0.25	0.16	2.8	0.65	0.14	0.46	43			
	B	Ctrl. 1	20	1.2	0.49	2.0	18	0.41	0.22	0.086	21			
		Ctrl. 2	20	1.1	0.58	1.7	17	0.48	0.27	0.11	27			
		Indomethacin	4.3	0.18	0.49	1.2	1.5	0.63	0.36	0.15	35	< LOD	< LOD	< LOD
		Dexamethasone	13	0.55	0.54	1.0	9.3	0.87	0.24	0.22	49			
		PF4191834	15	0.89	0.52	0.25	11	0.74	0.29	0.30	38			
	C	Ctrl. 1	30	2.4	0.69	0.26	16	1.2	0.56	0.22	49			
		Ctrl. 2	22	1.9	0.77	0.21	12	0.94	0.41	0.27	47			
		Indomethacin	3.6	0.25	0.71	0.44	0.81	1.1	0.49	0.20	49	< LOD	< LOD	< LOD
		Dexamethasone	16	1.4	1.2	0.20	7.8	0.93	0.17	0.30	46			
		PF4191834	40	2.8	0.44	0.14	11	1.1	0.38	0.31	44			
	D	Ctrl. 1	37	5.4	1.9	0.17	18	1.3	0.75	0.44	41			
		Ctrl. 2	29	4.3	1.7	0.26	18	1.1	0.58	0.35	29			
		Indomethacin	4.3	0.42	2.0	0.35	1.1	1.0	0.60	0.32	24	< LOD	< LOD	< LOD
		Dexamethasone	14	2.1	5.5	0.22	5.7	1.2	0.25	0.64	31			
		PF4191834	32	3.4	1.3	0.34	13	1.1	0.43	0.56	18			
M2 +1 µg mL^{-1} LPS	A	Ctrl .1	38	2.3	0.42	10	114	2.0	0.29	0.13	4.2		17	0.26
		Ctrl. 2	38	2.0	0.47	10	110	2.2	0.31	0.12	4.6	< LOD	18	0.24
		Dexamethasone	25	2.2	0.53	12	125	2.0	0.15	0.14	3.8		18	0.31
	B	Ctrl .1	29	2.8	0.50	11	143	1.4	0.20	0.062	2.7		17	0.17
		Ctrl. 2	39	3.4	0.82	11	154	1.5	0.19	0.10	3.1	< LOD	19	0.18
		ML351	37	3.8	0.63	5.3	94	2.0	0.36	< LOD	3.5		22	0.25
	C	Ctrl .1	27	2.4	1.5	16	56	0.75	0.26	0.064	3.4		0.38	0.079
		Ctrl. 2	29	2.9	1.3	19	65	0.40	0.20	0.044	2.4	< LOD	0.24	0.049
		Dexamethasone	21	1.7	1.4	14	67	0.62	0.15	0.082	3.2		0.46	0.086
	D	Ctrl .1	35	3.1	2.3	27	232	0.51	0.15	0.10	1.8		1.2	0.18
		Ctrl. 2	30	3.2	3.2	31	247	0.68	0.15	0.10	2.1	< LOD	1.0	0.15
		Celecoxib	13	1.5	4.0	29	296	0.55	0.12	0.080	1.8		0.89	0.11
	E	Ctrl .1	41	1.9	3.0	41	435	0.91	0.15	0.075	1.0		4.5	0.15
		Ctrl. 2	35	2.2	2.0	31	344	0.78	0.13	0.054	0.6	< LOD	3.3	0.14
		Dexamethasone	23	0.91	3.5	50	516	1.3	0.10	0.12	1.0		9.6	0.23
		ML351	51	3.9	1.7	27	233	0.81	0.19	0.032	0.8		4.4	0.13
	F	Ctrl .1	17	1.2	1.9	21	368	0.72	0.10	0.10	2.7		2.0	0.48
		Ctrl. 2	14	0.71	2.4	19	295	0.75	0.11	0.13	3.0		2.3	0.52
		Indomethacin	0.84	< LOD	2.5	19	291	0.83	0.070	0.086	2.7	< LOD	2.3	0.40
		Dexamethasone	8.6	0.51	2.8	26	389	0.75	0.032	0.084	2.6		2.7	0.53
		ML351	16	0.95	2.1	8.7	202	0.79	0.070	< LOD	2.7		2.0	0.29
	G	Ctrl .1	45	2.7	2.3	27	346	0.75	0.24	0.056	1.1		4.0	0.16
		Ctrl. 2	41	2.5	2.0	31	362	0.85	0.28	0.043	0.9		4.4	0.17
		Indomethacin	3.8	0.086	3.0	39	441	1.4	0.37	0.10	3.3	< LOD	5.9	0.21
		Dexamethasone	53	3.5	2.9	45	444	1.1	0.24	0.10	1.5		5.2	0.14
		Celecoxib	31	2.8	3.9	59	493	0.88	0.18	0.080	2.0		3.0	0.10
		ML351	100	7.9	1.3	17	200	1.3	0.48	< LOD	2.2		4.5	0.12
	H	Ctrl .1	55	4.3	1.6	23	309	1.3	0.36	0.081	2.2		6.8	0.40
		Ctrl. 2	53	4.5	1.8	23	356	1.3	0.38	0.082	2.9	< LOD	4.2	0.34
		Indomethacin	5.9	1.0	2.1	21	290	0.94	0.20	0.085	2.5		2.7	0.34
	I	Ctrl. [1]	26	0.062	1.5	13	136	1.3	0.39	0.12	2.0	< LOD	3.4	0.21
		Celecoxib[1]	15	0.052	2.1	18	174	1.2	0.37	0.11	1.9		4.5	0.20

			(A) ii) Relative oxylipin conc (% of ctrl)					(B) ii) Relative protein levels (% of ctrl)						
	Donor	Incubation	12-HHT	PGE_2	5-HETE	12-HETE	15-HETE	COX-1	COX-2	5-LOX	FLAP	12-LOX	15-LOX	15-LOX-2
M1 + 1 µg/mL LPS	A	Ctrl. 1	96	93	89	121	118	77	85	77	74			
		Ctrl. 2	104	107	111	79	82	123	115	123	126			
		Indomethacin	8	12	92	70	7	124	140	118	124	< LOD	< LOD	< LOD
		Dexamethasone	90	117	92	96	57	118	53	142	118			
		PF4191834	105	133	89	59	45	131	96	232	132			
	B	Ctrl. 1	99	104	92	108	104	92	90	86	87			
		Ctrl. 2	101	96	108	92	96	108	110	114	113			
		Indomethacin	21	16	92	65	8	143	150	147	147	< LOD	< LOD	< LOD
		Dexamethasone	66	49	101	54	53	197	101	218	206			
		PF4191834	72	80	97	13	63	167	118	302	160			
	C	Ctrl. 1	116	113	94	110	116	111	116	91	102			
		Ctrl. 2	84	87	106	90	84	89	84	109	98			
		Indomethacin	14	12	97	185	6	107	102	84	102	< LOD	< LOD	< LOD
		Dexamethasone	60	68	158	83	55	88	36	125	95			
		PF4191834	153	131	61	57	78	103	79	126	92			
	D	Ctrl. 1	112	112	107	78	99	108	113	111	118			
		Ctrl. 2	88	88	93	122	101	92	87	89	82			
		Indomethacin	13	9	111	162	6	83	90	81	68	< LOD	< LOD	< LOD
		Dexamethasone	42	44	307	101	32	97	38	160	89			
		PF4191834	97	70	74	160	73	90	65	141	53			
M2 +1 µg/mL LPS	A	Ctrl .1	100	107	94	99	101	94	96	105	96		98	103
		Ctrl. 2	100	93	106	101	99	106	104	95	104	< LOD	102	97
		Dexamethasone	65	102	119	116	112	98	52	112	86		101	125
	B	Ctrl .1	85	91	76	103	96	95	104	78	91		97	96
		Ctrl. 2	115	109	124	97	104	105	96	122	109	< LOD	103	104
		ML351	109	122	95	49	63	138	186	20	119		124	142
	C	Ctrl .1	98	91	107	91	92	130	113	119	117		123	124
		Ctrl. 2	102	109	93	109	108	70	87	81	83	< LOD	77	76
		Dexamethasone	75	65	102	78	111	107	66	152	110		146	135
	D	Ctrl .1	107	97	85	93	97	85	100	98	92		111	109
		Ctrl. 2	93	103	115	107	103	115	100	102	108	< LOD	89	91
		Celecoxib	40	46	147	98	124	93	82	80	92		83	68
	E	Ctrl .1	108	92	120	115	112	107	109	116	125		115	102
		Ctrl. 2	92	108	80	85	88	93	91	84	75	< LOD	85	98
		Dexamethasone	60	44	138	139	133	156	71	185	133		245	160
		ML351	133	191	65	75	60	96	138	25	110		113	89
	F	Ctrl .1	110	125	88	105	111	97	99	85	95		92	96
		Ctrl. 2	90	75	112	95	89	103	101	115	105		108	104
		Indomethacin	5	5	116	93	88	113	66	76	96	< LOD	108	80
		Dexamethasone	56	53	128	128	117	102	30	74	93		125	107
		ML351	103	100	97	43	61	108	67	14	95		93	59
	G	Ctrl .1	104	105	107	93	98	94	91	113	113		96	95
		Ctrl. 2	96	95	93	107	102	106	109	87	87		104	105
		Indomethacin	9	2	136	135	124	175	141	199	334	< LOD	142	125
		Dexamethasone	122	133	131	155	125	138	94	195	147		124	84
		Celecoxib	71	109	179	207	139	110	68	161	198		72	61
		ML351	231	303	61	58	56	157	184	33	221		106	74
	H	Ctrl .1	101	98	93	98	93	99	98	99	86		124	108
		Ctrl. 2	99	102	107	102	107	101	102	101	114	< LOD	76	92
		Indomethacin	11	23	123	91	87	72	53	104	100		49	92
	I	Ctrl. [1]	100	100	100	100	100	100	100	100	100	< LOD	100	100
		Celecoxib[1]	57	84	137	141	128	97	94	98	94		130	97

	(A) iii) Mean of relative oxylipin conc (% of ctrl)					(B) iii) Mean of relative protein levels (% of ctrl)						
**M1 + LPS**	**12-HHT**	**PGE_2**	**5-HETE**	**12-HETE**	**15-HETE**	**COX-1**	**COX-2**	**5-LOX**	**FLAP**	**12-LOX**	**15-LOX**	**15-LOX-2**
Indomethacin	14 ± 3	12 ± 1	98 ± 4	121 ± 31	7 ± 1	114 ± 13	120 ± 14	107 ± 16	110 ± 17			
Dexamethasone	52 ± 10	56 ± 17	132 ± 50	67 ± 11	39 ± 6	125 ± 25	57 ± 15	161 ± 20	127 ± 27	< LOD	< LOD	< LOD
PF4191834	107 ± 17	103 ± 17	80 ± 8	72 ± 31	65 ± 7	123 ± 17	90 ± 11	200 ± 41	109 ± 23			
**M2 + LPS**												
Indomethacin	8 ± 2	10 ± 6	125 ± 6	106 ± 14	100 ± 12	120 ± 30	87 ± 28	126 ± 37	177 ± 79		100 ± 27	99 ± 13
Dexamethasone	76 ± 18	80 ± 21	124 ± 23	123 ± 25	120 ± 22	120 ± 12	63 ± 11	144 ± 23	114 ± 12	< LOD	148 ± 25	122 ± 13
Celecoxib	56 ± 9	80 ± 18	154 ± 13	149 ± 32	130 ± 5	100 ± 5	81 ± 8	113 ± 25	128 ± 35		95 ± 18	76 ± 11
ML351	144 ± 30	179 ± 46	80 ± 10	56 ± 7	60 ± 1	125 ± 14	144 ± 28	23 ± 4	136 ± 29		109 ± 6	91 ± 18

≤ 25% **≤ 50%** **≤ 75%** **≥ 125%** **≥150%** **≥175%** **≥200%** of control

[1]: only one control per donor

4 MRM³ analysis

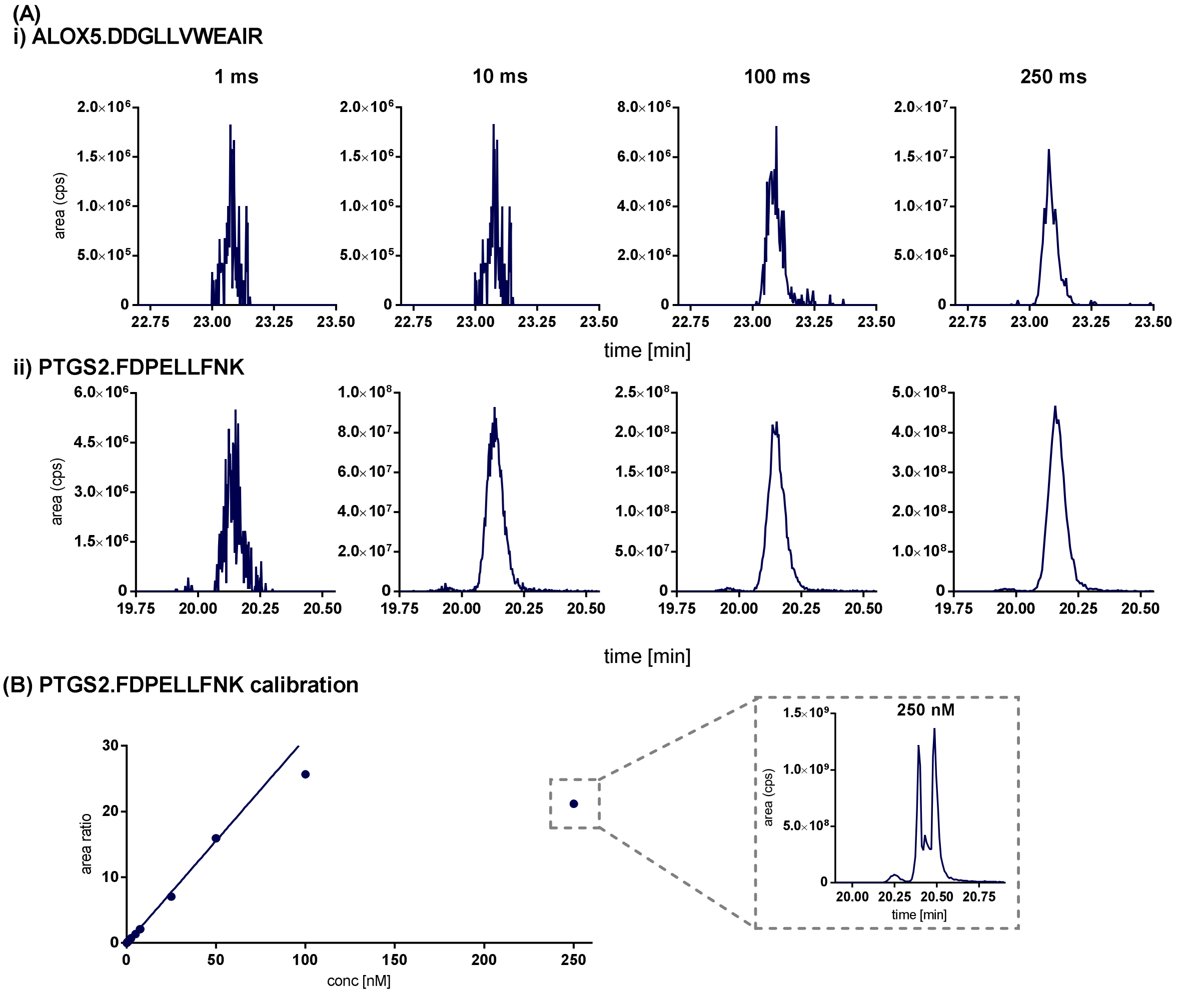

ESM Fig. S1 Optimization of QTRAP fill time for MS³ experiments and evaluation of linear range in MS³. (A) Longer fixed fill times (FFT) result in increased signal intensity and thus, improved signal-to noise ratios. Shown are 25 nM standards of **(A)i)** DDGLLVWEAIR (5-LOX) and **(A)ii)** FDPELLFNK (COX-2). **(B)** The calibration range in MS³ is limited due to overfilling of the ion trap at higher concentrations resulting in poor peak shape, shown exemplarily for the COX-2 peptide FDPELLFNK.

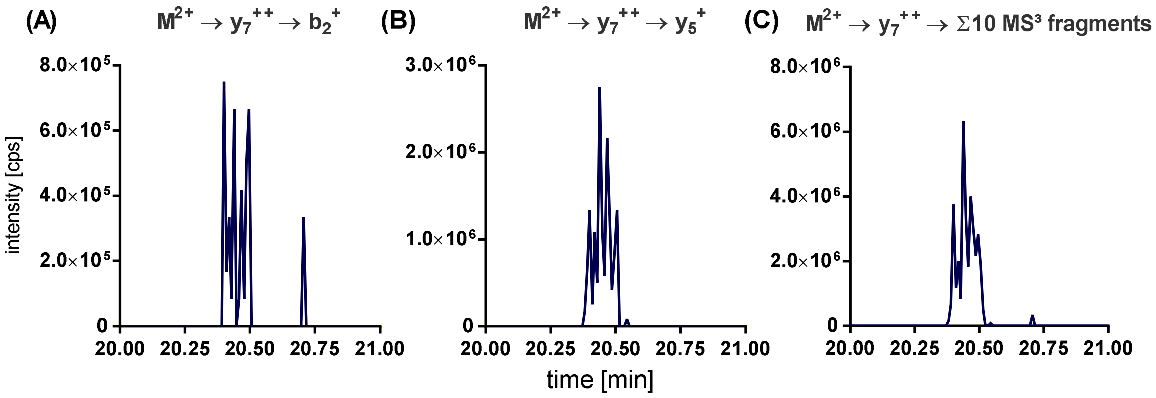

ESM Fig. S2 Improving MRM³ analysis. Summing multiple MS³ fragments improves sensitivity for analysis and thus enables lower LLOQs in MRM³ analysis. Shown is a standard of FDPELLFNK (COX-2; 84 pM) measured in MRM³ mode. The signal intensities of **(A)**, **(B)** individually isolated MS³ fragments is lower compared to **(C)** the sum of 10 MS³ fragments.

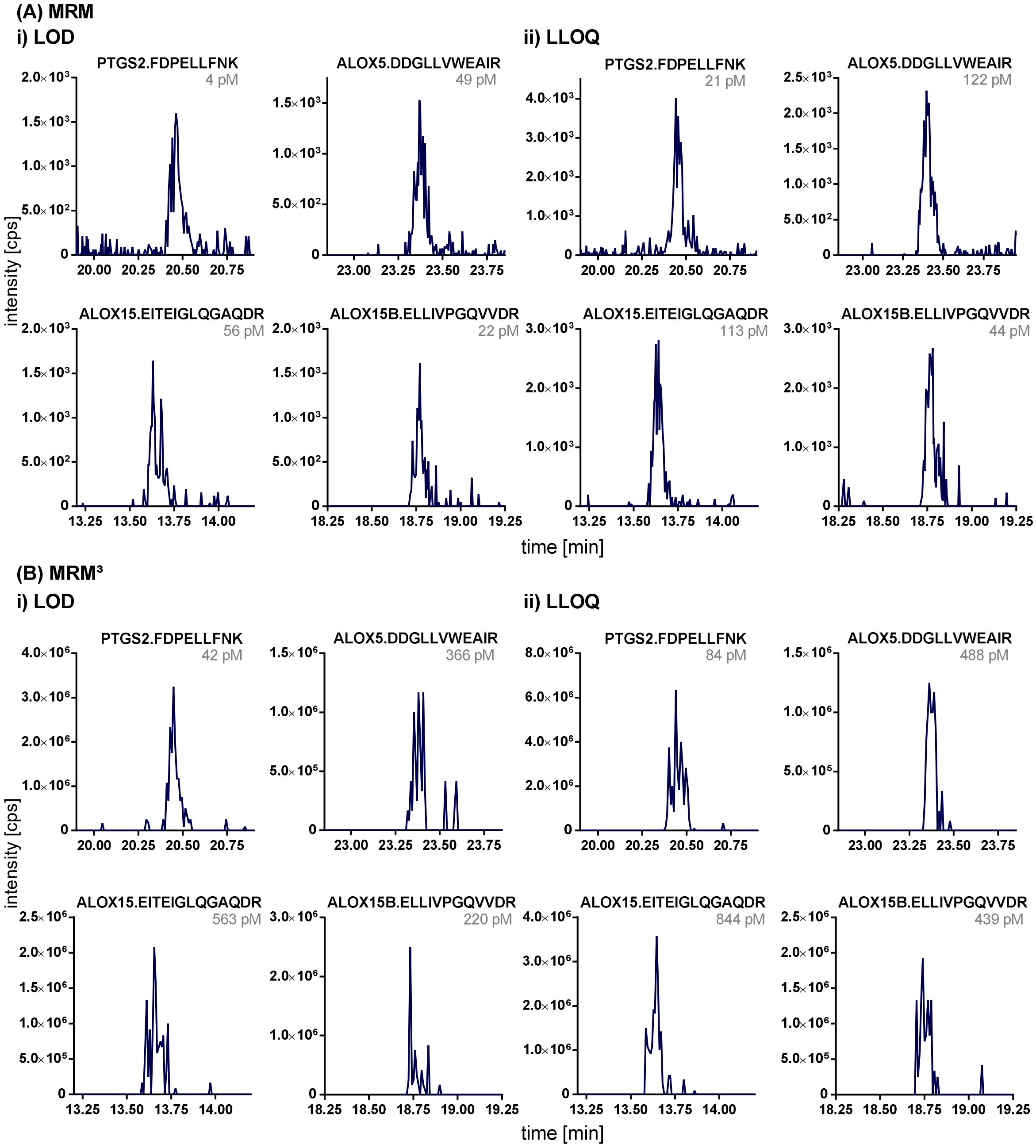

ESM Fig. S3 Comparison of MRM and MRM³ sensitivities. Comparison of **(A)** MRM and **(B)** MRM³ modes regarding **i)** limits of detection (LOD) and **ii)** lower limits of quantification (LLOQ) for peptides of COX-2 (FDPELLFNK), 5-LOX (DDGLLVWEAIR), 15-LOX (EITEIGLQGAQDR) and 15-LOX-2 (ELLIVPGQVVDR). LOD was set to $S/N \geq 3$ and LLOQ to $S/N \geq 5$ and accuracies within ± 20%.

5 Cell viability assays

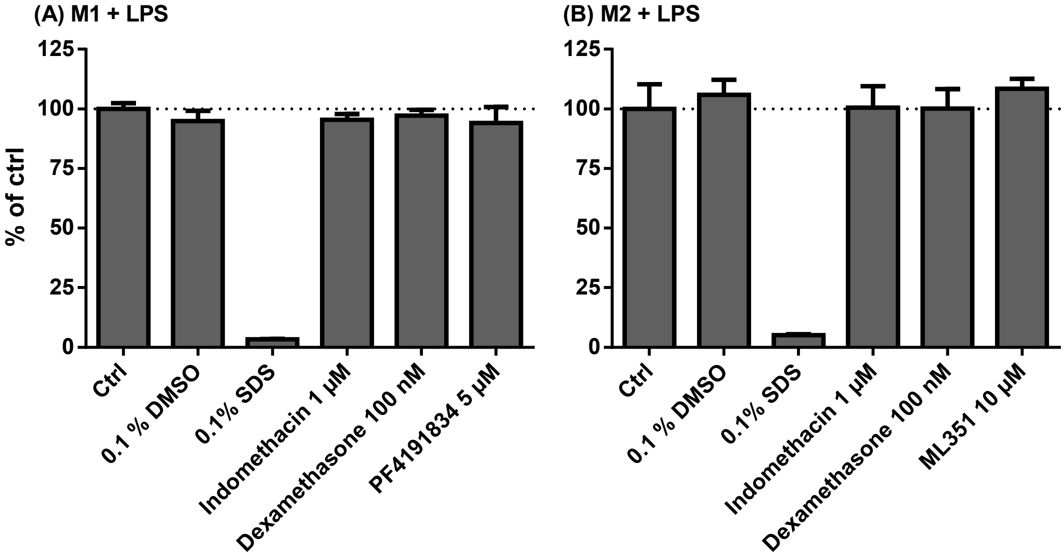

ESM Fig. S4 Resazurin assay. Cell viability was determined by resazurin assay in human primary macrophages. Cells were differentiated with **(A)** 10 ng mL^{-1} CSF-2 (M1-like cells) or **(B)** CSF-1 (M2-like cells) for 8 days and with 10 ng mL^{-1} IFNγ (M1-like cells) or IL-4 (M2-like cells) for the final 48 h. The cells were incubated with the different test compounds at the indicated concentrations for the final 7 h during additional 1 µg mL^{-1} LPS stimulation for the final 6 h. DMSO served as vehicle control and SDS as positive control. Dehydrogenase activity was measured as resorufin formation by fluorometric readout at 590 nm after excitation at 560 nm (13). Shown are mean ± SD for n = 6-12 technical replicates from a pool of 5 donors.

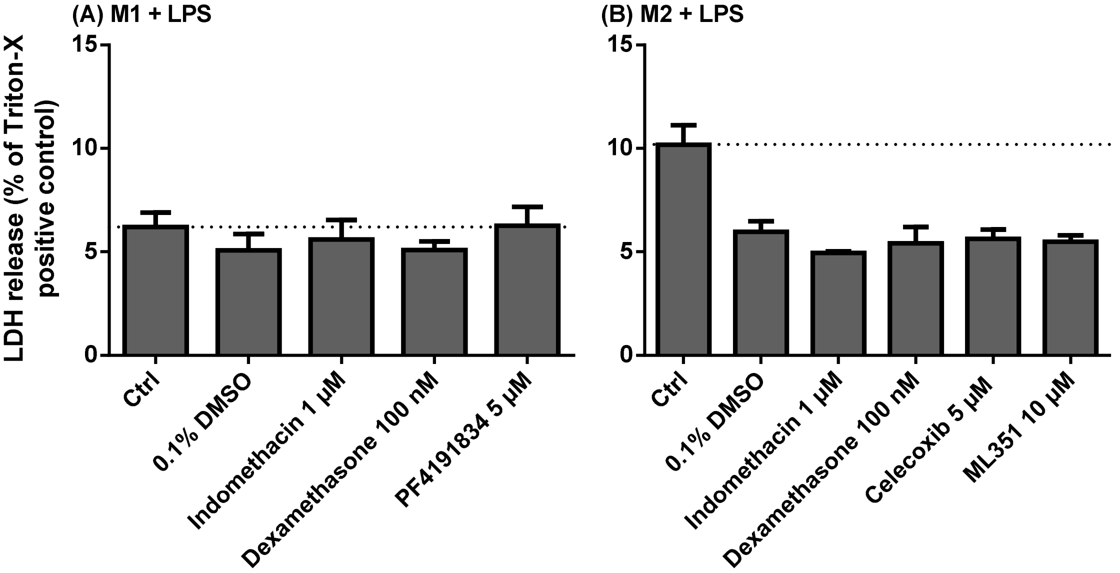

ESM Fig. S5 Lactate dehydrogenase assay. Cell viability was determined by lactate dehydrogenase assay in human primary macrophages. Cells were differentiated with **(A)** 10 ng mL^{-1} CSF-2 (M1-like cells) or **(B)** CSF-1 (M2-like cells) for 8 days and with 10 ng mL^{-1} IFNγ (M1-like cells) or IL-4 (M2-like cells) for the final 48 h. The cells were incubated with the different test compounds at the indicated concentrations for the final 7 h during additional 1 µg mL^{-1} LPS stimulation for the final 6 h. 0.2% Triton-X served as positive control and DMSO as vehicle control. Dehydrogenase activity was measured via the absorbance decrease at 340 nm for 45 minutes during the NADH dependent reduction of pyruvate to lactate. LDH leakage was estimated by comparing LDH activities in culture medium and lysed cells. Shown are mean ± SD for n = 3-4 technical replicates from a pool of 3 donors.

6 References

1. Koch E, Mainka M, Dalle C, Ostermann AI, Rund KM, Kutzner L, et al. Stability of oxylipins during plasma generation and long-term storage. Talanta. 2020;217:121074.
2. Rund KM, Ostermann AI, Kutzner L, Galano JM, Oger C, Vigor C, et al. Development of an LC-ESI(-)-MS/MS method for the simultaneous quantification of 35 isoprostanes and isofurans derived from the major n3- and n6-PUFAs. Anal Chim Acta. 2018;1037:63-74.
3. Kutzner L, Rund KM, Ostermann AI, Hartung NM, Galano JM, Balas L, et al. Development of an Optimized LC-MS Method for the Detection of Specialized Pro-Resolving Mediators in Biological Samples. Front Pharmacol. 2019;10.
4. Hartung NM, Mainka M, Kampschulte N, Ostermann AI, Schebb NH. A strategy for validating concentrations of oxylipin standards for external calibration. Prostaglandins Other Lipid Mediat. 2019;141:22-4.
5. Johnson M, Zaretskaya I, Raytselis Y, Merezhuk Y, McGinnis S, Madden TL. NCBI BLAST: a better web interface. Nucleic Acids Res. 2008;36(suppl_2):W5-W9.
6. Schaeffer M, Gateau A, Teixeira D, Michel PA, Zahn-Zabal M, Lane L. The neXtProt peptide uniqueness checker: a tool for the proteomics community. Bioinformatics. 2017;33(21):3471-2.
7. Gasteiger E, Hoogland C, Gattiker A, Duvaud Se, Wilkins MR, Appel RD, et al. Protein Identification and Analysis Tools on the ExPASy Server. In: Walker JM, editor. The Proteomics Protocols Handbook. Totowa, NJ: Humana Press; 2005. p. 571-607.
8. Fannes T, Vandermarliere E, Schietgat L, Degroeve S, Martens L, Ramon J. Predicting Tryptic Cleavage from Proteomics Data Using Decision Tree Ensembles. J Proteome Res. 2013;12(5):2253-9.
9. The Unitprot Consortium. UniProt: a worldwide hub of protein knowledge. Nucleic Acids Res. 2018;47(D1):D506-D15.
10. Hornbeck PV, Zhang B, Murray B, Kornhauser JM, Latham V, Skrzypek E. PhosphoSitePlus, 2014: mutations, PTMs and recalibrations. Nucleic Acids Res. 2015;43(D1):D512-D20.
11. Krokhin OV, Spicer V. Peptide Retention Standards and Hydrophobicity Indexes in Reversed-Phase High-Performance Liquid Chromatography of Peptides. Anal Chem. 2009;81(22):9522-30.

12. Hartung NM, Ostermann AI, Immenschuh S, Schebb NH. Combined Targeted Proteomics and Oxylipin Metabolomics for Monitoring of the COX-2 Pathway. PROTEOMICS. 2021;21(3-4):1900058.

13. O'Brien J, Wilson I, Orton T, Pognan F. Investigation of the Alamar Blue (resazurin) fluorescent dye for the assessment of mammalian cell cytotoxicity. Eur J Biochem. 2000;267(17):5421-6.
