# Supplementary material for: Development of a quantitative proteomics approach for cyclooxygenases and lipoxygenases in parallel to quantitative oxylipin analysis allowing the comprehensive investigation of the arachidonic acid cascade
Source: Anal Bioanal Chem. 2023 Jan 23;415(5):913–33. doi: 10.1007/s00216-022-04489-3 (PMC9883352; doi:10.1007/s00216-022-04489-3)
Supplement: Supplementary file 2 — Supplementary file2 (PDF 522 kb) [file 216_2022_4489_MOESM2_ESM.pdf]

**ESM Table S14 Targeted oxylipin metabolomics method.**

| precursor PUFA | PUFA class | analyte | MS transition Q1 | Q3 | MS parameters DP [V] | EP [V] | CE [V] | CXP [V] | internal standard | RT [min] | calibration range LOD [1] [nM] | LLOQ [2] [nM] | ULOQ [3] | Reference |
|---|---|---|---|---|---|---|---|---|---|---|---|---|---|---|
| Oleic acid (18:1 *n*-9) | epoxy-PUFA | 9(10)-Ep-stearic acid | 297.0 | 170.8 | -120 | -10 | -24 | -11 | $^{2}H_{4}$-12(13)-EpOME | 23.91 | 0.25 | 0.5 | 500 | Koch (2020) Talanta |
| | | *trans*-9(10)-Ep-stearic acid | 297.0 | 170.8 | -120 | -10 | -24 | -11 | $^{2}H_{4}$-12(13)-EpOME | 24.06 | | relative quantification based on 9(10)-Ep-stearic acid | | Rund (2019) POLM |
| | vic dihydroxy-PUFA | erythro-9,10-DiH-stearic acid | 315.2 | 170.8 | -85 | -10 | -35 | -9 | $^{2}H_{4}$-9,10-DiHOME | 16.34 | 0.25 | 0.50 | 1000 | current paper |
| | | threo-9,10-DiH-stearic acid | 315.2 | 170.8 | -85 | -10 | -35 | -9 | $^{2}H_{4}$-9,10-DiHOME | 17.29 | 0.25 | 0.50 | 1000 | current paper |
| Linoleic Acid (LA; 18:2 *n*-6) | hydroxy-PUFA | 9-HODE | 295.2 | 171.1 | -100 | -10 | -24 | -7 | $^{2}H_{4}$-9-HODE | 19.47 | 0.14 | 0.35 | 348 | Koch (2020) Talanta |
| | | 10-HODE | 295.1 | 183.0 | -90 | -10 | -25 | -7 | $^{2}H_{4}$-9-HODE | 19.40 | 0.038 | 0.076 | 380 | Koch (2020) Talanta |
| | | 12-HODE | 295.1 | 183.0 | -80 | -10 | -24 | -9 | $^{2}H_{4}$-13-HODE | 19.02 | 0.025 | 0.05 | 100 | Koch (2020) Talanta |
| | | 13-HODE | 295.2 | 195.2 | -100 | -10 | -24 | -9 | $^{2}H_{4}$-9-HODE | 19.33 | 0.1 | 0.25 | 500 | Koch (2020) Talanta |
| | | 15-HODE | 295.2 | 223.0 | -95 | -10 | -24 | -9 | $^{2}H_{4}$-13-HODE | 18.19 | 0.089 | 0.18 | 177 | Koch (2020) Talanta |
| | | $^{2}H_{4}$-13-HODE | 299.2 | 198.1 | -80 | -10 | -24 | -9 | *internal standard* | 19.21 | | | | Koch (2020) Talanta |
| | | $^{2}H_{4}$-9-HODE | 299.2 | 172.3 | -100 | -10 | -24 | -6 | *internal standard* | 19.35 | | | | Koch (2020) Talanta |
| | oxo-PUFA | 9-oxo-ODE | 293.2 | 185.1 | -115 | -10 | -26 | -8 | $^{2}H_{3}$-13-oxo-ODE | 20.90 | 0.25 | 0.5 | 500 | Koch (2020) Talanta |
| | | 13-oxo-ODE | 293.2 | 113.0 | -100 | -10 | -27 | -8 | $^{2}H_{3}$-13-oxo-ODE | 20.28 | 0.25 | 0.5 | 500 | Koch (2020) Talanta |
| | | $^{2}H_{3}$-13-oxo-ODE | 296.2 | 198.2 | -85 | -10 | -28 | -8 | *internal standard* | 20.19 | | | | Koch (2020) Talanta |
| | epoxy-PUFA | 9(10)-EpOME | 295.2 | 171.1 | -105 | -10 | -19 | -8 | $^{2}H_{4}$-12(13)-EpOME | 22.50 | 0.081 | 0.2 | 405 | Koch (2020) Talanta |
| | | *trans*-9(10)-EpOME | 295.2 | 171.1 | -105 | -10 | -19 | -8 | $^{2}H_{4}$-12(13)-EpOME | 22.65 | | relative quantification based on 9(10)-EpOME | | Rund (2019) POLM |
| | | 12(13)-EpOME | 295.2 | 195.2 | -105 | -10 | -19 | -8 | $^{2}H_{4}$-12(13)-EpOME | 22.29 | 0.019 | 0.037 | 185 | Koch (2020) Talanta |
| | | *trans*-12(13)-EpOME | 295.2 | 195.2 | -105 | -10 | -19 | -8 | $^{2}H_{4}$-12(13)-EpOME | 22.44 | | relative quantification based on 12(13)-EpOME | | Rund (2019) POLM |
| | | $^{2}H_{4}$-12(13)-EpOME | 299.2 | 198.1 | -100 | -10 | -21 | -8 | *internal standard* | 22.19 | | | | Koch (2020) Talanta |
| | vic dihydroxy-PUFA | 9,10-DiHOME | 313.2 | 201.2 | -105 | -10 | -28 | -8 | $^{2}H_{4}$-9,10-DiHOME | 14.94 | 0.0075 | 0.01 | 250 | Koch (2020) Talanta |
| | | 12,13-DiHOME | 313.2 | 183.2 | -105 | -10 | -29 | -8 | $^{2}H_{4}$-9,10-DiHOME | 14.46 | 0.015 | 0.029 | 145 | Koch (2020) Talanta |
| | | $^{2}H_{4}$-9,10-DiHOME | 317.2 | 203.4 | -105 | -10 | -28 | -8 | *internal standard* | 14.84 | | | | Koch (2020) Talanta |
| | misc | 9,10,11-TriHOME | 329.1 | 201.1 | -95 | -10 | -30 | -8 | $^{2}H_{5}$-LxA$_{4}$ | 9.70 | 0.05 | 0.1 | 250 | Koch (2020) Talanta |
| | | 9,10,13-TriHOME | 329.2 | 171.1 | -100 | -10 | -31 | -8 | $^{2}H_{5}$-LxA$_{4}$ | 8.50 | 0.05 | 0.1 | 250 | Koch (2020) Talanta |
| | | 9,12,13-TriHOME | 329.2 | 211.1 | -100 | -10 | -31 | -10 | $^{2}H_{5}$-LxA$_{4}$ | 8.35 | 0.025 | 0.05 | 250 | Koch (2020) Talanta |
| | | EKODE | 309.2 | 291.1 | -90 | -10 | -16 | -6 | $^{2}H_{4}$-9-HODE | 17.23 | | relative quantification based on 12(13)-EpOME | | Ostermann (2015) Anal Bioanal Chem |
| alpha-Linolenic Acid (ALA; 18:3 *n*-3) | hydroxy-PUFA | 9-HOTrE | 293.2 | 171.2 | -85 | -10 | -20 | -8 | $^{2}H_{4}$-13-HODE | 16.88 | 0.1 | 0.25 | 500 | Koch (2020) Talanta |
| | | 13-HOTrE | 293.2 | 195.1 | -90 | -10 | -22 | -8 | $^{2}H_{4}$-13-HODE | 17.24 | 0.25 | 0.5 | 250 | Koch (2020) Talanta |
| | oxo-PUFA | 9-oxo-OTrE | 291.0 | 185.0 | -100 | -10 | -24 | -8 | $^{2}H_{3}$-13-oxo-ODE | 18.34 | 0.1 | 0.25 | 500 | Koch (2020) Talanta |
| | | 13-oxo-OTrE | 291.1 | 195.0 | -100 | -10 | -27 | -8 | $^{2}H_{3}$-13-oxo-ODE | 18.12 | 0.05 | 0.1 | 100 | Koch (2020) Talanta |
| | epoxy-PUFA | 9(10)-EpODE | 293.2 | 171.2 | -90 | -10 | -16 | -8 | $^{2}H_{4}$-12(13)-EpOME | 20.15 | 0.058 | 0.116 | 582 | Koch (2020) Talanta |
| | | 12(13)-EpODE | 293.2 | 183.1 | -90 | -10 | -20 | -8 | $^{2}H_{4}$-12(13)-EpOME | 20.61 | 0.17 | 0.33 | 331 | Koch (2020) Talanta |
| | | 15(16)-EpODE | 293.3 | 235.2 | -90 | -10 | -16 | -4 | $^{2}H_{4}$-12(13)-EpOME | 19.97 | 0.092 | 0.185 | 924 | Koch (2020) Talanta |
| | | *trans*-9(10)-EpODE | 293.2 | 171.2 | -90 | -10 | -16 | -8 | $^{2}H_{4}$-12(13)-EpOME | 20.30 | | relative quantification based on trans-9(10)-EpODE | | Rund (2019) POLM |
| | | *trans*-12(13)-EpODE | 293.2 | 183.1 | -90 | -10 | -20 | -8 | $^{2}H_{4}$-12(13)-EpOME | 20.76 | | relative quantification based on 12(13)-EpODE | | Rund (2019) POLM |
| | | *trans*-15(16)-EpODE | 293.3 | 235.2 | -90 | -10 | -16 | -4 | $^{2}H_{4}$-12(13)-EpOME | 20.12 | | relative quantification based on 15(16)-EpODE | | Rund (2019) POLM |
| | vic dihydroxy-PUFA | 9,10-DiHODE | 311.2 | 201.2 | -90 | -10 | -26 | -10 | $^{2}H_{4}$-9,10-DiHOME | 12.82 | 0.01 | 0.025 | 100 | Koch (2020) Talanta |
| | | 12,13-DiHODE | 311.2 | 183.1 | -105 | -10 | -29 | -8 | $^{2}H_{4}$-9,10-DiHOME | 12.90 | 0.1 | 0.25 | 250 | Koch (2020) Talanta |
| | | 15,16-DiHODE | 311.2 | 223.2 | -105 | -10 | -28 | -10 | $^{2}H_{4}$-9,10-DiHOME | 12.76 | 0.18 | 0.45 | 181 | Koch (2020) Talanta |
| | misc | 9,10,11-TriHODE | 327.0 | 171.0 | -80 | -10 | -25 | -8 | $^{2}H_{5}$-LxA$_{4}$ | 8.34 | 0.025 | 0.05 | 250 | Koch (2020) Talanta |
| | | 9,10,13-TriHODE | 327.2 | 201.0 | -80 | -10 | -28 | -8 | $^{2}H_{5}$-LxA$_{4}$ | 7.35 | 0.75 | 1 | 500 | Koch (2020) Talanta |
| | | 9,12,13-TriHODE | 327.2 | 211.0 | -80 | -10 | -29 | -10 | $^{2}H_{5}$-LxA$_{4}$ | 7.35 | 0.05 | 0.1 | 250 | Koch (2020) Talanta |
| gamma-Linolenic Acid (GLA; 18:3 *n*-6) | hydroxy-PUFA | 13-γ-HOTrE | 293.0 | 193.0 | -90 | -10 | -23 | -8 | $^{2}H_{4}$-LTB$_{4}$ | 17.59 | 1 | 2.5 | 500 | Koch (2020) Talanta |
| dihomo-gamma-Linolenic Acid (DGLA; 20:3 *n*-6) | hydroxy-PUFA | 8-HETrE | 321.2 | 157.1 | -85 | -10 | -22 | -9 | $^{2}H_{8}$-5-HETE | 21.85 | 0.25 | 0.5 | 500 | Koch (2020) Talanta |
| | | 12-HETrE | 321.0 | 181.0 | -85 | -10 | -24 | -10 | $^{2}H_{8}$-5-HETE | 22.02 | 0.1 | 0.25 | 500 | Koch (2020) Talanta |
| | | 15-HETrE | 321.2 | 221.2 | -90 | -10 | -21 | -10 | $^{2}H_{8}$-5-HETE | 21.47 | 0.05 | 0.1 | 500 | Koch (2020) Talanta |
| | multihydroxy-PUFA | LTB$_{3}$ | 337.2 | 195.2 | -80 | -10 | -21 | -8 | $^{2}H_{11}$-11,12-DiHETrE | 15.99 | 0.1 | 0.25 | 500 | Koch (2020) Talanta |
| | epoxy-PUFA | 14(15)-EpEDE | 321.2 | 221.2 | -85 | -10 | -19 | -4 | $^{2}H_{11}$-14(15)-EpETrE | 23.47 | 0.025 | 0.05 | 100 | Koch (2020) Talanta |
| | prostanoids | PGB1 | 335.4 | 221.0 | -85 | -10 | -28 | -7 | $^{2}H_{4}$-15-deoxy-Δ12,14-PGJ$_{2}$ | 12.27 | 0.05 | 0.10 | 750 | current paper |
| | | PGD$_{1}$ | 353.3 | 317.2 | -80 | -10 | -19 | -6 | $^{2}H_{4}$-PGD$_{2}$ | 9.36 | 0.05 | 0.10 | 250 | current paper |
| | | 13,14-dihydro-15-keto-PGD$_{1}$ | 353.3 | 209.0 | -65 | -10 | -31 | -7 | $^{2}H_{4}$-13,14-dihydro-15-keto PGE$_{2}$ | 11.68 | 0.25 | 0.50 | 1000 | current paper |
| | | PGE$_{1}$ | 353.3 | 317.2 | -80 | -10 | -19 | -6 | $^{2}H_{4}$-PGE$_{2}$ | 9.20 | 0.05 | 0.10 | 250 | current paper |
| | | 13,14-dihydro PGE$_{1}$ | 355.4 | 237.1 | -70 | -10 | -37 | -7 | $^{2}H_{4}$-13,14-dihydro-15-keto PGE$_{2}$ | 9.81 | 0.17 | 0.35 | 698 | current paper |
| | | 13,14-dihydro-15-keto-PGE$_{1}$ | 353.3 | 221.2 | -70 | -10 | -29 | -6 | $^{2}H_{4}$-13,14-dihydro-15-keto PGE$_{2}$ | 10.81 | 0.25 | 0.50 | 1000 | current paper |
| | | 15-keto PGE$_{1}$ | 351.3 | 209.0 | -90 | -10 | -31 | -7 | $^{2}H_{4}$-PGE$_{2}$ | 9.96 | 2.50 | 5.00 | 1000 | current paper |
| | | PGF$_{1\alpha}$ | 355.4 | 293.2 | -110 | -10 | -35 | -6 | $^{2}H_{4}$-PGF$_{2\alpha}$ | 8.60 | 0.025 | 0.05 | 250 | Koch (2020) Talanta |
| | | 15-keto-PGF$_{1\alpha}$ | 353.3 | 193.1 | -70 | -10 | -37 | -6 | *theoretically* $^{2}H_{4}$-PGF$_{2\alpha}$ [5] | 9.46 | | relative quantification based on 15-keto PGE$_{1}$ | | current paper |
| | | TxB$_{1}$ | 371.3 | 171.2 | -90 | -10 | -33 | -10 | $^{2}H_{4}$-TxB2 | 7.37 | 0.40 | 0.80 | 1608 | current paper |
| | isoprostanes | 8-*iso*-PGE$_{1}$ | 353.4 | 235.0 | -80 | -10 | -19 | -7 | $^{2}H_{4}$-PGE$_{2}$ | 8.84 | 0.25 | 0.50 | 500 | current paper |
| | | 15-F$_{1t}$-IsoP (8-iso-PGF$_{1\alpha}$) [4] | 355.2 | 211.0 | -100 | -10 | -35 | -8 | $^{2}H_{4}$-PGF$_{2\alpha}$ | 7.53 | 0.75 | 1 | 500 | Koch (2020) Talanta |
| Mead acid (20:3 *n*-9) | hydroxy-PUFA | 5-HETrE | 321.2 | 115.1 | -90 | -10 | -17 | -9 | $^{2}H_{8}$-5-HETE | 23.56 | 0.01 | 0.025 | 250 | Koch (2020) Talanta |
| Arachidonic Acid (ARA; 20:4 *n*-6) | hydroperoxy-PUFA | 5-HpETE | 335.5 | 155.0 | -40 | -10 | -23 | -13 | $^{2}H_{8}$-5-HETE | 22.13 | | relative quantification based on 5-HETE | | Meckelmann (2017) POLM |
| | | 12-HpETE | 335.5 | 153.0 | -40 | -10 | -23 | -13 | $^{2}H_{8}$-12-HETE | 21.38 | | relative quantification based on 12-HETE | | Meckelmann (2017) POLM |
| | | 15-HpETE | 335.5 | 113.3 | -40 | -10 | -23 | -13 | $^{2}H_{8}$-15-HETE | 20.63 | | relative quantification based on 15-HETE | | Meckelmann (2017) POLM |
| | hydroxy-PUFA | 5-HETE | 319.2 | 115.2 | -80 | -10 | -19 | -7 | $^{2}H_{8}$-5-HETE | 21.74 | 0.018 | 0.035 | 350 | Koch (2020) Talanta |
| | | 8-HETE | 319.2 | 155.2 | -80 | -10 | -20 | -6 | $^{2}H_{8}$-12-HETE | 21.10 | 0.094 | 0.23 | 468 | Koch (2020) Talanta |
| | | 9-HETE | 319.2 | 167.2 | -80 | -10 | -21 | -7 | $^{2}H_{8}$-5-HETE | 21.45 | 0.27 | 0.4 | 265 | Koch (2020) Talanta |
| | | 11-HETE | 319.2 | 167.2 | -80 | -10 | -21 | -7 | $^{2}H_{8}$-12-HETE | 20.68 | 0.022 | 0.044 | 219 | Koch (2020) Talanta |
| | | 12-HETE | 319.2 | 179.2 | -80 | -10 | -19 | -8 | $^{2}H_{8}$-12-HETE | 21.10 | 0.1 | 0.25 | 500 | Koch (2020) Talanta |
| | | 15-HETE | 319.2 | 219.2 | -80 | -10 | -18 | -8 | $^{2}H_{8}$-15-HETE | 20.08 | 0.11 | 0.22 | 220 | Koch (2020) Talanta |
| | | 16-HETE | 319.2 | 233.1 | -85 | -10 | -18 | -8 | $^{2}H_{8}$-15-HETE | 18.89 | 0.1 | 0.25 | 500 | Koch (2020) Talanta |
| | | 17-HETE | 319.2 | 247.0 | -85 | -10 | -19 | -8 | $^{2}H_{8}$-15-HETE | 18.71 | 0.1 | 0.25 | 500 | Koch (2020) Talanta |
| | | 18-HETE | 319.2 | 261.0 | -85 | -10 | -20 | -8 | $^{2}H_{8}$-15-HETE | 18.49 | 0.1 | 0.25 | 500 | Koch (2020) Talanta |
| | | 19-HETE | 319.3 | 230.9 | -70 | -10 | -19 | -6 | $^{2}H_{8}$-15-HETE | 17.79 | 1 | 2.5 | 500 | Koch (2020) Talanta |
| | | 20-HETE | 319.2 | 289.1 | -100 | -10 | -22 | -6 | $^{2}H_{6}$-20-HETE | 18.05 | 0.25 | 0.5 | 500 | Koch (2020) Talanta |
| | | tetranor-12-HETE | 265.2 | 109.1 | -70 | -10 | -15 | -8 | $^{2}H_{4}$-9,10-DiHOME | 15.16 | 0.025 | 0.05 | 250 | Koch (2020) Talanta |
| | | 12-HHTrE | 279.1 | 179.0 | -70 | -10 | -15 | -8 | $^{2}H_{11}$-11,12-DiHETrE | 15.62 | 0.25 | 0.5 | 500 | Koch (2020) Talanta |
| | | $^{2}H_{8}$-5-HETE | 327.2 | 116.1 | -80 | -10 | -19 | -8 | *internal standard* | 21.60 | | | | Koch (2020) Talanta |
| | | $^{2}H_{8}$-12-HETE | 327.2 | 184.2 | -85 | -10 | -20 | -8 | *internal standard* | 20.93 | | | | Koch (2020) Talanta |
| | | $^{2}H_{8}$-15-HETE | 327.2 | 226.0 | -90 | -10 | -18 | -8 | *internal standard* | 19.88 | | | | Koch (2020) Talanta |
| | | $^{2}H_{6}$-20-HETE | 325.2 | 295.2 | -90 | -10 | -22 | -6 | *internal standard* | 17.97 | | | | Koch (2020) Talanta |
| | multihydroxy-PUFA | 5(*S*),12(*S*)-DiHETE | 335.2 | 195.1 | -80 | -10 | -21 | -8 | $^{2}H_{4}$-LTB$_{4}$ | 14.40 | 0.025 | 0.05 | 500 | Koch (2020) Talanta |
| | | 5(*S*),15(*S*)-DiHETE | 335.3 | 173.2 | -80 | -10 | -20 | -8 | $^{2}H_{4}$-LTB$_{4}$ | 13.36 | 0.05 | 0.1 | 500 | Koch (2020) Talanta |
| | | 8(*S*),15(*S*)-DiHETE | 335.2 | 235.2 | -90 | -10 | -21 | -4 | $^{2}H_{4}$-LTB$_{4}$ | 12.92 | 0.51 | 1.26 | 253 | Koch (2020) Talanta |
| | | LTB$_{4}$ | 335.2 | 195.1 | -80 | -10 | -22 | -9 | $^{2}H_{4}$-LTB$_{4}$ | 13.83 | 0.05 | 0.1 | 500 | Koch (2020) Talanta |
| | | 6-*trans*-LTB$_{4}$ | 335.2 | 195.1 | -80 | -10 | -22 | -9 | $^{2}H_{4}$-LTB$_{4}$ | 13.36 | 0.1 | 0.25 | 250 | Koch (2020) Talanta |
| | | 6-*trans*-12-*epi*-LTB$_{4}$ | 335.2 | 195.1 | -85 | -10 | -20 | -9 | $^{2}H_{4}$-LTB$_{4}$ | 13.51 | 0.1 | 0.25 | 500 | Koch (2020) Talanta |
| | | 5(*S*),6(*R*)-DiHETE (ARA) | 335.2 | 115.1 | -70 | -10 | -20 | -8 | $^{2}H_{11}$-11,12-DiHETrE | 17.33 | 0.020 | 0.039 | 390 | Koch (2020) Talanta |
| | | 5(*S*),6(*S*)-DiHETE (ARA) | 335.2 | 115.1 | -70 | -10 | -20 | -8 | $^{2}H_{11}$-11,12-DiHETrE | 17.80 | 0.022 | 0.045 | 223 | Koch (2020) Talanta |
| | | 20-OH-LTB$_{4}$ | 351.2 | 195.2 | -100 | -10 | -24 | -8 | $^{2}H_{4}$-8-iso-PGF$_{2\alpha}$ | 6.55 | 0.025 | 0.05 | 500 | Koch (2020) Talanta |
| | | 20-COOH-LTB$_{4}$ | 365.2 | 347.2 | -100 | -10 | -24 | -8 | $^{2}H_{4}$-8-iso-PGF$_{2\alpha}$ | 6.30 | 0.066 | 0.17 | 330 | Koch (2020) Talanta |
| | | 18-COOH-dinor-LTB$_{4}$ | 337.2 | 195.2 | -85 | -10 | -24 | -9 | $^{2}H_{4}$-8-iso-PGF$_{2\alpha}$ | 4.35 | 0.75 | 1.0 | 500 | Koch (2020) Talanta |
| | | 12-oxo-LTB$_{4}$ | 333.2 | 178.9 | -80 | -10 | -20 | -9 | $^{2}H_{4}$-LTB$_{4}$ | 14.98 | 0.1 | 0.25 | 500 | Koch (2020) Talanta |
| | | 5(*S*),6(*R*),15(*S*)-TriHETE (LxA$_{4}$) | 351.2 | 235.1 | -70 | -10 | -18 | -15 | $^{2}H_{5}$-LxA$_{4}$ | 10.15 | 0.1 | 0.25 | 500 | Koch (2020) Talanta |
| | | 5(*S*),6(*S*),15(*S*)-TriHETE (6(*S*)-LxA$_{4}$) | 351.2 | 235.1 | -70 | -10 | -18 | -5 | $^{2}H_{5}$-5(*S*),6(*R*),15(*S*)-TriHETE (LxA$_{4}$) | 10.51 | 0.75 | 1.00 | 1000 | current paper |
| | | 5(*S*),6(*R*),15(*R*)-TriHETE (15(*R*)-LxA4) | 351.2 | 235.1 | -70 | -10 | -18 | -15 | *theoretically* $^{2}H_{5}$-5(*S*),6(*R*),15(*S*)-TriHETE (LxA$_{4}$) [5] | 10.22 | | relative quantification based on 5(*S*),6(*R*),15(*S*)-TriHETE (LxA$_{4}$) | | current paper |
| | | 5(*S*),14(*R*),15(*S*)-TriHEPE (LxB$_{4}$) | 351.2 | 221.0 | -70 | -10 | -21 | -13 | $^{2}H_{5}$-5(*S*),6(*R*),15(*S*)-TriHETE (LxA$_{4}$) | 9.15 | 0.50 | 0.75 | 1000 | current paper |
| | | $^{2}H_{4}$-LTB$_{4}$ | 339.2 | 197.2 | -80 | -10 | -22 | -9 | *internal standard* | 13.76 | | | | Koch (2020) Talanta |
| | | $^{2}H_{5}$-5(*S*),6(*R*),15(*S*)-TriHETE (LxA$_{4}$) | 356.3 | 222.2 | -80 | -10 | -24 | -13 | *internal standard* | 10.09 | | | | Koch (2020) Talanta |
| | oxo-PUFA | 5-oxo-ETE | 317.2 | 273.0 | -90 | -10 | -20 | -6 | $^{2}H_{7}$-5-oxo-ETE | 22.87 | 0.5 | 0.75 | 500 | Koch (2020) Talanta |
| | | 12-oxo-ETE | 317.2 | 153.1 | -90 | -10 | -23 | -8 | $^{2}H_{7}$-5-oxo-ETE | 21.66 | 0.41 | 1.0 | 103 | Koch (2020) Talanta |
| | | 15-oxo-ETE | 317.2 | 113.1 | -90 | -10 | -23 | -8 | $^{2}H_{7}$-5-oxo-ETE | 20.88 | 0.05 | 0.1 | 250 | Koch (2020) Talanta |
| | | $^{2}H_{7}$-5-oxo-ETE | 324.2 | 210.2 | -90 | -10 | -24 | -8 | *internal standard* | 22.77 | | | | Koch (2020) Talanta |
| | epoxy-PUFA | 5(6)-EpETrE | 319.2 | 191.1 | -85 | -10 | -16 | -7 | $^{2}H_{11}$-14(15)-EpETrE | 23.44 | | relative quantification based on 8(9)-EpETrE | | Rund (2018) Analytica Chimica Acta |
| | | 8(9)-EpETrE | 319.2 | 155.2 | -90 | -10 | -16 | -6 | $^{2}H_{11}$-8(9)-EpETrE | 23.16 | 0.25 | 0.5 | 500 | Koch (2020) Talanta |
| | | 11(12)-EpETrE | 319.2 | 167.2 | -85 | -10 | -16 | -7 | $^{2}H_{11}$-8(9)-EpETrE | 22.98 | 0.05 | 0.1 | 500 | Koch (2020) Talanta |
| | | 14(15)-EpETrE | 319.2 | 219.2 | -90 | -10 | -15 | -4 | $^{2}H_{11}$-14(15)-EpETrE | 22.45 | 0.1 | 0.25 | 500 | Koch (2020) Talanta |
| | | *trans*-5(6)-EpETrE | 319.2 | 191.1 | -85 | -10 | -16 | -7 | $^{2}H_{11}$-14(15)-EpETrE | 23.59 | | relative quantification based on 8(9)-EpETrE | | Rund (2019) POLM |
| | | *trans*-8(9)-EpETrE | 319.2 | 155.2 | -90 | -10 | -16 | -6 | $^{2}H_{11}$-8(9)-EpETrE | 23.31 | | relative quantification based on 8(9)-EpETrE | | Rund (2019) POLM |
| | | *trans*-11(12)-EpETrE | 319.2 | 167.2 | -85 | -10 | -16 | -7 | $^{2}H_{11}$-8(9)-EpETrE | 23.13 | | relative quantification based on 11(12)-EpETrE | | Rund (2019) POLM |
| | | *trans*-14(15)-EpETrE | 319.2 | 219.2 | -90 | -10 | -15 | -4 | $^{2}H_{11}$-14(15)-EpETrE | 22.60 | | relative quantification based on 14(15)-EpETrE | | Rund (2019) POLM |
| | | $^{2}H_{11}$-8(9)-EpETrE | 330.2 | 155.0 | -80 | -10 | -16 | -7 | *internal standard* | 23.05 | | | | Koch (2020) Talanta |
| | | $^{2}H_{11}$-11,12-DiHETrE | 348.2 | 167.2 | -85 | -10 | -26 | -8 | *internal standard* | 16.31 | | | | Koch (2020) Talanta |
| | | $^{2}H_{11}$-14(15)-EpETrE | 330.2 | 219.3 | -90 | -10 | -16 | -4 | *internal standard* | 22.32 | | | | Koch (2020) Talanta |
| | vic hydroxy-PUFA | 5,6-DiHETrE | 337.2 | 145.1 | -95 | -10 | -25 | -10 | $^{2}H_{11}$-11,12-DiHETrE | 18.15 | 0.05 | 0.1 | 500 | Koch (2020) Talanta |
| | | 8,9-DiHETrE | 337.2 | 127.1 | -95 | -10 | -29 | -8 | $^{2}H_{11}$-11,12-DiHETrE | 17.16 | 0.034 | 0.068 | 170 | Koch (2020) Talanta |
| | | 11,12-DiHETrE | 337.2 | 167.1 | -90 | -10 | -25 | -8 | $^{2}H_{11}$-11,12-DiHETrE | 16.47 | 0.032 | 0.064 | 160 | Koch (2020) Talanta |
| | | 14,15-DiHETrE | 337.2 | 207.1 | -90 | -10 | -24 | -10 | $^{2}H_{11}$-11,12-DiHETrE | 15.65 | 0.01 | 0.025 | 100 | Koch (2020) Talanta |
| | | $^{2}H_{11}$-11,12-DiHETrE | 348.2 | 167.2 | -85 | -10 | -26 | -8 | *internal standard* | 16.31 | | | | Koch (2020) Talanta |
| | | PGB$_{2}$ | 333.3 | 175.1 | -80 | -10 | -27 | -8 | $^{2}H_{4}$-PGB$_{2}$ | 11.94 | 0.025 | 0.05 | 500 | Koch (2020) Talanta |
| | | $^{2}H_{4}$-PGB$_{2}$ | 337.2 | 179.0 | -80 | -10 | -27 | -8 | *internal standard* | 11.91 | | | | Koch (2020) Talanta |
| | | PGD$_{2}$ | 351.2 | 271.3 | -80 | -10 | -23 | -6 | $^{2}H_{4}$-PGD$_{2}$ | 9.37 | 0.75 | 1.00 | 750 | current paper |
| | | 15(*R*)-PGD$_{2}$ | 351.2 | 271.3 | -80 | -10 | -23 | -6 | *theoretically* $^{2}H_{4}$-PGD$_{2}$ [5] | 9.45 | | relative quantification based on PGD$_{2}$ | | current paper |
| | | Δ12-PGD$_{2}$ | 351.3 | 233.1 | -60 | -10 | -18 | -7 | *theoretically* $^{2}H_{4}$-PGD$_{2}$ [5] | 8.67 | | relative quantification based on PGD$_{2}$ | | current paper |
| | | 13,14-dihydro-15-keto PGD$_{2}$ | 351.2 | 207.0 | -55 | -10 | -25 | -7 | $^{2}H_{4}$-13,14-dihydro-15-keto PGE$_{2}$ | 11.18 | 0.25 | 0.50 | 1000 | current paper |
| | | 13,14-dihydro-15-keto-tetranor-PGD$_{2}$ | 297.2 | 109.0 | -55 | -10 | -31 | -7 | $^{2}H_{4}$-13,14-dihydro-15-keto PGE$_{2}$ | 6.56 | 1.04 | 1.55 | 2070 | current paper |
| | | $^{2}H_{4}$-PGD$_{2}$ | 355.2 | 275.3 | -80 | -10 | -24 | -6 | *internal standard* | 9.35 | | | | current paper |
| | | $^{2}H_{9}$-PGD$_{2}$ | 360.2 | 280.2 | -60 | -10 | -24 | -6 | *internal standard* | 9.29 | | | | Koch (2020) Talanta |
| | | PGE$_{2}$ | 351.2 | 271.3 | -80 | -10 | -23 | -6 | $^{2}H_{4}$-PGE$_{2}$ | 8.91 | 0.25 | 0.50 | 750 | current paper |
| | | 15(*R*)-PGE$_{2}$ | 351.2 | 271.3 | -80 | -10 | -23 | -6 | *theoretically* $^{2}H_{4}$-PGE$_{2}$ [5] | 8.67 | | relative quantification based on PGE$_{2}$ | | current paper |

| | | | | | | | | | | | | | | |
|---|---|---|---|---|---|---|---|---|---|---|---|---|---|---|
| | prostanoids | 11β-PGE$_2$ | 351.3 | 189.1 | -60 | -10 | -24 | -7 | *theoretically* $^2$H$_4$-PGE$_2$ $^{5)}$ | 9.11 | relative quantification based on PGE$_2$ | | | current paper |
| | | 20-OH-PGE$_2$ | 367.2 | 189.1 | -70 | -10 | -27 | -8 | $^2$H$_4$-PGE$_2$ | 3.74 | 0.57 | 1.14 | 2288 | current paper |
| | | 15-keto PGE$_2$ | 349.2 | 235.1 | -80 | -10 | -20 | -7 | $^2$H$_4$-PGE$_2$ | 9.50 | 0.25 | 0.50 | 750 | current paper |
| | | 13,14-dihydro-15-keto-PGE$_2$ | 351.2 | 235.2 | -75 | -10 | -18 | -13 | $^2$H$_4$-13,14-dihydro-15-keto PGE$_2$ | 10.29 | 10.00 | 25.00 | 1000 | current paper |
| | | 13,14-dihydro-15-keto-tetranor-PGE$_2$ | 297.0 | 109.0 | -70 | -10 | -18 | -8 | $^2$H$_4$-13,14-dihydro-15-keto PGE$_2$ | 7.32 | 0.39 | 0.79 | 1579 | current paper |
| | | 1a,1b-dihomo PGE$_2$ | 379.4 | 261.2 | -70 | -10 | -21 | -7 | $^2$H$_4$-PGE$_2$ | 11.40 | 0.03 | 0.06 | 451 | current paper |
| | | $^2$H$_4$-PGE$_2$ | 355.2 | 275.3 | -80 | -10 | -24 | -6 | *internal standard* | 8.88 | | | | current paper |
| | | $^2$H$_9$-PGE$_2$ | 360.2 | 280.1 | -85 | -10 | -24 | -6 | *internal standard* | 8.82 | | | | Koch (2020) Talanta |
| | | $^2$H$_4$-13,14-dihydro-15-keto PGE$_2$ | 355.4 | 239.1 | -65 | -10 | -31 | -7 | *internal standard* | 10.26 | | | | current paper |
| | | PGF$_{2\alpha}$ | 353.2 | 193.0 | -80 | -10 | -33 | -7 | $^2$H$_4$-PGF$_{2\alpha}$ | 8.56 | 0.25 | 0.5 | 500 | Koch (2020) Talanta |
| | | 15(*R*)-PGF$_{2\alpha}$ | 353.2 | 193.0 | -80 | -10 | -33 | -7 | *theoretically* $^2$H$_4$-PGF$_{2\alpha}$ $^{5)}$ | 8.48 | relative quantification based on PGF$_{2\alpha}$ | | | current paper |
| | | 11β-PGF$_{2\alpha}$ | 353.3 | 193.1 | -70 | -10 | -35 | -12 | $^2$H$_4$-PGE$_2$ | 7.82 | 0.50 | 0.75 | 1000 | current paper |
| | | 20-OH PGF$_{2\alpha}$ | 369.3 | 193.0 | -70 | -10 | -37 | -7 | $^2$H$_4$-PGE$_2$ | 3.59 | 1.06 | 1.59 | 2121 | current paper |
| | | 13,14-dihydro-PGF$_{2\alpha}$ | 355.4 | 193.0 | -90 | -10 | -34 | -7 | $^2$H$_4$-13,14-dihydro-15-keto PGE$_2$ | 9.53 | 5.00 | 10.00 | 1000 | current paper |
| | | 15-keto PGF$_{2\alpha}$ | 351.2 | 219.1 | -60 | -10 | -23 | -7 | $^2$H$_4$-PGE$_2$ | 9.17 | 0.50 | 0.75 | 750 | current paper |
| | | 13,14-dihydro-15-keto-PGF$_{2\alpha}$ | 353.3 | 183.3 | -100 | -10 | -35 | -10 | $^2$H$_4$-13,14-dihydro-15-keto PGE$_2$ | 10.28 | 0.75 | 1.00 | 1000 | current paper |
| | | 11β-13,14-dihydro-15-keto PGF$_{2\alpha}$ | 353.4 | 195.0 | -85 | -10 | -34 | -7 | $^2$H$_4$-13,14-dihydro-15-keto PGE$_2$ | 9.83 | 18.25 | 45.62 | 1825 | current paper |
| | | 2,3-dinor-11β-PGF$_{2\alpha}$ | 325.3 | 163.0 | -65 | -10 | -19 | -7 | $^2$H$_4$-PGE$_2$ | 5.93 | 0.65 | 1.31 | 2613 | current paper |
| | | $^2$H$_4$-PGF$_{2\alpha}$ | 357.2 | 197.0 | -80 | -10 | -33 | -7 | *internal standard* | 8.54 | | | | Koch (2020) Talanta |
| | | 6-keto-PGF$_{1\alpha}$ | 369.3 | 163.2 | -90 | -10 | -35 | -6 | $^2$H$_4$-6-keto-PGF$_{1\alpha}$ | 6.11 | 0.64 | 0.96 | 640 | Koch (2020) Talanta |
| | | 2,3-dinor-6-keto PGF$_{1\alpha}$ | 341.1 | 135.0 | -90 | -10 | -31 | -7 | $^2$H$_4$-PGE$_2$ | 7.34 | 0.15 | 0.25 | 800 | current paper |
| | | 6,15-diketo-13,14-dihydro PGF$_{1\alpha}$ | 369.3 | 267.0 | -70 | -10 | -31 | -7 | $^2$H$_4$-PGE$_2$ | 7.72 | 45.49 | 75.82 | 3033 | current paper |
| | | $^2$H$_4$-6-keto-PGF$_{1\alpha}$ | 373.0 | 167.0 | -100 | -10 | -35 | -8 | *internal standard* | 6.08 | | | | Koch (2020) Talanta |
| | | PGJ$_2$ | 333.3 | 189.2 | -80 | -10 | -24 | -8 | $^2$H$_4$-PGB$_2$ | 11.83 | 0.014 | 0.027 | 135 | Koch (2020) Talanta |
| | | Δ12-PGJ$_2$ | 333.3 | 189.2 | -70 | -10 | -21 | -8 | $^2$H$_4$-15-deoxy-Δ12,14-PGJ$_2$ | 11.89 | 0.86 | 1.28 | 1712 | current paper |
| | | 15-deoxy-Δ$^{12,14}$-PGJ$_2$ | 315.2 | 203.1 | -90 | -10 | -28 | -7 | $^2$H$_4$-15-deoxy-Δ12,14-PGJ$_2$ | 17.73 | 0.75 | 1.00 | 1000 | current paper |
| | | $^2$H$_4$-15-deoxy-Δ$^{12,14}$-PGJ$_2$ | 319.4 | 203.0 | -80 | -10 | -31 | -7 | *internal standard* | 17.68 | | | | current paper |
| | | TxB$_2$ | 369.2 | 169.1 | -80 | -10 | -24 | -7 | $^2$H$_4$-TxB2 | 7.68 | 0.25 | 0.50 | 1000 | current paper |
| | | 2,3-dinor-TxB$_2$ | 341.2 | 167.0 | -70 | -10 | -14 | -8 | $^2$H$_4$-TxB2 | 5.68 | 1.00 | 2.50 | 1000 | current paper |
| | | 2,3-dinor-TxB$_1$ | 343.0 | 142.9 | -70 | -10 | -18 | -8 | $^2$H$_4$-TxB2 | 5.17 | 1.00 | 2.50 | 750 | current paper |
| | | 11-dehydro-2,3-dinor-TxB$_2$ | 339.3 | 133.0 | -55 | -10 | -20 | -7 | $^2$H$_4$-TxB$_2$ | 6.89 | 0.57 | 0.76 | 762 | current paper |
| | | 11-dehydro-TxB$_2$ | 367.0 | 161.1 | -70 | -10 | -26 | -8 | $^2$H$_4$-TxB$_2$ | 9.02 | 0.21 | 0.31 | 412 | current paper |
| | | $^2$H$_4$-TxB$_2$ | 373.3 | 173.2 | -85 | -10 | -23 | -8 | *internal standard* | 7.66 | | | | current paper |
| | isoprostanes | 8-*iso*-PGE$_2$ | 351.4 | 271.2 | -55 | -10 | -23 | -7 | $^2$H$_4$-PGE$_2$ | 8.69 | 0.10 | 0.25 | 500 | current paper |
| | | 8-*iso*-15-keto PGE$_2$ | 349.4 | 235.0 | -65 | -10 | -19 | -7 | *theoretically* $^2$H$_4$-PGE$_2$ $^{5)}$ | 9.47 | relative quantification based on 15-keto PGE$_2$ | | | current paper |
| | | 15-F$_{2t}$-IsoP (8-iso-PGF$_{2\alpha}$) $^{4)}$ | 353.1 | 193.1 | -95 | -10 | -34 | -8 | $^2$H$_4$-8-iso-PGF$_{2\alpha}$ | 7.58 | 0.10 | 0.25 | 500 | Koch (2020) Talanta |
| | | 8-iso-15(*R*)-PGF$_{2\alpha}$ | 353.2 | 193.0 | -80 | -10 | -33 | -7 | *theoretically* $^2$H$_4$-PGF$_{2\alpha}$ $^{5)}$ | 8.48 | relative quantification based on 8-iso-15(*R*)-PGF$_{2\alpha}$ | | | current paper |
| | | 5(*R,S*)-5-F$_{2c}$-IsoP (8,12-*iso*-iPF$_{2\alpha}$-VI) $^{4)}$ | 353.2 | 219.2 | -90 | -10 | -30 | -8 | $^2$H$_{11}$-8,12-*iso*-iPF$_{2\alpha}$-VI | 10.07 | 0.25 | 0.5 | 500 | Koch (2020) Talanta |
| | | 13,14-dihydro-15-oxo-15-F$_{2t}$-IsoP $^{4)}$ | 353.2 | 291.1 | -110 | -10 | -30 | -8 | $^2$H$_4$-PGF$_{2\alpha}$ | 8.80 | 0.25 | 0.50 | 500 | Koch (2020) Talanta |
| | | 15-oxo-15-F$_{2t}$-IsoP $^{4)}$ | 351.2 | 219.0 | -75 | -10 | -23 | -8 | $^2$H$_4$-8-iso-PGF$_{2\alpha}$ | 8.19 | 0.3 | 0.5 | 500 | Koch (2020) Talanta |
| | | 2,3-dinor-15-(*R,S*)-15-F$_{2t}$-IsoP $^{4)}$ | 325.2 | 237.0 | -70 | -10 | -18 | -8 | $^2$H$_4$-8-iso-PGF$_{2\alpha}$ | 5.49 | 0.100 | 0.25 | 500 | Koch (2020) Talanta |
| | | 5(*R,S*)-5-F$_{2t}$-IsoP (5-iPF$_{2\alpha}$-VI) $^{4)}$ | 353.2 | 114.8 | -85 | -10 | -27 | -8 | $^2$H$_4$-8-iso-PGF$_{2\alpha}$ | 8.07 | 0.1 | 0.25 | 500 | Koch (2020) Talanta |
| | | $^2$H$_{11}$-8,12-*iso*-iPF$_{2\alpha}$-VI | 364.2 | 320.1 | -90 | -10 | -30 | -8 | *internal standard* | 10.01 | | | | Koch (2020) Talanta |
| | | $^2$H$_4$-8-iso-PGF$_{2\alpha}$ | 357.2 | 196.8 | -75 | -10 | -34 | -8 | *internal standard* | 7.55 | | | | Koch (2020) Talanta |
| | misc | 20-COOH-ARA | 333.2 | 271.0 | -95 | -10 | -23 | -6 | $^2$H$_6$-20-HETE | 17.13 | 0.1 | 0.25 | 500 | Koch (2020) Talanta |
| | | 11,12,15-TriHETrE | 353.2 | 167.1 | -100 | -10 | -27 | -10 | $^2$H$_4$-PGE$_2$ | 10.16 | relative quantification based on LXA$_4$ | | | Rund (2018) Analytica Chimica Acta |
| Eicosapentaenoic Acid (EPA; 20:5 *n*-3) | hydroxy-PUFA | 5-HEPE | 317.2 | 115.1 | -80 | -10 | -18 | -6 | $^2$H$_8$-15-HETE | 19.18 | 0.03 | 0.06 | 295 | Koch (2020) Talanta |
| | | 8-HEPE | 317.2 | 155.2 | -80 | -10 | -18 | -8 | $^2$H$_8$-15-HETE | 18.41 | 0.03 | 0.06 | 300 | Koch (2020) Talanta |
| | | 9-HEPE | 317.2 | 167.0 | -70 | -10 | -17 | -8 | $^2$H$_8$-15-HETE | 18.41 | 0.1 | 0.25 | 500 | Koch (2020) Talanta |
| | | 11-HEPE | 317.2 | 167.0 | -70 | -10 | -19 | -8 | $^2$H$_8$-15-HETE | 18.16 | 0.031 | 0.062 | 310 | Koch (2020) Talanta |
| | | 12-HEPE | 317.2 | 179.2 | -85 | -10 | -18 | -8 | $^2$H$_8$-15-HETE | 18.58 | 0.05 | 0.1 | 500 | Koch (2020) Talanta |
| | | 15-HEPE | 317.2 | 219.2 | -80 | -10 | -18 | -10 | $^2$H$_8$-15-HETE | 18.04 | 0.05 | 0.1 | 500 | Koch (2020) Talanta |
| | | 18-HEPE | 317.2 | 259.2 | -75 | -10 | -15 | -7 | $^2$H$_8$-15-HETE | 17.30 | 0.05 | 0.1 | 500 | Koch (2020) Talanta |
| | | 19-HEPE | 317.2 | 229.3 | -70 | -10 | -16 | -8 | $^2$H$_8$-15-HETE | 16.69 | relative quantification based on 18-HEPE | | | Rund (2018) Analytica Chimica Acta |
| | | 20-HEPE | 317.2 | 287.3 | -70 | -10 | -18 | -8 | $^2$H$_6$-20-HETE | 16.76 | 0.25 | 0.50 | 1000 | current paper |
| | multihydroxy-PUFA | 5(*S*),12(*R*),18(*R*)-TriHEPE (RvE1) | 349.3 | 195.0 | -80 | -10 | -22 | -10 | $^2$H$_5$-7(*S*),16(*R*),17(*S*) -TriHDHA (RvD2) | 6.25 | 0.25 | 0.50 | 1000 | current paper |
| | | 5,12,12-TriHEPE (*trans*-RvE1) | 349.2 | 195.0 | -80 | -10 | -22 | -10 | $^2$H$_5$-7(*S*),16(*R*),17(*S*) -TriHDHA (RvD2) | 6.03 | relative quantification based on 5(*S*),12(*R*),18(*R*)-TriHEPE (RvE1) | | | compound e/f from Kutzner (2020) BBA Lipids |
| | | 5(*S*),18(*R*)-DiHEPE (RvE2) | 333.2 | 253.3 | -80 | -10 | -19 | -9 | $^2$H$_4$-LTB$_4$ | 11.27 | 4.63 | 9.26 | 1852 | current paper |
| | | 17(*R*),18(*R*)-DiHEPE (RvE3) | 333.2 | 201.3 | -80 | -10 | -19 | -9 | $^2$H$_4$-9,10-DiHOME | 13.17 | relative quantification based on 8,15-DiHETE | | | Kutzner (2019) Frontiers in Pharmacology |
| | | 17(*R*),18(S)-DiHEPE (18(*S*)-RvE3) | 333.2 | 201.3 | -80 | -10 | -19 | -9 | $^2$H$_4$-9,10-DiHOME | 12.56 | relative quantification based on 8,15-DiHETE | | | Kutzner (2019) Frontiers in Pharmacology |
| | | 5(*S*),15(*S*)-DiHEPE (RvE4) | 333.2 | 115.0 | -80 | -10 | -19 | -7 | $^2$H$_4$-LTB$_4$ | 11.85 | 0.25 | 0.50 | 1000 | current paper |
| | | 5(*S*),6(*R*),15(*S*)-TriHEPE (LxA$_5$) | 349.1 | 215.0 | -70 | -10 | -24 | -13 | $^2$H$_5$-5(*S*),6(*R*),15(*S*)-TriHETE (LxA$_4$) | 8.77 | 1.00 | 2.50 | 1000 | current paper |
| | | LTB$_5$ | 333.3 | 195.2 | -80 | -10 | -21 | -8 | $^2$H$_4$-LTB$_4$ | 11.95 | 0.25 | 0.50 | 1000 | current paper |
| | | 5,12-diHEPE | 333.2 | 195.0 | -80 | -10 | -19 | -9 | $^2$H$_4$-LTB$_4$ | 12.32 | relative quantification based on 5(*S*),15(*S*)-DiHEPE (RvE4) | | | compound 8/9 from Kutzner (2020) BBA Lipids |
| | | 12,18-diHEPE | 333.2 | 179.0 | -80 | -10 | -19 | -9 | $^2$H$_4$-LTB$_4$ | 11.08 | relative quantification based on 5(*S*),18(*R*)-DiHEPE (RvE2) | | | compound c/d from Kutzner (2020) BBA Lipids |
| | | 5,x,18-triHEPE 1 | 349.2 | 215.0 | -80 | -10 | -22 | -10 | $^2$H$_5$-5(*S*),6(*R*),15(*S*)-TriHETE (LxA$_4$) | 7.92 | relative quantification based on 5(*S*),6(*R*),15(*S*)-TriHEPE (LxA$_5$) | | | compound i from Kutzner (2020) BBA Lipids |
| | | 5,x,18-triHEPE 2 | 349.3 | 145.0 | -80 | -10 | -22 | -10 | $^2$H$_5$-5(*S*),6(*R*),15(*S*)-TriHETE (LxA$_4$) | 8.4 | relative quantification based on 5(*S*),6(*R*),15(*S*)-TriHEPE (LxA$_5$) | | | compound h/j from Kutzner (2020) BBA Lipids |
| | epoxy-PUFA | 5(6)-EpETE | 317.2 | 189.1 | -90 | -10 | -16 | -6 | $^2$H$_{11}$-8(9)-EpETrE | 21.56 | relative quantification based on 8(9)-EpETE | | | synthesized using MCPBA as described Newman (2002) J Lipid Res |
| | | 8(9)-EpETE | 317.2 | 127.2 | -90 | -10 | -16 | -6 | $^2$H$_{11}$-8(9)-EpETrE | 21.32 | 0.5 | 0.75 | 500 | Koch (2020) Talanta |
| | | 11(12)-EpETE | 317.2 | 167.0 | -90 | -10 | -16 | -6 | $^2$H$_{11}$-14(15)-EpETrE | 21.14 | 0.1 | 0.25 | 500 | Koch (2020) Talanta |
| | | 14(15)-EpETE | 317.2 | 207.2 | -90 | -10 | -16 | -6 | $^2$H$_{11}$-14(15)-EpETrE | 20.97 | 0.1 | 0.25 | 500 | Koch (2020) Talanta |
| | | 17(18)-EpETE | 317.2 | 215.2 | -90 | -10 | -16 | -6 | $^2$H$_{11}$-14(15)-EpETrE | 20.21 | 0.5 | 0.75 | 500 | Koch (2020) Talanta |
| | | *trans*-5(6)-EpETE | 317.2 | 189.1 | -90 | -10 | -16 | -6 | $^2$H$_{11}$-8(9)-EpETrE | 21.71 | relative quantification based on 8(9)-EpETE | | | Rund (2019) POLM |
| | | *trans*-8(9)-EpETE | 317.2 | 127.2 | -90 | -10 | -16 | -6 | $^2$H$_{11}$-8(9)-EpETrE | 21.47 | relative quantification based on 8(9)-EpETE | | | Rund (2019) POLM |
| | | *trans*-11(12)-EpETE | 317.2 | 167.0 | -90 | -10 | -16 | -6 | $^2$H$_{11}$-14(15)-EpETrE | 21.29 | relative quantification based on 11(12)-EpETE | | | Rund (2019) POLM |
| | | *trans*-14(15)-EpETE | 317.2 | 207.2 | -90 | -10 | -16 | -6 | $^2$H$_{11}$-14(15)-EpETrE | 21.12 | relative quantification based on 14(15)-EpETE | | | Rund (2019) POLM |
| | | *trans*-17(18)-EpETE | 317.2 | 215.2 | -90 | -10 | -16 | -6 | $^2$H$_{11}$-14(15)-EpETrE | 20.36 | relative quantification based on 17(18)-EpETE | | | Rund (2019) POLM |
| | vic hydroxy-PUFA | 5,6-DiHETE | 335.2 | 115.1 | -85 | -10 | -20 | -8 | $^2$H$_4$-LTB$_4$ | 15.43 | 0.23 | 0.3 | 15 | Koch (2020) Talanta |
| | | 8,9-DiHETE | 335.2 | 127.1 | -90 | -10 | -25 | -5 | $^2$H$_4$-LTB$_4$ | 14.68 | 0.05 | 0.100 | 500 | Koch (2020) Talanta |
| | | 11,12-DiHETE | 335.2 | 167.1 | -90 | -10 | -25 | -5 | $^2$H$_4$-LTB$_4$ | 14.27 | 0.025 | 0.05 | 250 | Koch (2020) Talanta |
| | | 14,15-DiHETE | 335.3 | 207.2 | -90 | -10 | -24 | -10 | $^2$H$_4$-LTB$_4$ | 14.04 | 0.025 | 0.05 | 250 | Koch (2020) Talanta |
| | | 17,18-DiHETE | 335.3 | 247.2 | -90 | -10 | -23 | -8 | $^2$H$_4$-LTB$_4$ | 13.44 | 0.083 | 0.11 | 55 | Koch (2020) Talanta |
| | prostanoids | PGB$_3$ | 331.2 | 223.0 | -75 | -10 | -24 | -8 | $^2$H$_4$-PGB$_2$ | 10.40 | 0.5 | 0.75 | 500 | Koch (2020) Talanta |
| | | PGD$_3$ | 349.3 | 269.2 | -80 | -10 | -21 | -6 | $^2$H$_4$-PGD$_2$ | 8.11 | 0.50 | 0.75 | 1000 | current paper |
| | | PGE$_3$ | 349.3 | 269.2 | -80 | -10 | -21 | -6 | $^2$H$_4$-PGE$_2$ | 7.74 | 0.37 | 0.73 | 1462 | current paper |
| | | PGF$_{3\alpha}$ | 351.2 | 193.2 | -95 | -10 | -30 | -7 | $^2$H$_4$-PGF$_{2\alpha}$ | 7.35 | 0.75 | 1 | 250 | Koch (2020) Talanta |
| | | Δ$^{17}$-6-keto-PGF$_{1\alpha}$ | 367.2 | 163.2 | -60 | -10 | -35 | -10 | $^2$H$_4$-6-keto-PGF$_{1\alpha}$ | 5.01 | 0.250 | 0.50 | 500 | Koch (2020) Talanta |
| | | TxB$_3$ | 367.3 | 169.3 | -90 | -10 | -33 | -8 | $^2$H$_4$-TxB2 | 6.54 | 3.23 | 4.30 | 4301 | current paper |
| | | 11-dehydro-TxB$_3$ | 365.3 | 161.2 | -70 | -10 | -24 | -8 | $^2$H$_4$-TxB$_2$ | 7.73 | 0.74 | 1.84 | 737 | current paper |
| | isoprostanes | 15-F$_{3t}$-IsoP (8-iso-PGF$_{3\alpha}$) $^{4)}$ | 351.1 | 193.0 | -95 | -10 | -27 | -8 | $^2$H$_4$-8-iso-PGF$_{2\alpha}$ | 6.45 | 1 | 2.5 | 500 | Koch (2020) Talanta |
| | misc | 12-OH-17(18)-EpETE | 333.1 | 179.3 | -90 | -10 | -17 | -6 | $^2$H$_4$-9,10-DiHOME | 12.74 | relative quantification based on 8,15-DiHETE | | | Rund (2018) Analytica Chimica Acta |
| Docosapentaenoic Acid (DPA; 22:5 *n*-3) | multihydroxy-PUFA | 7(*S*),17(*S*)-DiH-n3DPA | 361.5 | 263.3 | -80 | -10 | -19 | -4 | $^2$H$_4$-LTB$_4$ | 14.00 | 0.5 | 0.75 | 500 | Koch (2020) Talanta |
| | oxo-PUFA | 17-oxo-n3DPA | 343.1 | 247.0 | -85 | -10 | -24 | -8 | $^2$H$_7$-5-oxo-ETE | 22.23 | 2.5 | 5 | 100 | Koch (2020) Talanta |
| Docosahexaenoic Acid (DHA; 22:6 *n*-3) | hydroxy-PUFA | 4-HDHA | 343.2 | 101.1 | -75 | -10 | -17 | -7 | $^2$H$_8$-5-HETE | 22.24 | 0.05 | 0.1 | 500 | Koch (2020) Talanta |
| | | 7-HDHA | 343.2 | 141.2 | -75 | -10 | -17 | -7 | $^2$H$_8$-5-HETE | 21.36 | 0.05 | 0.1 | 500 | Koch (2020) Talanta |
| | | 8-HDHA | 343.2 | 189.2 | -70 | -10 | -17 | -7 | $^2$H$_8$-5-HETE | 21.57 | 0.038 | 0.1 | 190 | Koch (2020) Talanta |
| | | 10-HDHA | 343.2 | 153.2 | -70 | -10 | -19 | -7 | $^2$H$_8$-12-HETE | 20.89 | 0.025 | 0.05 | 500 | Koch (2020) Talanta |
| | | 11-HDHA | 343.2 | 121.1 | -70 | -10 | -18 | -7 | $^2$H$_8$-5-HETE | 21.20 | 0.1 | 0.25 | 500 | Koch (2020) Talanta |
| | | 13-HDHA | 343.2 | 193.2 | -75 | -10 | -17 | -7 | $^2$H$_8$-12-HETE | 20.61 | 0.05 | 0.1 | 500 | Koch (2020) Talanta |
| | | 14-HDHA | 343.2 | 205.2 | -70 | -10 | -17 | -7 | $^2$H$_8$-12-HETE | 20.87 | 0.055 | 0.14 | 275 | Koch (2020) Talanta |
| | | 16-HDHA | 343.2 | 233.2 | -75 | -10 | -17 | -7 | $^2$H$_8$-15-HETE | 20.27 | 0.1 | 0.25 | 500 | Koch (2020) Talanta |
| | | 17-HDHA | 343.2 | 201.2 | -80 | -10 | -18 | -6 | $^2$H$_8$-15-HETE | 20.40 | 0.34 | 0.9 | 170 | Koch (2020) Talanta |
| | | 20-HDHA | 343.2 | 241.2 | -75 | -10 | -17 | -7 | $^2$H$_8$-15-HETE | 19.71 | 0.1 | 0.25 | 500 | Koch (2020) Talanta |
| | | 21-HDHA | 343.2 | 255.2 | -80 | -10 | -16 | -7 | $^2$H$_8$-15-HETE | 19.14 | relative quantification based on 20-HDHA | | | Rund (2018) Analytica Chimica Acta |
| | | 22-HDHA | 343.2 | 313.2 | -85 | -10 | -18 | -7 | $^2$H$_6$-20-HETE | 19.15 | 0.75 | 1.00 | 1000 | current paper |
| | multihydroxy-PUFA | 7(*R*),14(*S*)-DiHDHA (MaR1) | 359.1 | 250.2 | -80 | -10 | -19 | -14 | $^2$H$_4$-LTB$_4$ | 13.60 | 0.75 | 1.00 | 1000 | current paper |
| | | 7(*S*),14(*S*)-DiHDHA (7-*epi*-MaR1) | 359.1 | 250.1 | -80 | -10 | -20 | -5 | $^2$H$_4$-LTB$_4$ | 13.06 | 0.50 | 0.75 | 1000 | current paper |
| | | 13(*R*),14(*S*)-diHDHA (MaR2) | 359.1 | 221.0 | -70 | -10 | -16 | -14 | $^2$H$_4$-9,10-DiHOME | 15.08 | 0.1 | 0.25 | 500 | Koch (2020) Talanta |
| | | 7(*S*),8(*R*),17(*S*)-TriHDHA (RvD1) | 375.3 | 141.0 | -70 | -10 | -19 | -8 | $^2$H$_5$-7(*S*),8(*R*),17(*S*)-TriHDHA (RvD1) | 10.24 | 0.05 | 0.10 | 1000 | current paper |
| | | 7(*S*),8(*R*),17(*R*)-TriHDHA (17(*R*)-RvD1) | 375.3 | 141.0 | -70 | -10 | -19 | -15 | *theoretically* $^2$H$_5$-7(*S*),8(*R*),17(*S*)-TriHDHA (RvD1) $^{5)}$ | 10.35 | relative quantification based on $^2$H$_5$-7(*S*),8(*R*),17(*S*)-TriHDHA (RvD1) | | | current paper |
| | | $^2$H$_5$-7(*S*),8(*R*),17(*S*)-TriHDHA (RvD1) | 380.3 | 141.0 | -80 | -10 | -18 | -8 | *internal standard* | 10.19 | | | | Koch (2020) Talanta |
| | | 7(*S*),16(*R*),17(*S*)-TriHDHA (RvD2) | 375.3 | 141.0 | -80 | -10 | -21 | -8 | $^2$H$_5$-7(*S*),16(*R*),17(*S*) -TriHDHA (RvD2) | 9.45 | 0.75 | 1.00 | 1000 | current paper |
| | | $^2$H$_5$-7(S) ,16(*R*),17(*S*) -TriHDHA (RvD2) | 380.2 | 175.0 | -80 | -10 | -31 | -10 | *internal standard* | 9.40 | | | | Koch (2020) Talanta |
| | | 4(*S*),11(*R*),17(*R*)-TriHDHA (17(*R*)-RvD3) | 375.3 | 147.0 | -80 | -10 | -24 | -10 | *theoretically* $^2$H$_5$-7(*S*),16(*R*),17(*S*) -TriHDHA (RvD2) $^{5)}$ | 9.12 | relative quantification based on 4(*S*),11(*R*),17(*S*)-TriHDHA (RvD3) | | | current paper |
| | | 4(*S*),11(*R*),17(*S*)-TriHDHA (RvD3) | 375.3 | 147.0 | -80 | -10 | -24 | -10 | $^2$H$_5$-7(*S*),16(*R*),17(*S*) -TriHDHA (RvD2) | 9.18 | 0.25 | 0.50 | 1000 | current paper |
| | | 4(*S*),5(*R*),17(*R,S*)-RvD4 | 375.2 | 131.0 | -80 | -10 | -19 | -10 | $^2$H$_5$-RvD1 | 11.43 | 0.1 | 0.25 | 500 | Koch (2020) Talanta |
| | | 7(*S*),17(*S*)-DiHDHA (RvD5) | 359.1 | 199.1 | -70 | -10 | -19 | -10 | $^2$H$_4$-LTB$_4$ | 13.57 | 0.1 | 0.25 | 500 | Koch (2020) Talanta |
| | | 10(*S*),17(*S*)-DiHDHA (PDx) | 359.1 | 153.1 | -80 | -10 | -21 | -10 | $^2$H$_4$-LTB$_4$ | 13.47 | 0.16 | 0.39 | 393 | Koch (2020) Talanta |
| | oxo-PUFA | 4-oxo-DHA | 341.0 | 115.1 | -100 | -10 | -20 | -6 | $^2$H$_7$-5-oxo-ETE | 23.23 | 0.1 | 0.25 | 500 | Koch (2020) Talanta |
| | | 17-oxo-DHA | 341.3 | 111.0 | -85 | -10 | -21 | -8 | $^2$H$_7$-5-oxo-ETE | 22.17 | 7.5 | 10 | 500 | Koch (2020) Talanta |
| | epoxy-PUFA | 4(5)-EpDPE | 343.2 | 229.1 | -75 | -10 | -16 | -7 | $^2$H$_{11}$-8(9)-EpETrE | 23.39 | relative quantification based on 7(8)-EpDPE | | | synthesized using MCPBA as described Newman (2002) J Lipid Res |
| | | 7(8)-EpDPE | 343.2 | 141.2 | -75 | -10 | -16 | -7 | $^2$H$_{11}$-8(9)-EpETrE | 23.08 | 0.44 | 0.65 | 436 | Koch (2020) Talanta |
| | | 10(11)-EpDPE | 343.2 | 153.2 | -90 | -10 | -16 | -7 | $^2$H$_{11}$-8(9)-EpETrE | 22.88 | 0.01 | 0.025 | 500 | Koch (2020) Talanta |
| | | 13(14)-EpDPE | 343.2 | 193.2 | -90 | -10 | -16 | -7 | $^2$H$_{11}$-14(15)-EpETrE | 22.77 | 0.05 | 0.1 | 250 | Koch (2020) Talanta |
| | | 16(17)-EpDPE | 343.2 | 233.2 | -90 | -10 | -16 | -7 | $^2$H$_{11}$-14(15)-EpETrE | 22.68 | 0.1 | 0.25 | 500 | Koch (2020) Talanta |
| | | 19(20)-EpDPE | 343.2 | 241.2 | -90 | -10 | -16 | -7 | $^2$H$_{11}$-14(15)-EpETrE | 22.24 | 0.25 | 0.5 | 500 | Koch (2020) Talanta |
| | | *trans*-4(5)-EpDPE | 343.2 | 229.1 | -75 | -10 | -16 | -7 | $^2$H$_{11}$-8(9)-EpETrE | 23.54 | relative quantification based on 7(8)-EpDPE | | | Rund (2019) POLM |
| | | *trans*-7(8)-EpDPE | 343.2 | 141.2 | -75 | -10 | -16 | -7 | $^2$H$_{11}$-8(9)-EpETrE | 23.23 | relative quantification based on 7(8)-EpDPE | | | Rund (2019) POLM |
| | | *trans*-10(11)-EpDPE | 343.2 | 153.2 | -90 | -10 | -16 | -7 | $^2$H$_{11}$-8(9)-EpETrE | 23.03 | relative quantification based on 10(11)-EpDPE | | | Rund (2019) POLM |
| | | *trans*-13(14)-EpDPE | 343.2 | 193.2 | -90 | -10 | -16 | -7 | $^2$H$_{11}$-14(15)-EpETrE | 22.92 | relative quantification based on 13(14)-EpDPE | | | Rund (2019) POLM |
| | | *trans*-16(17)-EpDPE | 343.2 | 233.2 | -90 | -10 | -16 | -7 | $^2$H$_{11}$-14(15)-EpETrE | 22.83 | relative quantification based on 16(17)-EpDPE | | | Rund (2019) POLM |
| | | *trans*-19(20)-EpDPE | 343.2 | 241.2 | -90 | -10 | -16 | -7 | $^2$H$_{11}$-14(15)-EpETrE | 22.39 | relative quantification based on 19(20)-EpDPE | | | Rund (2019) POLM |
| | vic dihydroxy-PUFA | 4,5-DiHDPE | 361.2 | 229.3 | -90 | -10 | -23 | -6 | $^2$H$_{11}$-11,12-DiHETrE | 19.19 | relative quantification based on 7,8-DiHDPE | | | Rund (2018) Analytica Chimica Acta |
| | | 7,8-DiHDPE | 361.2 | 113.1 | -90 | -10 | -23 | -6 | $^2$H$_{11}$-11,12-DiHETrE | 17.91 | 0.25 | 0.5 | 500 | Koch (2020) Talanta |
| | | 10,11-DiHDPE | 361.2 | 153.2 | -90 | -10 | -23 | -6 | $^2$H$_{11}$-11,12-DiHETrE | 17.03 | 0.05 | 0.1 | 250 | Koch (2020) Talanta |
| | | 13,14-DiHDPE | 361.2 | 193.2 | -90 | -10 | -23 | -6 | $^2$H$_{11}$-11,12-DiHETrE | 16.63 | 0.05 | 0.1 | 500 | Koch (2020) Talanta |
| | | 16,17-DiHDPE | 361.2 | 233.2 | -90 | -10 | -23 | -6 | $^2$H$_{11}$-11,12-DiHETrE | 16.34 | 0.05 | 0.1 | 500 | Koch (2020) Talanta |
| | | 19,20-DiHDPE | 361.2 | 273.2 | -90 | -10 | -23 | -6 | $^2$H$_{11}$-11,12-DiHETrE | 15.71 | 0.25 | 0.5 | 500 | Koch (2020) Talanta |
| Adrenic acid (AdA; 22:4 *n*-6) | prostanoids | 1a,1b-dihomo-PGF$_{2\alpha}$ $^{4)}$ | 381.4 | 221.1 | -80 | -10 | -37 | -10 | $^2$H$_4$-PGB$_2$ | 10.89 | 0.5 | 0.75 | 500 | Koch (2020) Talanta |
| secondary internal standard | | | | | | | | | | | | | | |
| | | Aleuritic Acid | 303.1 | 268.8 | -75 | -10 | -41 | -10 | *secondary internal standard* | 5.39 | | | | Koch (2020) Talanta |
| | | 12-[[(tricyclo[3.3.1.13,7]dec-1-ylamino)carbonyl]amino]-dodecanoic acid (AUDA) | 391.0 | 240.1 | -100 | -10 | -25 | -10 | *secondary internal standard* | 19.66 | | | | Koch (2020) Talanta |
| | | 12-oxo Phytodienoic acid (OPDA) | 291.1 | 165.0 | -90 | -10 | -27 | -10 | *secondary internal standard* | 15.56 | | | | Koch (2020) Talanta |
| | | (1-(1-(Ethyl-sulfonyl)piperidin-4-yl)-3-(4-(trifluoromethoxy)phenyl)urea) 2213 | 394.0 | 176.0 | -100 | -10 | -21 | -10 | *secondary internal standard* | 10.69 | | | | Koch (2020) Talanta |

## Legend

1) limit of detection (LOD) set to lowest concentration with a signal to noise ratio ≥3

2) lower limit of quantification (LLOQ) set to lowest calibration standards with a signal to noise ratio ≥5 and accuracy ±20%

3) upper limit of quantification (ULOQ) set to calibration of the highest injected standard

4) other isoprostanes, isofuranes and phytoprostanes can be included as described in Rund et al., 2017 (Anal Chim Acta 1037, 63-74)

5) preparation of independent calibration necessary due to interferences

## Abbreviations

| | |
|---|---|
| **AdA** | adrenic acid |
| **ALA** | alpha-linolenic acid |
| **ARA** | arachidonic acid |
| **CE** | collision energy |
| **CXP** | collision cell exit potential |
| **DGLA** | dihomo-gamma linolenic acid |
| **DHA** | docosahexaenoic acid |
| **DiH** | dihydroxy |
| **DiHDHA** | dihydroxydocosahexaenoic acid |
| **DiHDPE** | dihydroxydocosapentaenoic acid |
| **DiHEPE** | dihydroxyeicosapentaenoic acid |
| **DiHETE** | dihydroxyeicosatetraenoic acid |
| **DiHETrE** | dihydroxyeicosatrienoic acid |
| **DiHODE** | dihydroxyoctadecadienoic acid |
| **DiHOME** | dihydroxyoctadecamonoenoic acid/ dihydroxyoctadecenoic acid |
| **DP** | declustering potential |
| **DPA** | docosapentaenoic acid |
| **EKODE** | epoxy-keto-octadecadienoic acid |
| **Ep** | epoxy |
| **EP** | entrance potential |
| **EPA** | eicosapentaenoic acid |
| **EpDoTrE** | epoxydocosatrienoic acid |
| **EpDPE** | epoxydocosapentaenoic acid |
| **EpEDE** | epoxyeicosadienoic acid |
| **EpETE** | epoxyeicosatetraenoic acid |
| **EpETrE** | epoxyeicosatrienoic acid |
| **EpETrE** | epoxyeicosatrienoic acid |
| **EpODE** | epoxyoctadecadienoic acid |
| **EpOME** | epoxyoctadecamonoenoic acid/ epoxyoctadecenoic acid |
| **ETE** | eicosatetraenoic acid |
| **FA** | fatty acid |
| **GLA** | gamma-linolenic acid |
| **HDHA** | hydroxydocosahexaenoic acid |
| **HEPE** | hydroxyeicosapentaenoic acid |
| **HETE** | hydroxyeicosatetraenoic acid |
| **HETrE** | hydroxyeicosatrienoic acid |
| **HHTrE** | hydroxyheptatrienoic acid |
| **HOTrE** | hydroxyoctadecatrienoic acid |
| **HpETE** | hydroperoxyeicosatetraenoic acid |
| **IsoP/ iP** | isoprostane |
| **LA** | linoleic acid |
| **LLOQ** | lower limit of quantification |
| **LOD** | limit of detection |
| **LT** | leukotriene |
| **Lx** | lipoxin |
| **MaR** | maresin |
| **ODE** | octadecadienoic acid |
| **Oleic** | oleic acid |
| **OTrE** | octadecatrienoic acid |
| **P** | protectin |
| **PG** | prostaglandin |
| **Rv** | resolvin |
| **TriHDHA** | trihydroxydocosahexaenoic acid |
| **TriHEPE** | trihydroxyeicosapentaenoic acid |
| **TriHETE** | trihydroxyeicosatetraenoic acid |
| **TriHETrE** | trihydroxyeicosatrienoic acid |
| **TriHODE** | trihydroxyoctadecadienoic acid |
| **TriHOME** | trihydroxyoctadecamonoenoic acid/ trihydroxyoctadecenoic acid |
| **Tx** | thromboxane |
| **ULOQ** | upper limit of quantification |

## References


Koch, E., et al., Talanta 2020, 217, 121074.

Kutzner, L., et al., Biochimica et Biophysica Acta (BBA) - Molecular and Cell Biology of Lipids 2020, 1865 (12), 158806.

Kutzner, L., et al., Frontiers in Pharmacology 2019, 10 (169).

Lundström, S. L., et al., PLoS ONE 2011, 6 (8), e23864.

Meckelmann, S. W., et al., Prostaglandins & Other Lipid Mediators 2017, 130, 8-15.

Newman, J. W., et al., Journal of Lipid Research 2002, 43 (9), 1563-1578.

Ostermann, A. I., et al., Anal Bioanal Chem 2015, 407,1403–1414.

Rund, K. M., et al., Analytica Chimica Acta 2018, 1037, 63-74.

Rund, K. M., et al., Prostaglandins Other Lipid Mediators 2019, 144, 106334.

Yang, J., et al., Analytical Chemistry 2009, 81 (19), 8085-8093.

Zivkovic, A. M., et al., Metabolomics 2012, 8 (6), 1102-1113.
